# Carbamoyl phosphate-mediated peptide formation in mild prebiotic environment

**DOI:** 10.1038/s42004-026-02149-6

**Published:** 2026-07-23

**Authors:** Xiangxiao Zheng, Fude Chen, Min Zhang, Shichao Yu, Shuang Xie, Yufen Zhao, Jianxi Ying

**Affiliations:** 1 Institute of Drug Discovery Technology, Ningbo University, Ningbo, P R China; 2 Department of Chemical Biology, College of Chemistry and Chemical Engineering, Xiamen University, Xiamen, P R China; 3 Key Laboratory of Bioorganic Phosphorus Chemistry and Chemical Biology (Ministry of Education), Department of Chemistry, Tsinghua University, Beijing, P R China

**Keywords:** Peptides, Origin of life

## Abstract

The abiotic formation of peptides in water remains a fundamental challenge in origin-of-life research. Direct aqueous condensation of amino acids is thermodynamically hindered; consequently, existing models frequently depend on activation agents or require extreme conditions. We have developed and implemented an aqueous pathway for peptide synthesis mediated by carbamoyl phosphate (CAP) within a mild, “warm little pond” scenario. Co-incubation of diverse proteinogenic amino acids with CAP and metal cations promoted peptide bond formation. Our analysis reveals that magnesium ions operate synergistically with CAP, driving a dual-activation mechanism through *N*-carbamoylation and *O*-phosphorylation intermediates. Crucially, we establish that CAP can be generated in situ directly from early-Earth accessible precursors, urea and orthophosphate. This continuous aqueous pathway entirely bypasses the necessity for destructive dry-state cycles. CAP continues to play a vital role in nitrogen metabolism and the de novo synthesis of pyrimidine nucleotides in extant organisms. Therefore, we suggest that CAP serves as an evolutionary bridge, linking primordial geochemical conditions to the emergence of functional biochemical machinery.

**Figure:**
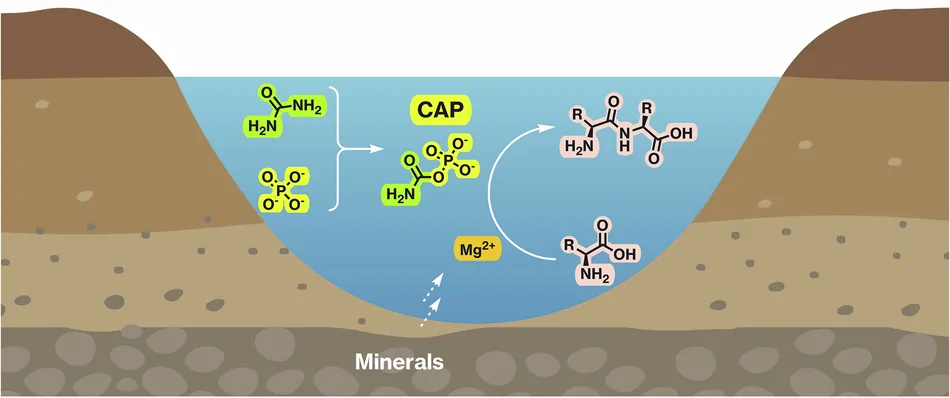

## Abstract (web summary)

The abiotic formation of peptides in water is a key challenge in origin-of-life research due to thermodynamic constraints. Here, the authors demonstrate a mild aqueous pathway for peptide synthesis using carbamoyl phosphate (CAP), revealing a dual-activation mechanism with magnesium ions, suggesting CAP as an evolutionary link to early biochemical processes.

## Introduction

The formation of biological macromolecules, such as proteins, from monomers represents a crucial step in the origin of life^1^. Peptide bonds, the structural foundation of proteins, are formed in modern organisms through the complex synergistic actions of ribosomes, RNAs, and cofactors^2–4^. However, understanding how the first polypeptides emerged before the evolution of these complex molecular machines remains a central challenge. Specifically, the direct condensation of amino acids in an aqueous environment is thermodynamically and kinetically highly unfavorable^5^. Consequently, prebiotic chemistry has frequently turned to alternative pre-activated substrates or exogenous activation reagents. Substrate-driven pathways are initiated from pre-activated derivatives, including aminonitriles^6^, aminoacyl imidazoles^7^, aminoacyl phosphonates^8^, and *N*-carboxyanhydrides (NCAs)^9,10^. Besides, α-hydroxy acids could also form peptide bonds through a combination of ester-amide bond exchange and ester bond hydrolysis^11^. Alternatively, reagent-driven approaches facilitate peptide synthesis by introducing activating agents, such as carbonyl sulfide^9^, 1-ethyl-3-(3-dimethylaminopropyl)carbodiimide (EDC)^12^, and diamidophosphate (DAP)^13,14^.

While these strategies demonstrate the feasibility of abiotic peptide bond formation, challenges regarding the prebiotic availability of these reagents and their environmental plausibility remain significant. To address the thermodynamic barriers of aqueous condensation, research has explored non-equilibrium environments (such as wet–dry cycles^15,16^ and freeze–thaw cycles^17^) or mineral catalysis^18–20^ to concentrate monomers and drive dehydration reactions. In parallel, Darwin’s “warm little pond” concept, proposed in 1871, posits that prebiotic proteins could have originated in shallow water environments rich in ammonia and phosphoric salts, as well as light and heat^21^. This scenario remains a foundational hypothesis in origin-of-life research^21,22^. Within this context, orthophosphate is widely recognized as an indispensable molecule for energy coupling and activation.

Though the scarcity of soluble phosphate on early Earth constitutes a well-known geochemical bottleneck known as the “phosphorus problem,” geological and geochemical studies provide further evidence to address the availability of soluble phosphorus. Current research suggests that soluble phosphate in early Earth’s waters could have had diverse sources, including volcanic activity^23^, hydrothermal activity^24^, and lightning strikes^25^. While these calculated concentrations remain highly debated, ranging from low nanomolar to high micromolar levels^26–28^, they underline the complex and variable geochemical environment of the early Earth.

Furthermore, in the context of the origin of life, urea is considered a key intermediate in important prebiotic synthesis pathways. In particular, its critical roles in phosphorylation and the formation of nucleobases are intimately linked to the environment in which Darwin envisioned the origin of life^22,29,30^. Urea can be synthesized in large quantities from widely accepted prebiotic molecules such as hydrogen cyanide or cyanamide, and is highly water-soluble, allowing it to concentrate in aqueous solutions to form eutectic mixtures^29,30^.

To facilitate amino acid condensation under such a “warm little pond” scenario, appropriate activation methods are essential. Carbamoyl phosphate (CAP) is considered an activated form of prebiotic soluble orthophosphate and has been shown to promote a variety of prebiotic phosphorylation reactions for imidazole, nucleosides, or glycerol^23,31^. Notably, CAP is also a key biochemical intermediate linking nitrogen and carbon metabolism in modern organisms, playing a crucial role in pyrimidine and arginine biosynthesis as well as the urea cycle^32^. Recent studies have also further highlighted the potential of CAP as a promising prebiotic agent for *N*-carbamoylation^33^. However, there has been insufficient research on the role of CAP in prebiotic amino acid condensation. Therefore, this work focuses on the CAP-mediated synthesis of prebiotic peptides and discusses how this process might have proceeded within a “warm little pond”-like scenario. Furthermore, we demonstrate that the synergistic interaction of phosphate, urea, and metal ions could facilitate the condensation of amino acid monomers through the in situ formation of CAP.

## Results and discussion

### CAP promotes peptide synthesis under mild conditions

**Fig. 1:**
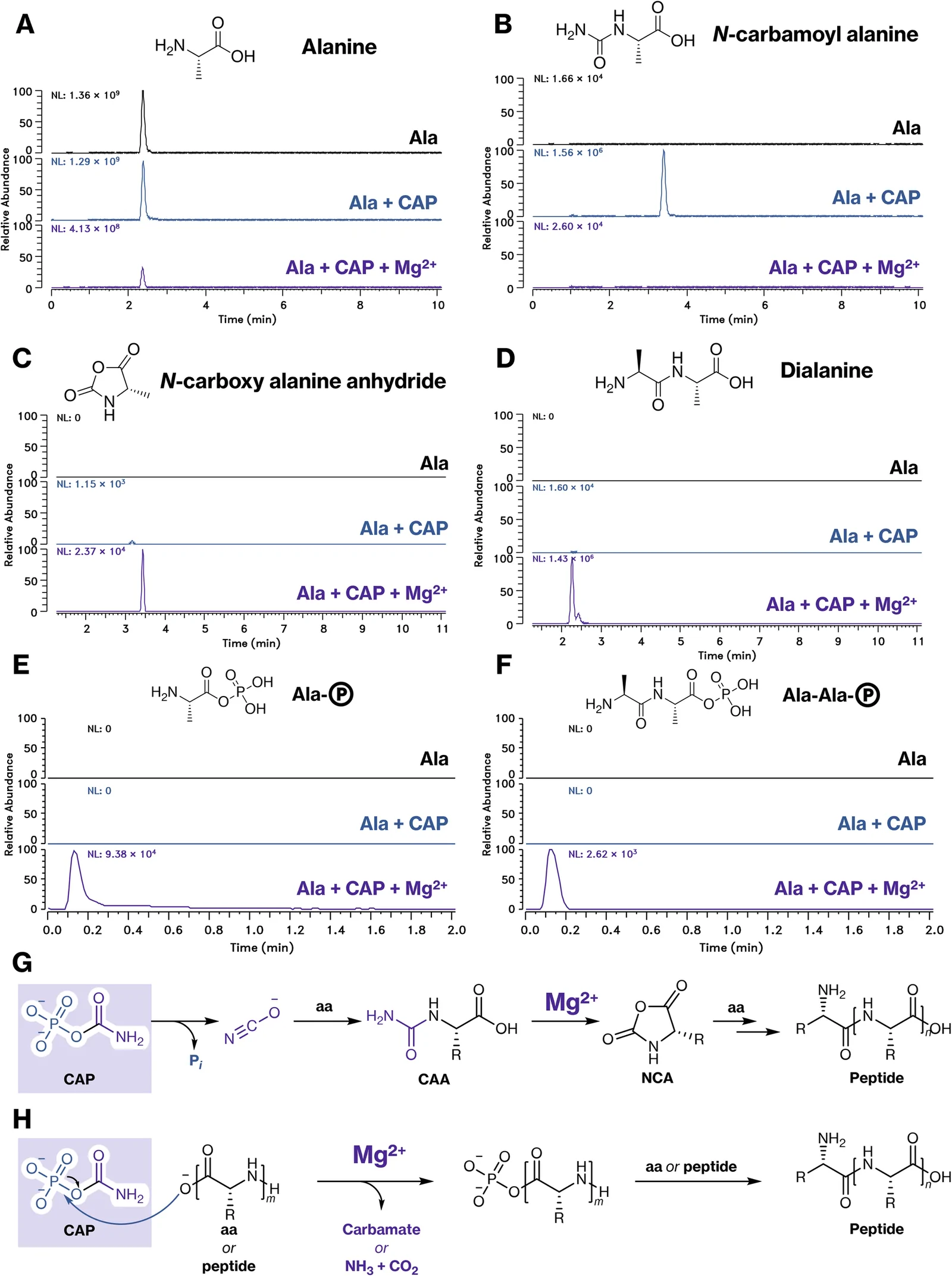
Carbamoyl phosphate (CAP)-mediated peptide bond formation. **A**–**D** Analysis by HPLC-MS in positive ion mode of an aqueous solution of 0.02 m alanine alone (Ala), with 1 equivalent of CAP (Ala + CAP), or with 1 equivalent of CAP and a catalytic amount of Mg^2+^ (Ala + CAP + Mg^2+^), after 5 days of reaction at 37 °C. Extracted ion chromatogram (EIC) spectra of the reactants alanine (Ala), *N*-carbamoylalanine (CAA), *N*-carboxyalanine anhydride (NCA), and dialanine (Ala_2_) are shown. **E**, **F** Analysis by HPLC-MS in negative ion mode of an aqueous solution of 0.02 m alanine alone (Ala), with 1 equivalent of CAP (Ala + CAP), or with 1 equivalent of CAP and a catalytic amount of Mg^2+^ (Ala + CAP + Mg^2+^), after 5 days of reaction at 37 °C. EIC spectra of the phosphorylated products *O*-phosphorylated alanine (Ala-P) and *O*-phosphorylated dialanine (Ala-Ala-P) are shown. **G** CAP mediates the formation of peptide bonds from amino acids via the *N*-carbamoylation pathway through CAA and NCA intermediates. **H** CAP mediates the formation of peptide bonds from amino acids via the *O*-phosphorylation pathway through mixed anhydride intermediates.

Aqueous solutions of alanine (Ala, 0.02 m) were prepared and incubated at 37  °C. The initial pH was set to 6, and no buffer was used to better simulate prebiotic conditions. Since CAP could act as both a phosphorylation and a carbamoylation reagent, to examine the reactivity of CAP in this system, 1 equivalent of CAP was added to co-incubate with Ala. The products were separated and analyzed by high-performance liquid chromatography-mass spectrometry (HPLC-MS) after a 5-day reaction period. In the reaction system to which CAP was added, alanine levels decreased (Fig. 1A), and *N*-carbamoyl alanine was detected (Fig. 1B, *m*/*z*_[M+H]⁺_ = 133.0608; *r.t*. = 3.41 min), which was absent in the control group without CAP. This indicates that CAP can induce the *N*-carbamoylation of amino acids in this setting. Consistently, *N*-carbamoyl alanine can be obtained from Ala with cyanate, as reported by Taillades et al.^34^. Furthermore, considering that CAP primarily exists in the form of a monovalent or divalent anion when pH is around 6, whereas Ala exists mainly as a zwitterion, the concentration of the free amino group is extremely low, rendering it insufficiently nucleophilic to attack CAP. We therefore speculate that cyanate, a potential decomposition product of CAP, could be the key intermediate in the CAP-induced *N*-carbamoylation step. However, no phosphorylated alanine was detected in the Ala + CAP binary system; furthermore, no peptide products were detected regardless of whether CAP was present or not. Notably, weak CAP signals were still observed via ^31^P-NMR monitoring after 5 days of incubation, indicating that CAP exhibits greater stability in our specific prebiotic reaction system than the 92% decomposition rate within 72 h reported by Ter-Ovanessian et al. for neutral, pure CAP solutions^35^ (Fig. S1).

Considering that some metal ions act as essential cofactors in biochemical reactions and were likely present in prebiotic aqueous environments due to mineral dissolution, we introduced catalytic amounts of metal ions into our reaction system^36–38^. Under identical conditions, the addition of Mg^2+^ (Ala + CAP + Mg^2+^) enabled the formation of a dipeptide (Fig.1D, Ala_2_, *m*/*z*_[M+H]⁺_ = 161.0921; *r.t*. = 2.24 min), yielding a concentration of 8.48 μm (Fig. S2), which corresponds to a yield of 0.0424% based on the initial monomer concentration. The conditions for peptide formation are unfavorable in prebiotic aquatic environments, yielding low production rates. Our experiments aim to identify an effective pathway for prebiotic peptide synthesis by simulating the early Earth’s conditions^39^. Furthermore, phosphorylated alanine was also detected in systems containing Mg^2+^ (Fig. 1B). Under weakly acidic conditions, this phosphorylated amino acid is more likely to be an *O*-phosphorylated product formed by the deprotonated carboxyl group of alanine attacking the phosphorus atom of CAP, while Mg^2+^ has been shown to stabilize phosphorylated amino acids and increase peptide yield in triphosphate reaction systems^40^.

Mechanistically, CAP possesses dual reactivity, capable of driving both carbamoylation and phosphorylation. Previous work by Danger et al.^10^ demonstrated that *N*-carbamoyl amino acids (CAAs) may serve as activated forms of amino acids in prebiotic chemistry, forming NCAs via intramolecular cyclization, which can readily polymerize to form peptides^41^. In the absence of Mg^2+^, a signal for *N*-carbamoyl alanine was detected in the system. In contrast, the addition of Mg^2+^ not only promoted the formation of the dipeptide Ala_2_ but also induced distinct signals corresponding to *N*-carboxy alanine anhydride (Fig. 1C), a key intermediate in the CAA-initiated peptide chain elongation, and *O*-phosphoryl alanine (Fig. 1E). Phosphorylated dipeptide products were also detected in the product mixture from this reaction system (Fig. 1F). Therefore, the presence of Mg^2+^ promotes the conversion of CAA to peptide products via NCA in aqueous solutions under milder conditions. Specifically, this process proceeds at significantly lower temperatures and within shorter time frames compared to the reaction at 140 °C reported by Mita et al.^42^ or at 80 °C for over 28 days reported by Danger et al.^10^ (Fig. 1G). In addition, Mg^2+^ is proposed to stabilize *O*-phosphoryl alanine, a highly reactive mixed acid anhydride, thereby facilitating the formation of the peptide bond (Fig. 1H).

**Fig. 2:**
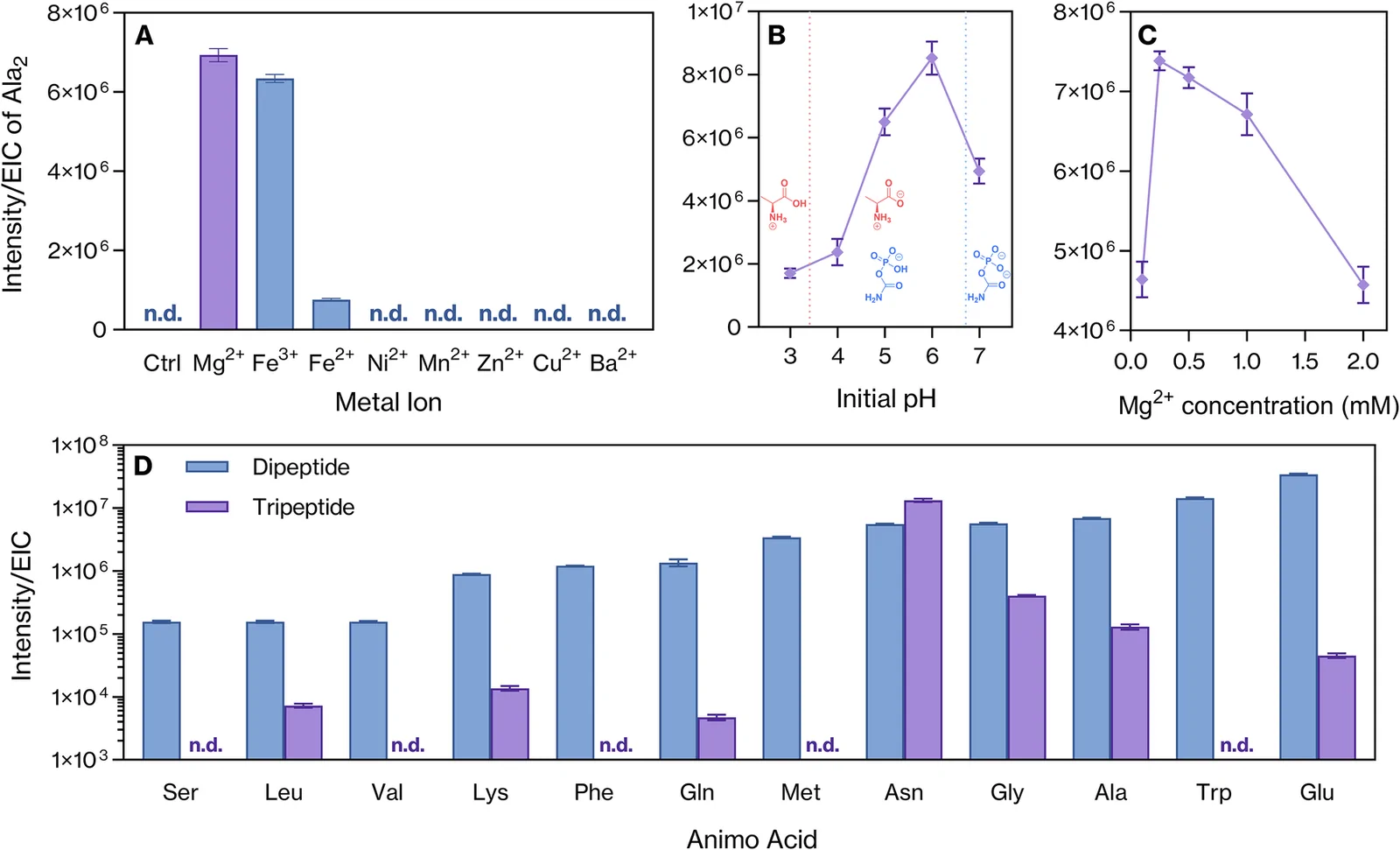
Effects of metal ion type, concentration, and pH on CAP-mediated peptide formation processes. **A** EIC intensity of Ala_2_ in an Ala-CAP binary system with Mg^2+^ or seven transition metal ions. Peptide bonds can only form when Mg^2+^, Fe^2+^, or Fe^3+^ are present. **B** EIC intensity of Ala_2_ in an Ala-CAP binary system with 0.25 mm Mg^2+^ at an initial pH range from 3 to 7. The protonation states of the two key reactants, Ala and CAP, within the tested pH range are also shown. **C** EIC intensity of Ala_2_ in an Ala-CAP binary system with Mg^2+^ (at concentrations of 0.1 mm, 0.25 mm, 0.5 mm, 1.0 mm, or 2.0 mm). **D** EIC intensity of dipeptides and tripeptides formed when each of the 12 amino acids reacts with CAP in the presence of 0.25 mm Mg^2+^. Error bars represent mean ± SD (*n* = 3 independent experiments). *n.d.: peptide product not detected.

We subsequently assessed the catalytic efficacy of various metal ions. When Mg^2+^ was replaced by other metal ions, only Fe^2+^ and Fe^3+^ could promote Ala_2_ formation, with lower yields (Figs. 2A and S3–S5). This can be attributed to the ability of Mg^2+^ to simultaneously bind one molecule of CAP and one molecule of Ala, forming a [CAP-Mg^2+^-Ala] ternary transition-state complex. This reduces the spatial distance between the reactants, and the positive charge of Mg^2+^ also polarizes the P–O bond, thereby lowering the activation energy required for the carboxyl group of alanine to attack the phosphorus atom and form *O*-phosphoryl alanine. Although Fe^3+^ and Mg^2+^ exhibit better binding and catalytic efficiency with acylphosphates in the aqueous phase compared to other metals^43^, Fe^3+^ tends to form precipitates at higher pH levels and may trigger oxidative side reactions. In addition, under the ambient air atmosphere employed in our experiments, the majority of the initially added Fe^2+^ was likely oxidized to Fe^3+^ by the end of the reaction.

Furthermore, neither Ba^2+^ nor Mn^2+^ alone resulted in the formation of the peptide, whereas the reaction proceeded successfully when both ions were present simultaneously (Figs. S6–S8). This phenomenon indicates a significant bimetallic synergistic effect in the system rather than simple single-metal-ion promotion. A similar bimetallic synergism has been extensively discussed in biochemistry and in prebiotic chemical systems^44,45^. Ba^2+^ typically stabilizes polyanionic substrates or intermediates through charge shielding and coordination interactions, thereby lowering the conformational and electrostatic energy barriers of reaction precursors. In contrast, Mn^2+^ predominantly functions as a Lewis acid during critical steps, participating in bond polarization and transition state stabilization to lower the activation free energy^44^. Consequently, the coexistence of both metal ions alters the reaction pathway, rendering the otherwise kinetically prohibitive process feasible through a distinct bimetallic synergistic mechanism.

Given that Mg^2+^ exhibits the most effective catalytic performance in the test set, pH dependence (within the range of 3–7) for the CAP-Mg^2+^-mediated reaction was further investigated. The optimal pH for this system was determined to be around 6 based on the relative yields of dipeptide (Ala_2_) across the different groups (Figs. 2B and S9–S13). Based on the p*K*_a_ data proposed in ref. ^46^ and SwissParam computation^47–49^, protonation state analysis showed that CAP exists predominantly as a monovalent anion and Ala as a zwitterion between pH 3.4 and 6.7. This favorable distribution of ionic species optimizes the reactive centers of CAP, making the phosphorus atom more electrophilic than its divalent anion form, thereby facilitating concurrent *N*-carbamoylation and phosphorylation (Fig. 1F, G). Despite this theoretically favorable protonation window stretching down to pH 3.4, the Ala_2_ yield declines as the pH decreases below 6. This led us to speculate that more acidic environments suppress the reaction via distinct mechanisms. On the one hand, CAP tends to undergo spontaneous degradation in these lower pH environments. On the other hand, a downward pH shifts the zwitterionic alanine toward its fully protonated form, whereby its diminished nucleophilicity becomes insufficient to attack CAP and drive the subsequent coupling processes effectively. In addition, a slight pH elevation was observed after each reaction set, which may be attributed to the generation of soluble ammonia during the process.

The formation of Ala_2_ was sensitive to the concentration of Mg^2+^. Specifically, the yield of Ala_2_ increased with escalating Mg^2+^ concentrations up to an optimal window of 0.25–1.0 mm, beyond which higher Mg^2+^ concentrations led to a subsequent decline (Figs. 2C and S14–S18). At low concentrations, Mg^2+^ can bind CAP and Ala in an ideal stoichiometric ratio, forming a ternary bridged complex that drives the reaction forward. In contrast, at excessively high Mg^2+^ concentrations, CAP and Ala no longer share the same metal center; instead, their independent coordination with separate metal ions induces electrostatic repulsion when they come into close spatial proximity, thereby hindering the formation of critical reactive intermediates. Furthermore, high concentrations of Mg^2+^ and inorganic phosphate gradually released from CAP could form microscopic, insoluble magnesium phosphate clusters, reducing the local concentration of effective reactants.

To evaluate the amino acid universality of this prebiotic peptide synthesis pathway, we investigated the reactions of various proteinogenic amino acids under optimized conditions, with an initial pH of 6 and 0.25 mm Mg^2+^. In all amino acid reaction systems (including aliphatic, aromatic, polar uncharged, basic, and acidic amino acids), the corresponding dipeptide and tripeptide products were detected (Figs. 2D and S19–S34). Among the amino acids tested, asparagine (Asn) produced a higher tripeptide signal than the corresponding dipeptide product. However, this enhanced intensity does not necessarily equate to a higher absolute chemical yield, which may be partly attributed to differences in ionization efficiency during mass spectrometry detection caused by the amide group on its side chain. However, the corresponding peptide products obtained from these amino acids after the reaction were no longer than tripeptides. Given that different amino acids or peptide products have varying ionization efficiencies, the EIC signals of these peptide products are used only for relative quantification. Simultaneously, the formation of diketopiperazine (DKP), a well-known thermodynamic trap, was detected in the systems (Figs. S35–S40), accompanied by the detection of the corresponding CAA, NCA, and *O*-phosphoryl amino acid intermediates (Figs. S41–S61). Consequently, due to the inherent differences in ionization efficiencies among diverse peptide species in HPLC-MS, the EIC signals presented in Fig. 2D are only for semi-quantitative and relative comparison purposes.

Since cyanate is a widely recognized carbamoylating agent in prebiotic chemistry^10^ and is also one of the hydrolysis products of CAP, we compared the effects of potassium cyanate (KCNO) and CAP on the condensation of 12 amino acids under identical conditions. Compared to the direct use of cyanate, the CAP-mediated system not only produced significantly more peptides but also promoted the formation of longer peptide chains (Figs. 2D, S28–S34, and S62). Furthermore, previous studies on cyanate-mediated prebiotic peptide synthesis were often conducted under relatively extreme conditions. Flores and Leckie observed cyanate-mediated ligation at temperatures exceeding 95 °C^50^. Taillades et al. achieved the *N*-carbamoylation of amino acids using cyanate under milder conditions (pH 7–8, 40–50 °C), but did not report peptide formation^34^. Similarly, Danger et al. started from *N*-carbamoylated precursors to form peptides, yet the reaction required prolonged incubation (for at least 28 days) even at 80 °C^10^.

These results indicate that under relatively mild conditions, CAP can promote the condensation of amino acids in the presence of metal ions, proceeding via parallel CAP-mediated *N*-carbamoylation and *O*-phosphorylation pathways.

### Potential sources of CAP in prebiotic ponds

Although CAP exhibits amino acid activation properties, its instability and susceptibility to decomposition in aqueous solutions limit its availability in prebiotic scenarios^35^. To address this, we investigated the in situ generation of CAP within the conceptual framework of Darwin’s “warm little pond.” Among the available chemical inventory, urea and inorganic orthophosphate are highly plausible precursors^51^. Although bulk oceanic phosphate was heavily restricted on early Earth due to the precipitation of insoluble minerals (the “phosphorus problem”), evaporating shallow basins could have effectively concentrated localized phosphate and urea reservoirs^23,24^. Within this scenario, while urea is known to form eutectic mixtures and lower water activity in localized regimes^52^, orthophosphate provides the indispensable thermodynamic handle required to forge high-energy bonds^53,54^.

**Fig. 3:**
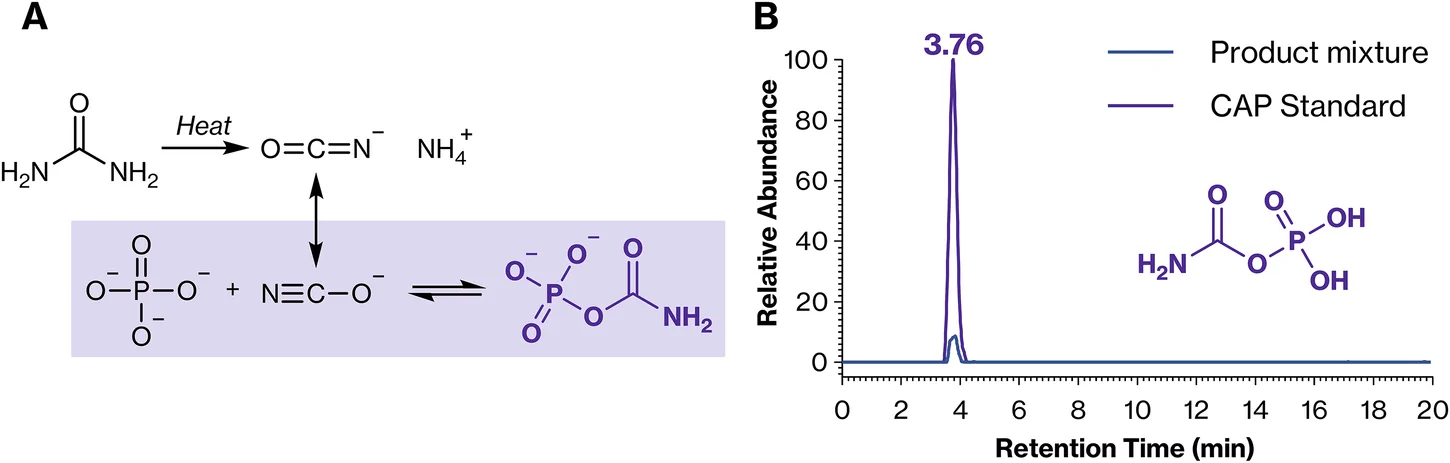
In situ generation of CAP from urea and phosphate. **A** Proposed mechanism of CAP formation. **B** EIC spectrum of the CAP product in a heated urea-phosphate system after 72 h (blue line), and after CAP standard addition (purple line).

To evaluate the feasibility of this in situ CAP synthesis, an aqueous mixture of urea, disodium hydrogen phosphate (Na_2_HPO_4_), and Mg^2+^ was heated to 65 °C for 72 h. Due to the intrinsic instability and rapid hydrolysis kinetics of CAP, achieving its precise absolute quantification within the complex aqueous mixture remains technically challenging. Instead, the successful generation of CAP (*m/z*_[M+H]⁺_ = 163.9720) was qualitatively confirmed via HPLC-MS employing a rigorous standard addition approach (Figs. 3B and S63). In our system, CAP is formed via the reaction between inorganic phosphate and cyanate intermediates (NCO^−^) derived from the thermal decomposition of urea (Fig. 3A)^55^.

### Alternate approach to peptide in the “warm little pond”

**Fig. 4:**
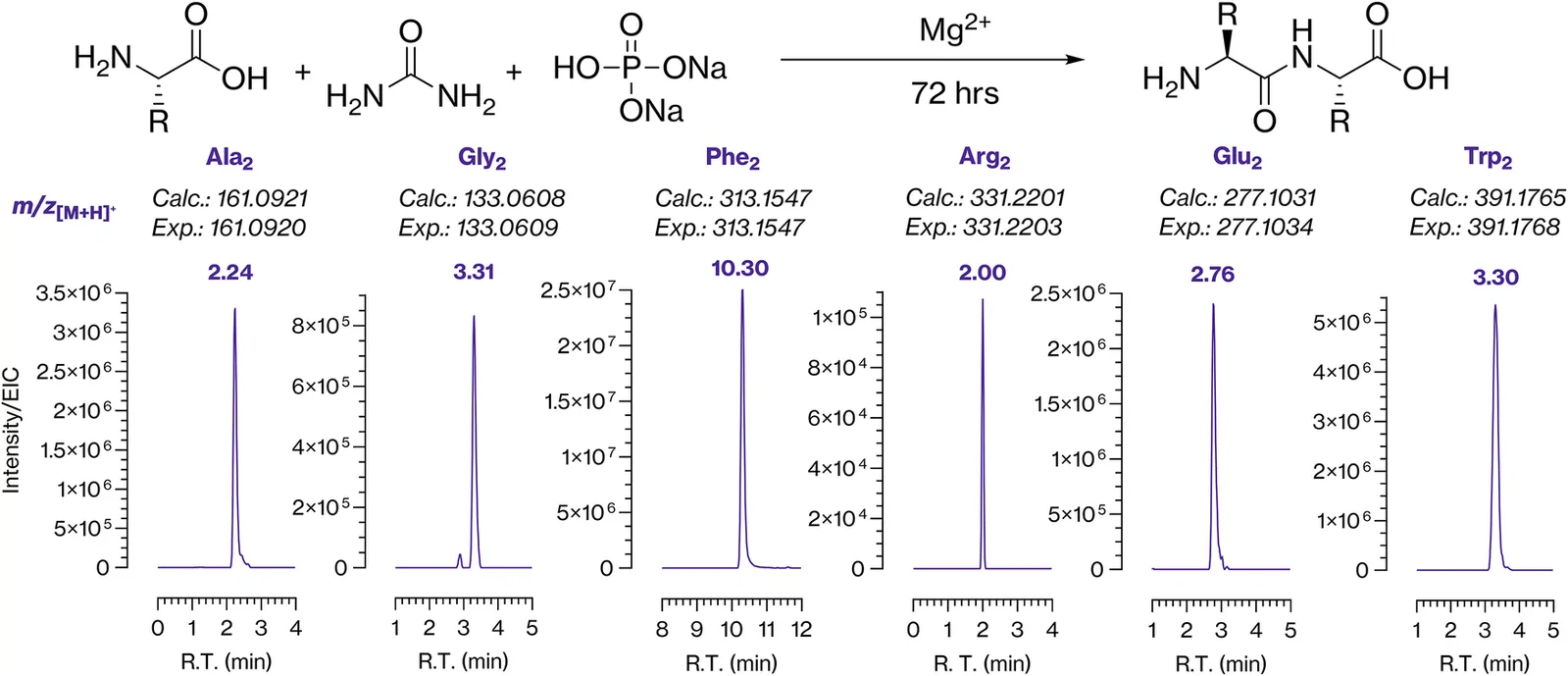
Peptide formation from amino acids in the simulated urea-phosphate lake with Mg^2+^. Extraction ion chromatography of the dipeptide product after 72 h of reaction. Ala, Gly, Phe, Arg, Glu, and Trp were tested, and the corresponding dipeptide products (Ala_2_, Gly_2_, Phe_2_, Arg_2_, Glu_2_, and Trp_2_) were all detected.

To simulate a more prebiotically plausible scenario, where large amounts of pre-synthesized CAP were not present, we replaced the direct addition of CAP with the precursors described previously: soluble orthophosphate (0.01 mmol) and urea (0.1 mmol). The reaction temperature was concurrently lowered to 37 °C. Notably, CAP was indeed detected within this phosphate-and-urea co-incubation system at this physiological temperature (Fig. S64). Upon the introduction of Ala, the resulting mixture was separated and analyzed using HPLC-MS. Consistent with the CAP-mediated pathway, the formation of Ala_2_ was successfully identified within this precursor-driven system (Figs. 4 and S65). In contrast, the exclusion of phosphates from the system suppressed peptide formation under identical conditions (Figs. S66–S68). When the phosphate-depleted system was reacted at 65 °C, peptide formation was observed, but the yield remained lower than under conditions where phosphate was present at 37 °C (Figs. S67 and S68), suggesting that phosphate could facilitate peptide formation under milder conditions. Five other amino acids were also tested, and the corresponding dipeptides and tripeptides were detected in the respective products (Figs. 4 and S69–S77). Similarly, no peptide products larger than trimers were detected in the current system.

To verify the role of each component, a series of control experiments was performed. Control incubation in ultrapure water yielded no peptide products, confirming that amino acids do not undergo spontaneous condensation under ambient aquatic conditions. Furthermore, the omission of any single component, whether orthophosphate, urea, or Mg^2+^, inhibited peptide bond formation, leaving no detectable dipeptides or tripeptides in the corresponding extracted ion chromatograms (Fig. S66 and Table S3). This dependence on all components indicates a mechanism that differs from direct carbamoylation by cyanates or direct phosphorylation by inorganic phosphate. The requirement for orthophosphate confirms that the reaction is driven by transiently generated CAP, which provides a “dual-activation” pathway, thereby enabling peptide elongation within a mild aqueous system and timeframe.

### Conclusion

In this study, we have demonstrated a prebiotically plausible pathway for abiotic peptide bond formation mediated by CAP in mild aqueous environments. We showed that the synergistic interaction between CAP and specific metal ions, particularly Mg^2+^, successfully facilitates the condensation of a diverse range of proteinogenic amino acids into dipeptides and tripeptides. As noted in early studies, CAP acts as a labile intermediate that can decompose into cyanate and phosphate^46^. Cyanate itself has been shown to mediate peptide bond formation, particularly in the presence of hydroxyapatite under hypohydrous conditions^50^. Our process bypasses the need for dry-state conditions, which are often required for cyanate-mediated condensation on mineral surfaces, via a dual-activation mechanism involving *N*-carbamoylation and *O*-phosphorylation intermediates. Furthermore, we established that CAP can be generated in situ from accessible early-Earth precursors, urea and orthophosphate, supporting the chemical viability of Darwin’s “warm little pond” hypothesis.

The CAP-mediated system promotes peptide bond formation under mild aqueous conditions, offering a distinct path compared to other prebiotic activation chemical networks. Trimetaphosphate (TMP), a classic inorganic activator, historically relied on dry-heating regimes or wet-dry fluctuations to drive condensation^56^; however, its capacity to facilitate peptide synthesis and phosphorylation in ambient, non-boiling water across a wide pH range has been increasingly recognized^57^. While the TMP pathway operates via cyclic intermediates, the CAP system presented here provides an additional, plausible pathway for condensation within temperate aqueous settings. Another class of activators, DAP, reacts efficiently with simple aldehydes and hydrogen cyanide in water, yet its prebiotic plausibility on the early Earth remains debated^13^. In contrast, the aminonitrile approach bypasses the thermodynamic barrier associated with free amino acid activation by directly utilizing the high-energy intermediate in the Strecker synthesis. Although this approach achieves peptide chain elongation through H_2_S-mediated pathways or specific catalysis, it relies heavily on a cyanide chemical network and a specific sulfide-enriched environment^58^. Therefore, while these reagents undoubtedly expanded the prebiotic inventory, we suggest that CAP distinguishes itself as a unique evolutionary bridge, structurally integrating both carbamoylation and phosphorylation potentials that modern metabolism has fundamentally retained.

While our results demonstrate the ability of a urea-phosphate-metal system to drive early peptide synthesis, the study also highlights certain limitations; specifically, the current system primarily yields short oligomers and remains susceptible to thermodynamic traps, such as DKP formation. Future research should investigate the coupling of this CAP-mediated activation with non-equilibrium environmental conditions to suppress DKP formation and promote further chain elongation. Finally, these findings advance our fundamental understanding of prebiotic chemistry by showing how a cooperative network of simple molecules could bridge the gap between primordial geochemistry and the origins of functional biochemical machinery.

## Methods

### Peptide formation mediated by CAP and metal ions

CAP (0.02 mmol), amino acids (0.02 mmol), and metal ions (0.0005 mmol) were dissolved in 1.0 mL of ultrapure water under ambient atmosphere. The pH of the reaction solution was adjusted to 6.0 using either 5 M NaOH or 2 M HCl. The mixture was subsequently incubated in a sealed container at 37 °C for 5 days.

### Peptide synthesis in Darwin’s “warm little pond”

Urea (0.1 mmol), Na_2_HPO_4_ (0.01 mmol), metal ions (0.001 mmol), and amino acids (0.02 mmol) were dissolved in 1.0 mL of ultrapure water. The initial pH was adjusted to 6 using 5 m NaOH or 2 m HCl. Subsequently, the mixture was sealed and incubated at 37 °C for 72 h.

### Chelation of metal ions with EDTA

To prevent damage to the chromatographic column by metal ions, the post-reaction samples were treated with EDTA at twice the molar concentration of the metal ions. Following mixing, settling, and centrifugation, the resulting mixture was filtered through a 0.22-μm PES microporous filter and analyzed via HPLC-MS. Furthermore, control experiments confirmed that the addition of EDTA before analysis did not interfere with the mass spectrometry results (Figs. S78–S81).

### HPLC-MS analysis

High-performance liquid chromatography tandem mass spectrometry (HPLC-MS/MS) measurements were performed on a Thermo Scientific™ UltiMate™ 3000 HPLC system and Q Exactive™ Plus Hybrid Quadrupole-Orbitrap™ mass spectrometer.

For positive ion mode analysis, a gradient-type HPLC method was used during each run with ACN/0.1% formic acid as mobile phase A and 95% water/5% ACN/0.1% formic acid as mobile phase B (Table S1). An injection volume of 5 µL of the extract was introduced onto a Luna^®^ C18 column (250 mm × 4.6 mm, particle size 5 µm, Phenomenex Ltd., Germany) held at room temperature with a solvent flow rate of 1.0 mL min^−1^ to drive the chromatographic separation.

For negative ion mode analysis, a gradient-type HPLC method was used during each run with 50 mM ammonium acetate as mobile phase A and acetonitrile as mobile phase B (Table S2). An injection volume of 20 µL of the extract was introduced onto a Hypersil GOLD™ C18 column (250 mm × 4.6 mm, particle size 5 µm, Thermo Scientific, United States) held at room temperature, with a solvent flow rate of 0.8 mL min^−1^ to drive the chromatographic separation. The detection wavelength was set to 260 nm.

Mass measurements were carried out in positive or negative ESI mode, with source gases set at sheath gas = 35 a.u, aux gas = 15 a.u., sweep gas = 0 a.u. The ESI spray voltage was set to 3800 V, and the capillary temperature was set at 320 °C. Mass spectra were registered in the scan range of 50–750 *m/z* or 150–2000 *m/z*.

Mass spectrometry data analysis was performed using Thermo Scientific™ Xcalibur™ 4.7 software.

## Supplementary information

Supplementary Materials

Description of Additional Supplementary Files

Supplementary Data

## Data Availability

The data supporting the findings of this study are available within the paper and its Supplementary Information files. The source data for Fig. 2 are provided in Supplementary Data. All data are available from the corresponding author on request.
