# Supplementary material for: Carbamoyl phosphate-mediated peptide formation in mild prebiotic environment
Source: Commun Chem. 2026 Jul 23;9:361. doi: 10.1038/s42004-026-02149-6 (PMC13760053; doi:10.1038/s42004-026-02149-6)
Supplement: Supplementary file 1 [file 42004_2026_2149_MOESM1_ESM.pdf]

Supplementary Information

for manuscript entitled

Carbamoyl Phosphate-Mediated Peptide Formation in Mild Prebiotic Environment

Xiangxiao Zheng,[a,§] Fude Chen,[a,§] Min Zhang,[a,b] Shichao Yu,[a] Shuang Xie,[a] Yufen Zhao,[a,b,c] and Jianxi Ying[a*]

a. Institute of Drug Discovery Technology, Ningbo University, Ningbo 315211, P R China.

b. Department of Chemical Biology, College of Chemistry and Chemical Engineering, Xiamen University, Xiamen 361005, P R China.

c. Key Laboratory of Bioorganic Phosphorus Chemistry and Chemical Biology (Ministry of Education), Department of Chemistry, Tsinghua University, Beijing 100084, P R China.

§ These authors contributed equally to this work.

* Correspondences: J. Ying: yingjianxi@nbu.edu.cn

Materials and Methods 2
Equipment and Reagents 2
Synthesis method 3
CAP synthesis procedure from Urea and Phosphate 3
General peptide synthesis procedure with CAP 3
The method of chelating metal ions with EDTA 3
Analysis Methods 3
Supplementary Figures and Tables 5
CAP promotes peptide synthesis with metal ions 5
Ba^{2+}, Mn^{2+}, and Cu^{2+} can be combined in pairs to promote peptide formation.. 8
Optimization of pH and Metal Ions 9
CAP mediated amino acid peptide formation 14
Diketopiperazines (DKPs) in products 22
N-Carbamoyl amino acids (CAAs) in products 24
N-carboxyanhydrides (NCAs) in products 29
O-phosphoryl amino acid in products 32
CAP and potassium cyanate (KCNO) promote peptide bond formation 35
CAP can be in situ formed with urea and phosphate 35
One-pot peptide synthesis 36
The influence of EDTA on reaction systems 42
Supplementary Data 45

Materials and Methods

Equipment and Reagents

Proline (Pro), Methionine (Met), Glycine (Gly), Valine (Val), Histidine (His), Isoleucine (Ile), Leucine (Leu), Threonine (Thr), Tryptophan (Trp), Tyrosine (Tyr), Alanine (Ala), Serine (Ser), Phenylalanine (Phe), Arginine (Arg), Asparagine (Asn), Aspartic acid (Asp), Cysteine (Cys), Glutamine (Gln), Glutamic acid (Glu), Lysine (Lys), disodium carbamoyl phosphate (CAP), DNA-Grade EDTA Solution（0.5 M, pH 8.0）, barium chloride ($BaCl_2$), manganese chloride ($MnCl_2$), anhydrous aluminum chloride ($AlCl_3$), magnesium chloride ($MgCl_2$), iron(II) chloride ($FeCl_2$), nickel chloride ($NiCl_2$), iron(III) sulfate ($Fe_2(SO_4)_3$), and disodium hydrogen phosphate (Na_2HPO_4) were obtained from Shanghai Macklin Biochemical Co., Ltd. Urea pellets (≥ 99.5%, Lot#: 705G053) were purchased from Beijing Solarbio Science & Technology Co., Ltd. All amino acids used were of L-configuration. Ultrapure water (18.2 MΩ·cm) was prepared using a HealForce® SMART Plus N Water Purification System.

High-performance liquid chromatography tandem mass spectrometry (HPLC-MS/MS) measurements were performed on a Thermo Scientific™ UltiMate™ 3000 HPLC system and Q Exactive™ Plus Hybrid Quadrupole-Orbitrap™ mass spectrometer. Mass measurements were carried out in positive or negative ESI mode, with source gases set at sheath gas = 35 a.u, aux gas = 15 a.u., sweep gas = 0 a.u. The ESI spray voltage was set to 3800 V, and the capillary temperature was set at 320 °C. Mass spectra were registered in the scan range of 50-750 m/z or 150-2000 m/z. Mass spectrometry data analysis was performed using Thermo Scientific™ Xcalibur™ 4.7 software.

Synthesis method

CAP synthesis procedure from Urea and Phosphate

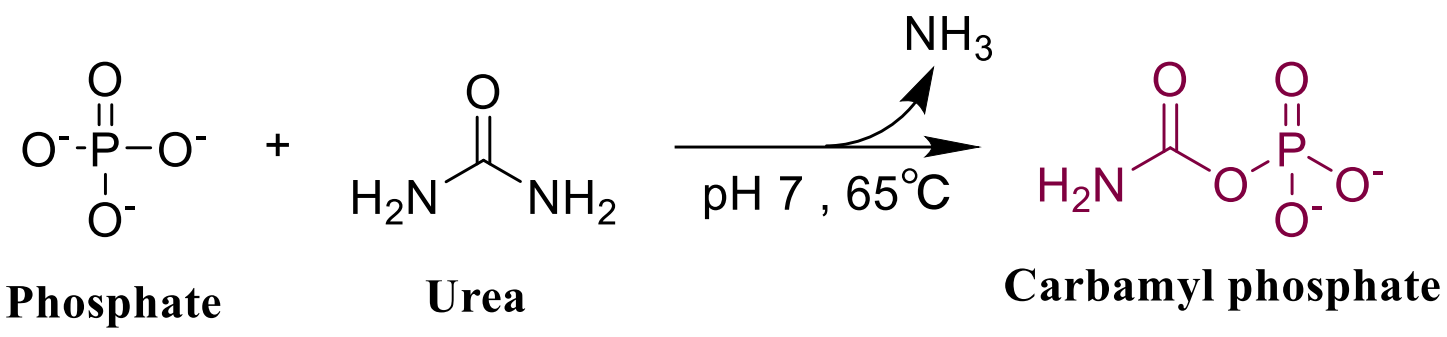

Urea (0.1 mmol), phosphate salts (0.01 mmol), and metal ions (0.001 mmol) were dissolved in ultrapure water (1.0 mL) under ambient atmosphere. The pH of the reaction solution was adjusted to 6.0 using either 5 M sodium hydroxide solution or 2 M hydrochloric acid. The mixture was subsequently heated in a sealed container at 65 °C for 72 hours. Upon completion of the reaction, the resulting mixture was filtered through a 0.22-µm PES syringe filter and subjected to HPLC-MS analysis.

General peptide synthesis procedure with CAP

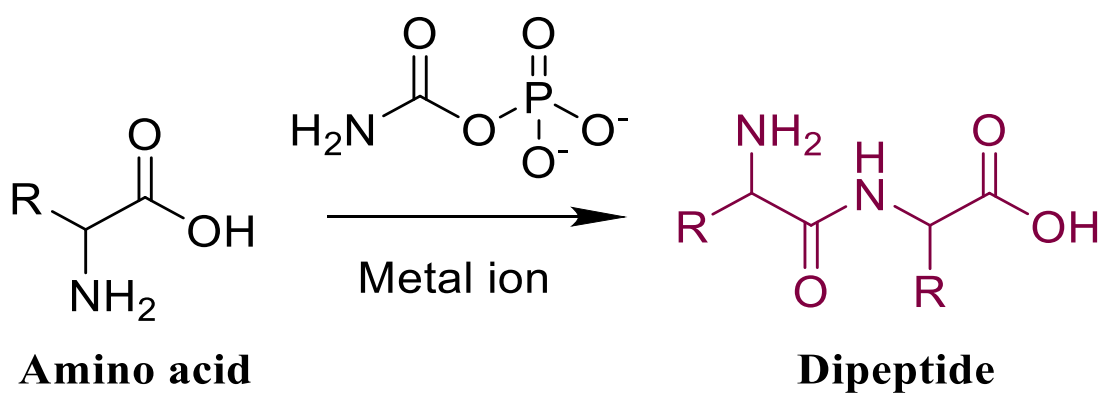

Carbamoyl phosphate (CAP, 0.02 mmol), amino acids (0.02 mmol), and metal ions (0.0005 mmol) were dissolved in ultrapure water (1.0 mL) under ambient atmosphere. The pH of the reaction solution was adjusted to 6.0 using either 5 M sodium hydroxide solution or 2 M hydrochloric acid. The mixture was subsequently incubated in a sealed container at 37 °C for five days. Upon reaction completion, the resulting mixture was filtered through a 0.22-µm PES syringe filter and analyzed by high performance liquid chromatography-mass spectrometry (HPLC-MS).

The method of chelating metal ions with EDTA

To prevent the chromatographic column from being damaged by metal ions, samples containing metal ions are treated by adding EDTA, which has twice the concentration of metal ions, after the reaction is completed for chelation. Then, by mixing, settling and centrifugation, the obtained mixture is filtered through a 0.22-micron PES microporous filter, and analyzed by high-performance liquid chromatography-mass spectrometry (HPLC-MS). EDTA will not have any effect on the signals of amino acids and dipeptides (Figs.S60-S63).

Analysis Methods

High-performance liquid chromatography tandem mass spectrometry (HPLC-MS/MS) measurements were performed on Thermo Scientific™ UltiMate™ 3000 HPLC system and Q Exactive™ Plus Hybrid Quadrupole-Orbitrap™ mass spectrometer.

For positive ion mode analysis, a gradient-type HPLC method was used during each run with ACN/0.1% formic acid as mobile phase A and 95% water/5% ACN/0.1% formic acid as mobile phase B. An injection volume of 5 µL of the extract was introduced onto a Luna® C18

column (250 mm × 4.6 mm, particle size 5 µm, Phenomenex Ltd., Germany) held at room temperature with a solvent flow rate of 1.0 mL min^{-1} to drive the chromatographic separation.

Table S1: Flow Gradient for Loading Pump in HPLC Assay in positive analysis mode.

Time (min)	Mobile phase A ACN/0.1% formic acid	Mobile phase B H_2O/0.1% formic acid
0.000	5%	95%
4.000	5%	95%
19.000	70%	30%
21.000	70%	30%
22.000	5%	95%
30.000	5%	95%

For negative ion mode analysis, a gradient-type HPLC method was used during each run with 50 mM ammonium acetate as mobile phase A and acetonitrile as mobile phase B. An injection volume of 20 µL of the extract was introduced onto a Hypersil GOLD™ C18 column (250 mm × 4.6 mm, particle size 5 µm, Thermo Scientific, United States) held at room temperature, with a solvent flow rate of 0.8 mL min^{-1} to drive the chromatographic separation. The detection wavelength was set to 260 nm.

Table S2: Flow Gradient for Loading Pump in HPLC Assay in negative analysis mode.

Time (min)	Mobile phase A 50 mM ammonium acetate	Mobile phase B acetonitrile
0.000	100%	0%
1.000	100%	0%
21.000	50%	50%
26.000	50%	50%
30.000	100%	0%

Mass measurements were carried out in positive or negative ESI mode, with source gases set at sheath gas = 35 a.u, aux gas = 15 a.u., sweep gas = 0 arbitrary units. The ESI spray voltage was set to 3800 V, and the capillary temperature was set at 320 °C. Mass spectra were registered in the scan range of 50-750 m/z or 150-2000 m/z.

Mass spectrometry data analysis was performed using Thermo Scientific™ Xcalibur™ 4.7 software.

Supplementary Figures and Tables

CAP promotes peptide synthesis with metal ions

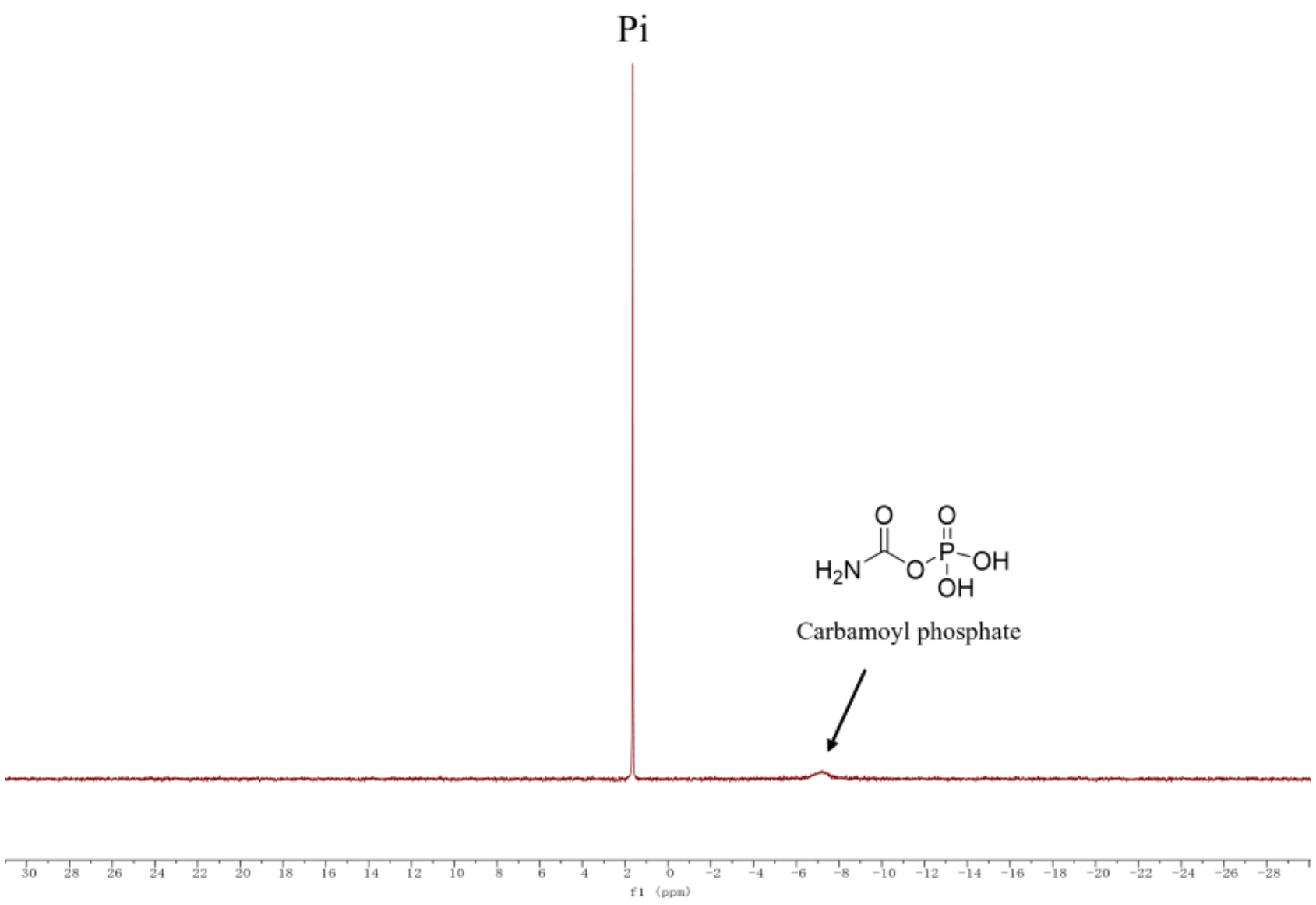

Fig. S1 The NMR spectra clearly show that the characteristic peaks of CAP are still detectable even after 5 days of reaction.

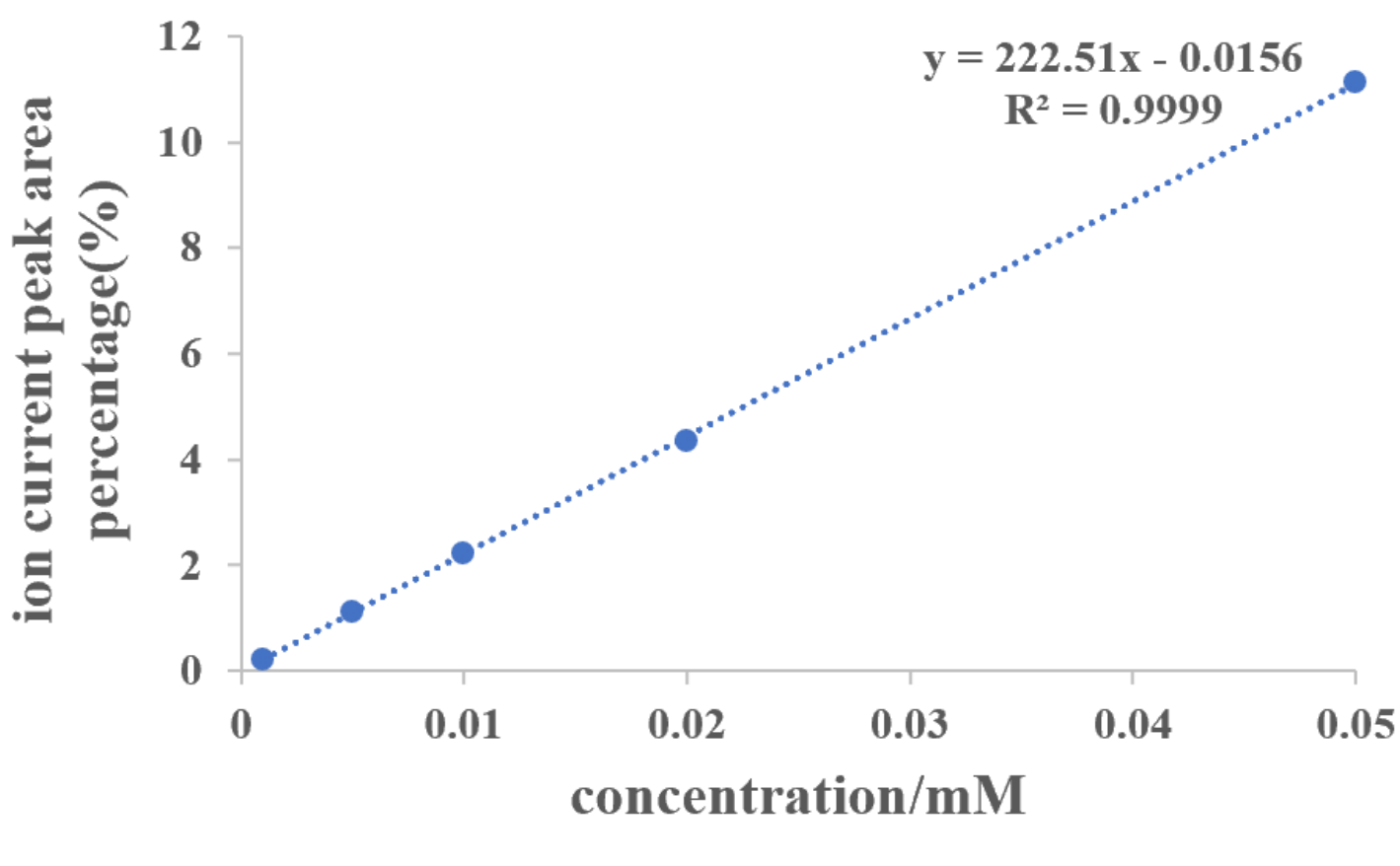

Fig. S2 The standard curve for detecting Ala_2 in samples by liquid chromatography-mass spectrometry. The X-axis represents the concentration of the standard sample of Ala_2, and the Y-axis represents the chromatographic peak area. The curve was linearly fitted using the weighted least squares method ($1/x^2$), and the fitting equation was $y = 222.51x + 0.3183$, with $R^2 = 0.9999$.

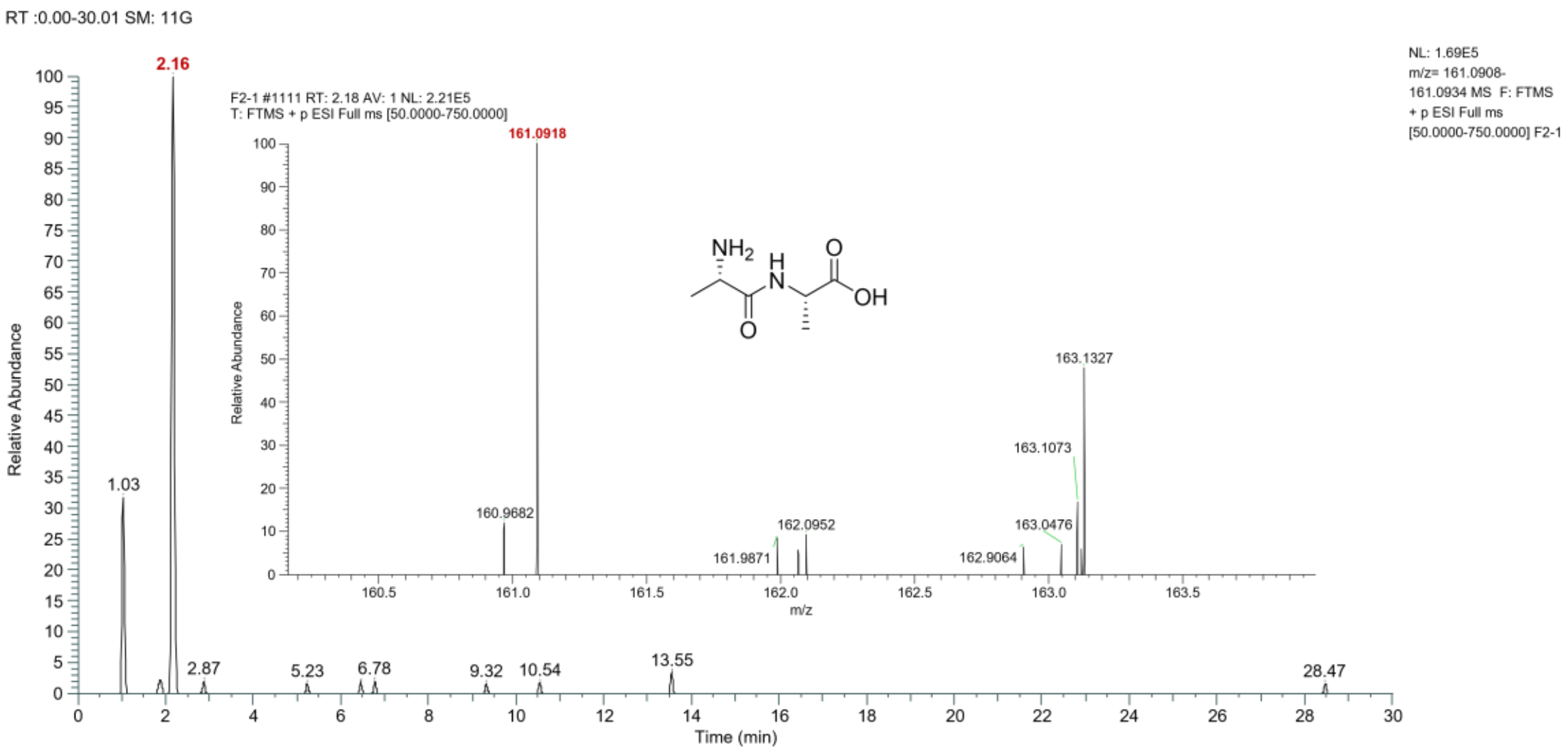

Fig. S3 The extraction ion chromatography-mass spectrometry (EIC-MS) data of Ala_2. The analysis results confirm that Ala_2 was formed in the reaction system containing alanine, CAP, and Fe^{2+}. The extraction ion plot (EIC) shows a peak at m/z 161.0918, corresponding to Ala_2, with a retention time of 2.16 minutes.

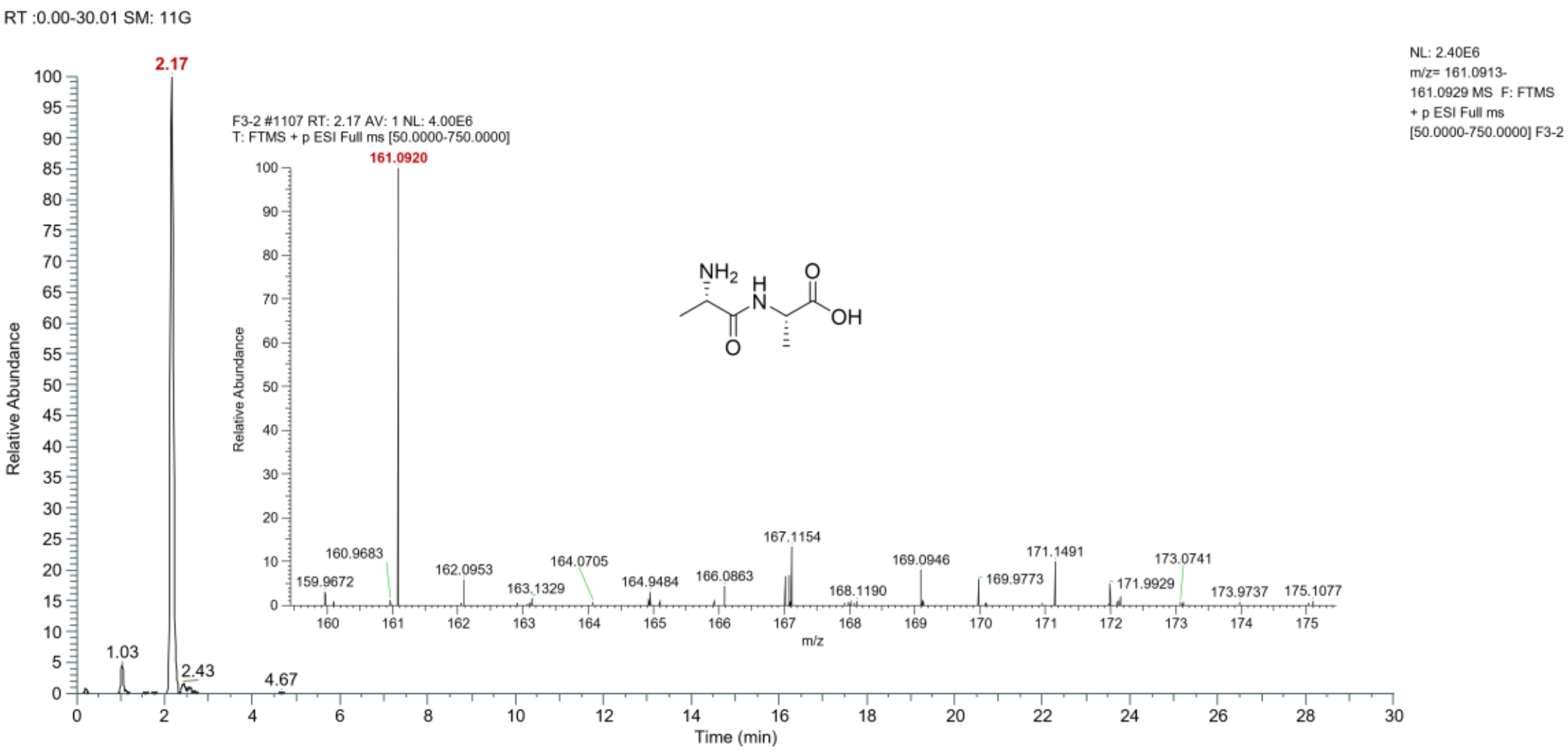

Fig. S4 The extraction ion chromatography-mass spectrometry (EIC-MS) data of Ala_2. The analysis results confirm that Ala_2 was formed in the reaction system containing alanine, CAP, and Fe^{3+}. The extraction ion plot (EIC) shows a peak at m/z 161.0920, corresponding to Ala_2, with a retention time of 2.17 minutes.

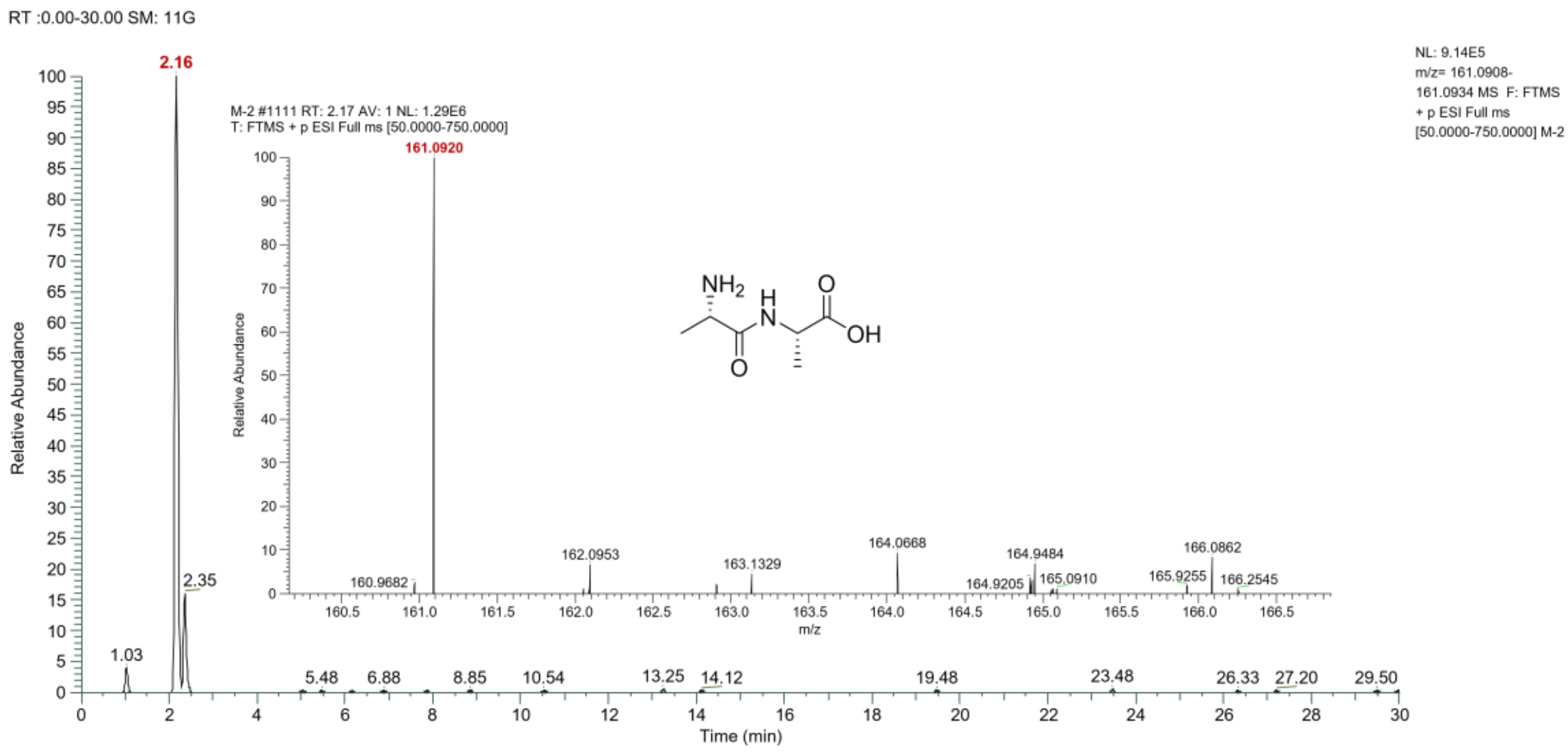

Fig. S5 The extraction ion chromatography-mass spectrometry (EIC-MS) data of Ala$_2$. The analysis results confirm that Ala$_2$ was formed in the reaction system containing alanine, CAP, and Mg^{2+}. The extraction ion plot (EIC) shows a peak at m/z 161.0920, corresponding to Ala$_2$, with a retention time of 2.16 minutes.

Ba^{2+}, Mn^{2+}, and Cu^{2+} can be combined in pairs to promote peptide formation.

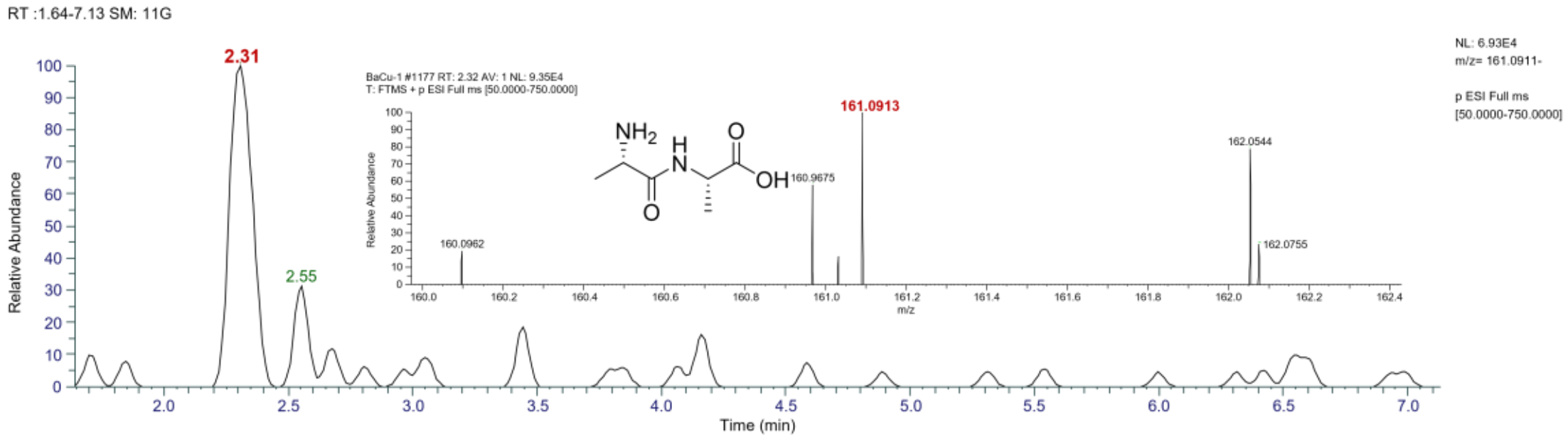

Fig. S6 The extraction ion chromatography-mass spectrometry (EIC-MS) data of Ala_2. The analysis results confirm that Ala_2 was formed in the reaction system containing Ala, CAP, Cu^{2+} and Ba^{2+}. The extraction ion plot (EIC) shows a peak at m/z 161.0914, corresponding to Ala_2, with a retention time of 2.31 minutes.

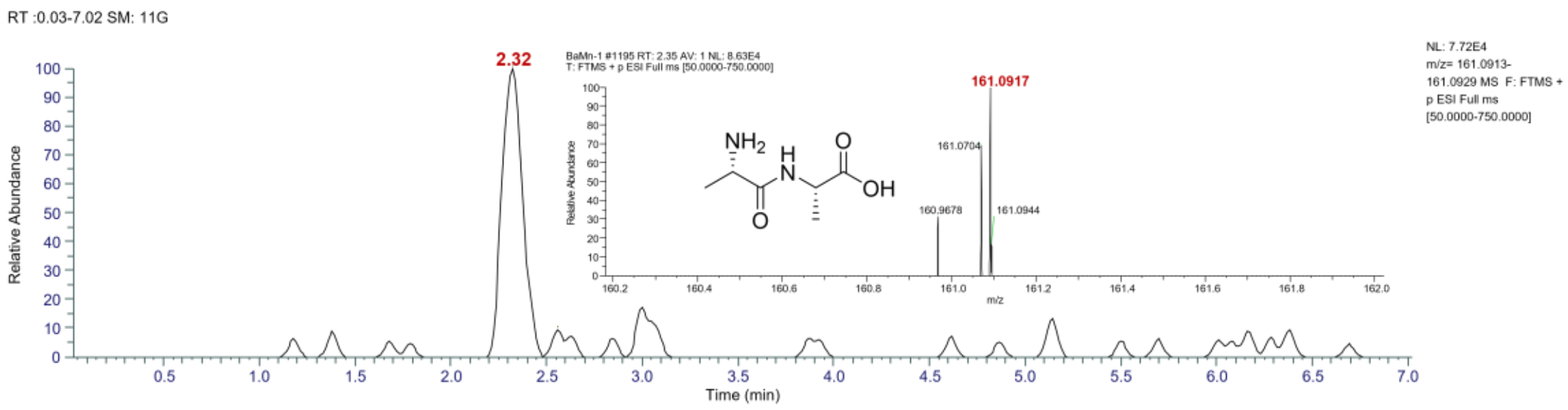

Fig. S7 The extraction ion chromatography-mass spectrometry (EIC-MS) data of Ala_2. The analysis results confirm that Ala_2 was formed in the reaction system containing Ala, CAP, Mn^{2+} and Ba^{2+}. The extraction ion plot (EIC) shows a peak at m/z 161.0914, corresponding to Ala_2, with a retention time of 2.32 minutes.

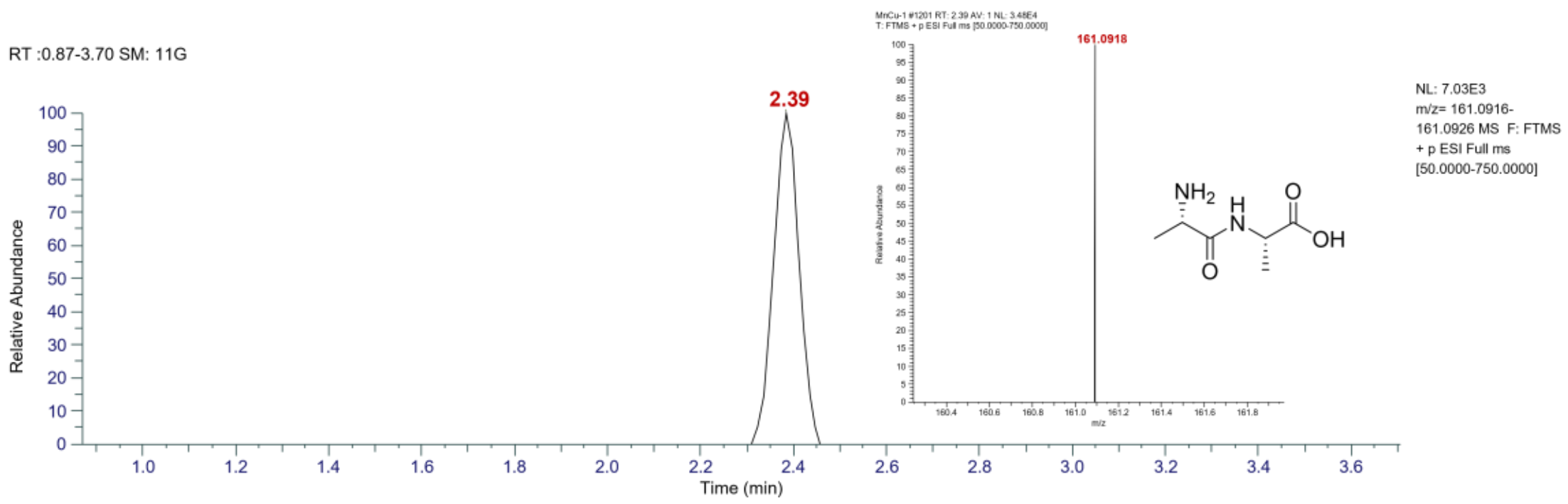

Fig. S8 The extraction ion chromatography-mass spectrometry (EIC-MS) data of Ala_2. The analysis results confirm that Ala_2 was formed in the reaction system containing Ala, CAP, Cu^{2+} and Mn^{2+}. The extraction ion plot (EIC) shows a peak at m/z 161.0914, corresponding to Ala_2, with a retention time of 2.39 minutes.

Optimization of pH and Metal Ions

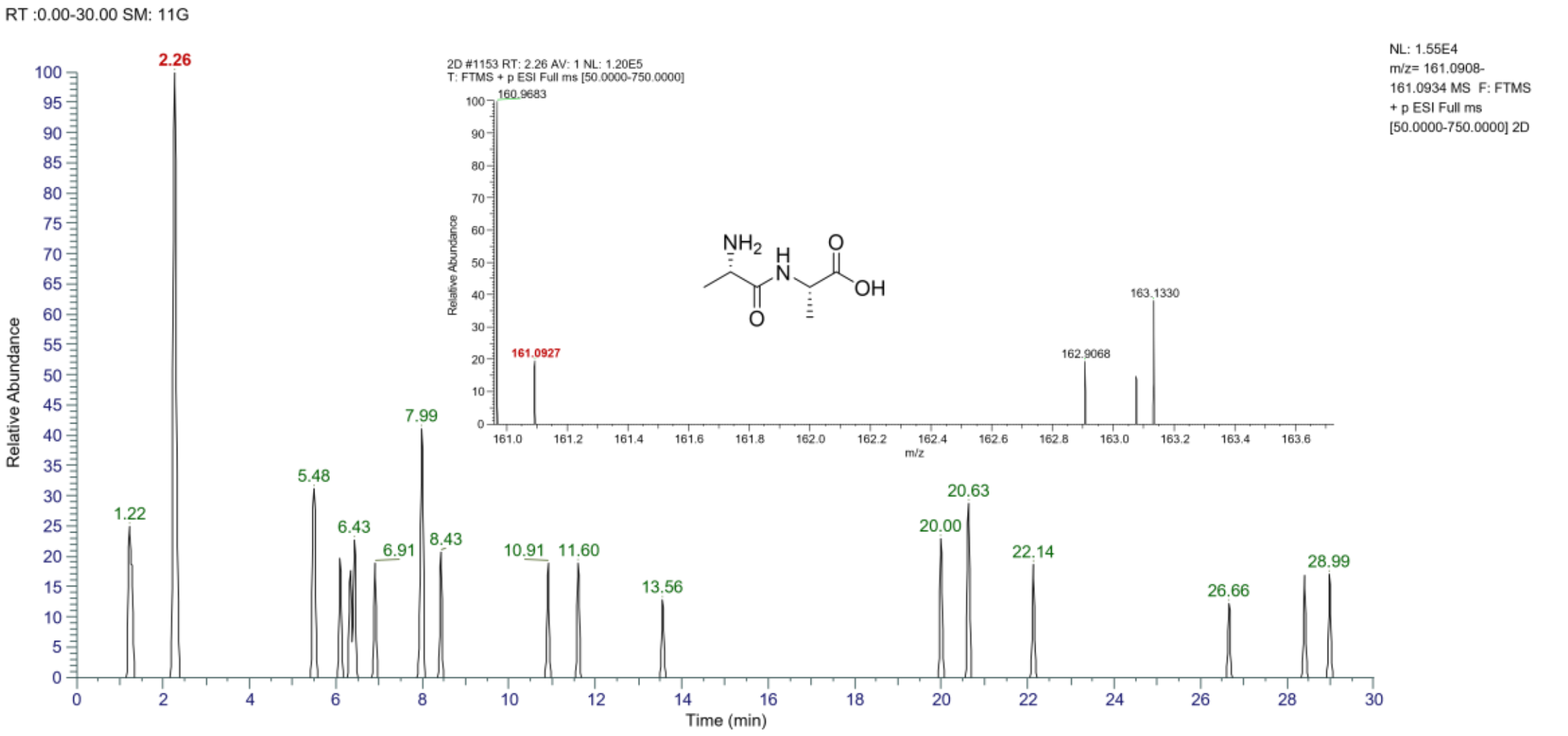

Fig. S9 The extraction ion chromatography-mass spectrometry (EIC-MS) data of Ala_2 (pH 3). The analysis results confirm that Ala_2 was formed in the reaction system containing Ala, CAP, and Mg^{2+}. The extraction ion chromatogram (EIC) shows a peak at m/z 161.0927, corresponding to Ala_2, with a retention time of 2.26 minutes.

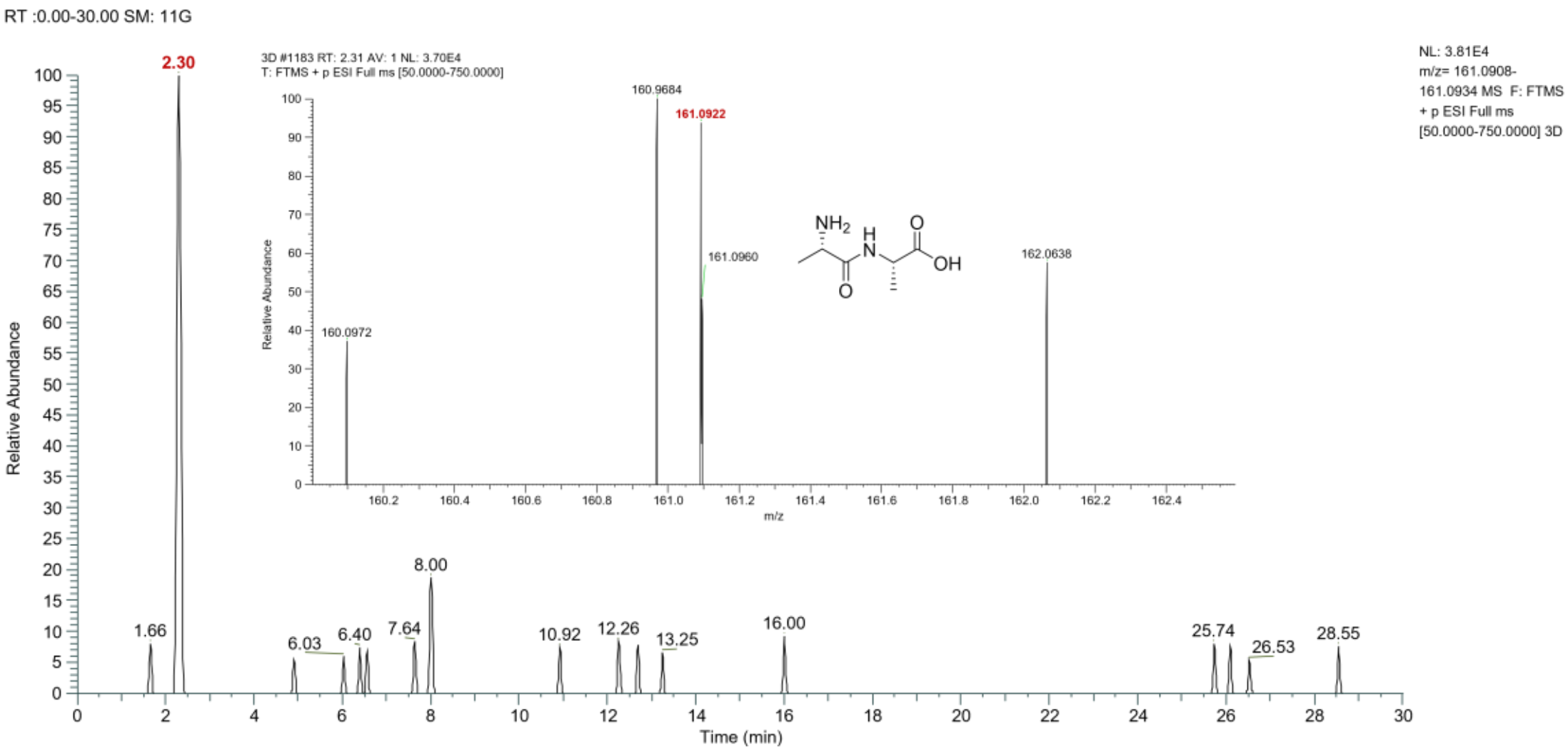

Fig. S10 The extraction ion chromatography-mass spectrometry (EIC-MS) data of Ala_2 (pH 4). The analysis results confirm that Ala_2 was formed in the reaction system containing Ala, CAP, and M Mg^{2+}. The extraction ion chromatogram (EIC) shows a peak at m/z 161.0922, corresponding to Ala_2, with a retention time of 2.30 minutes.

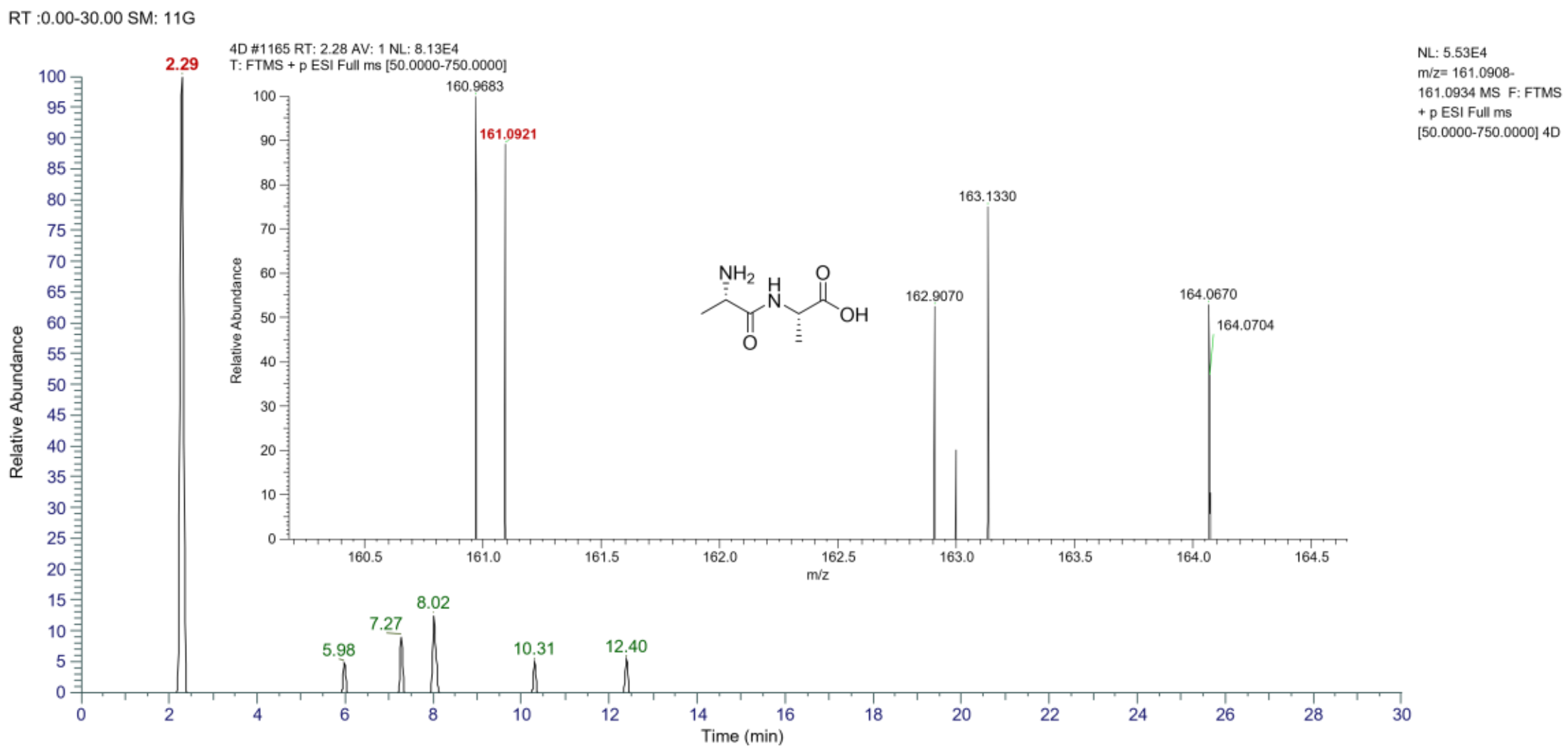

Fig. S11 The extraction ion chromatography-mass spectrometry (EIC-MS) data of Ala_2 (pH 5). The analysis results confirm that Ala_2 was formed in the reaction system containing Ala, CAP, and Mg^{2+}. The extraction ion chromatogram (EIC) shows a peak at m/z 161.0921, corresponding to Ala_2, with a retention time of 2.29 minutes.

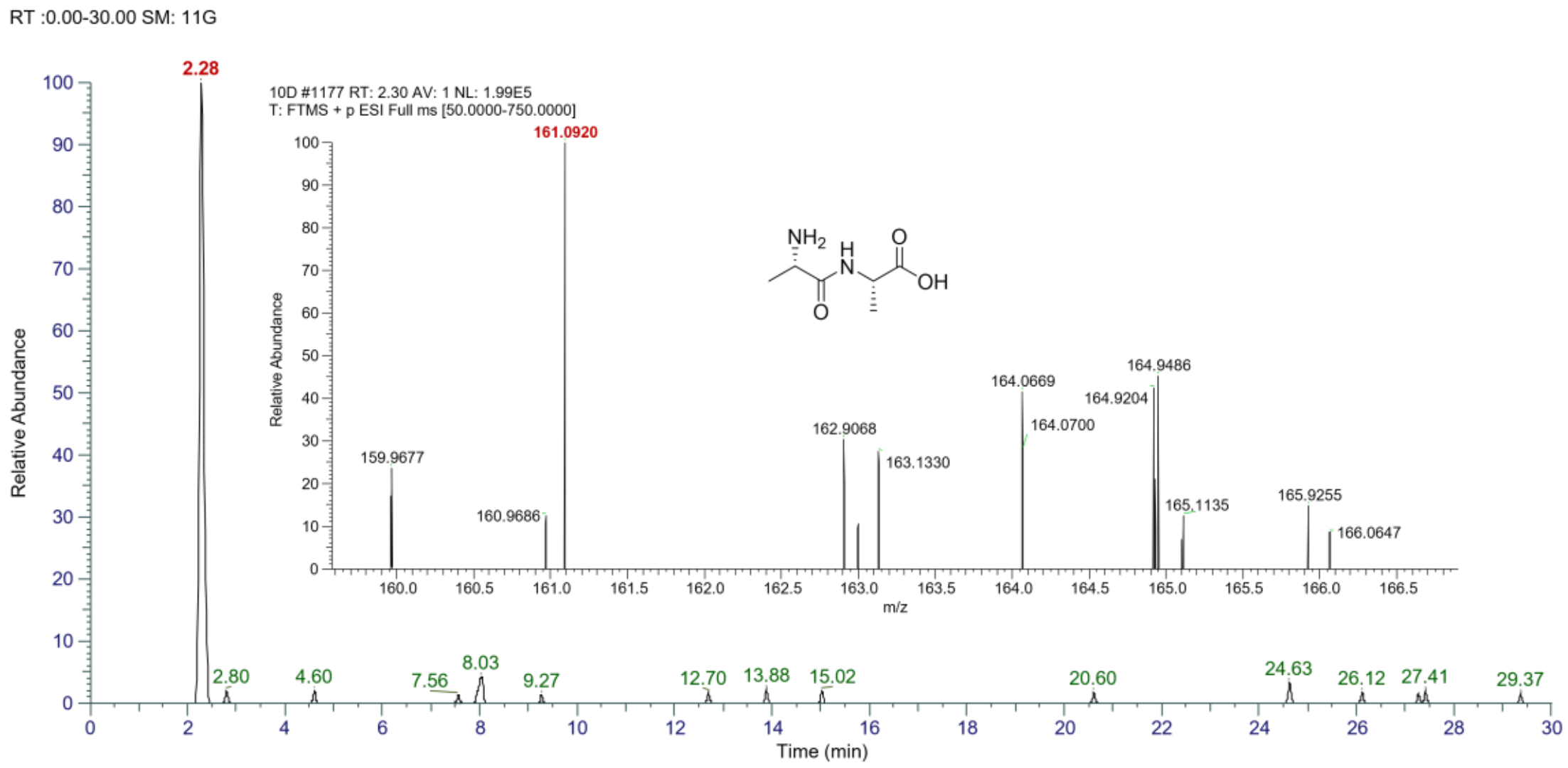

Fig. S12 The extraction ion chromatography-mass spectrometry (EIC-MS) data of Ala_2 (pH 6). The analysis results confirm that Ala_2 was formed in the reaction system containing Ala, CAP, and 1.0 M Mg^{2+}. The extraction ion chromatogram (EIC) shows a peak at m/z 161.0923, corresponding to Ala_2, with a retention time of 2.28 minutes.

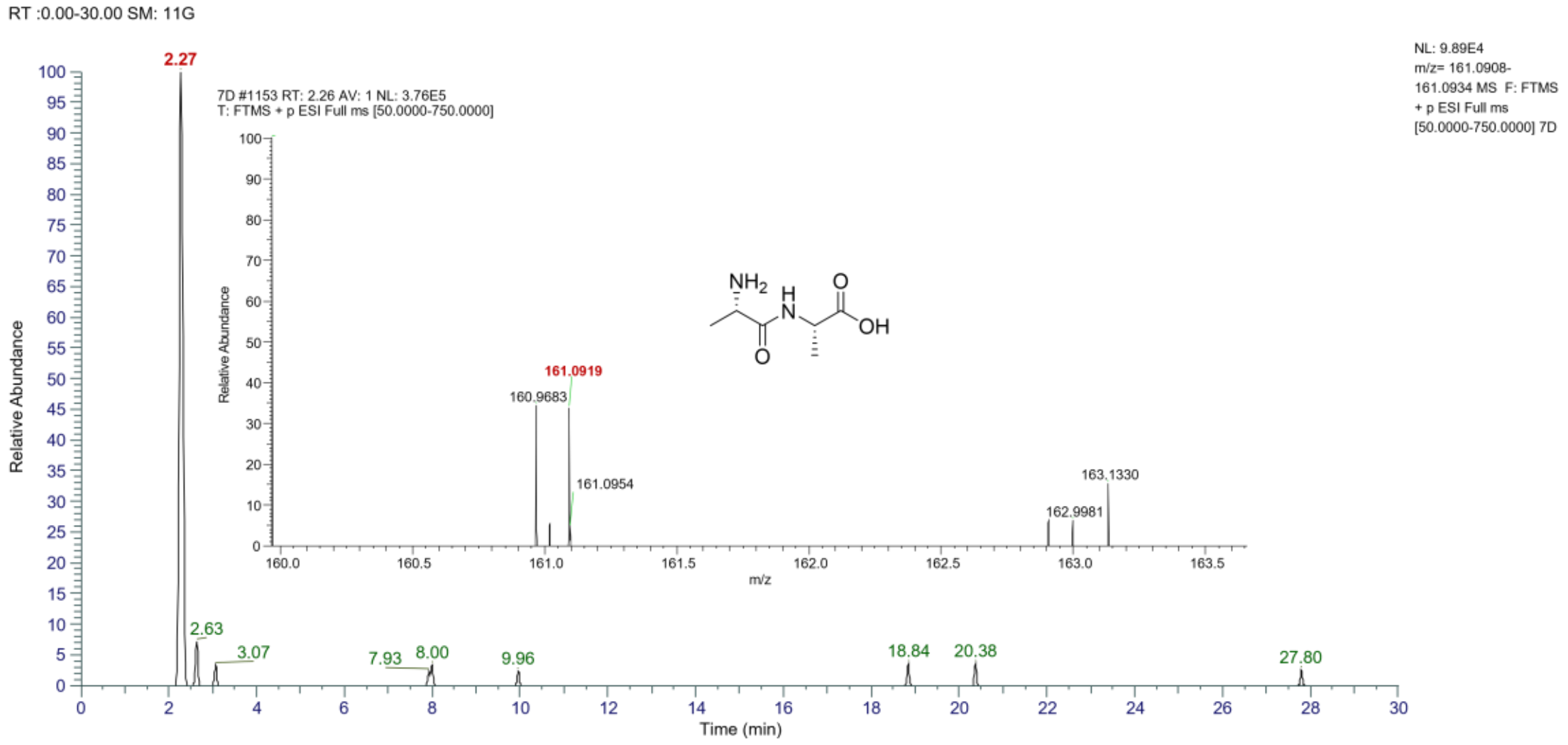

Fig. S13 The extraction ion chromatography-mass spectrometry (EIC-MS) data of Ala_2 (pH 7). The analysis results confirm that Ala_2 was formed in the reaction system containing Ala, CAP, and Mg^{2+}. The extraction ion chromatogram (EIC) shows a peak at m/z 161.0919, corresponding to Ala_2, with a retention time of 2.27 minutes.

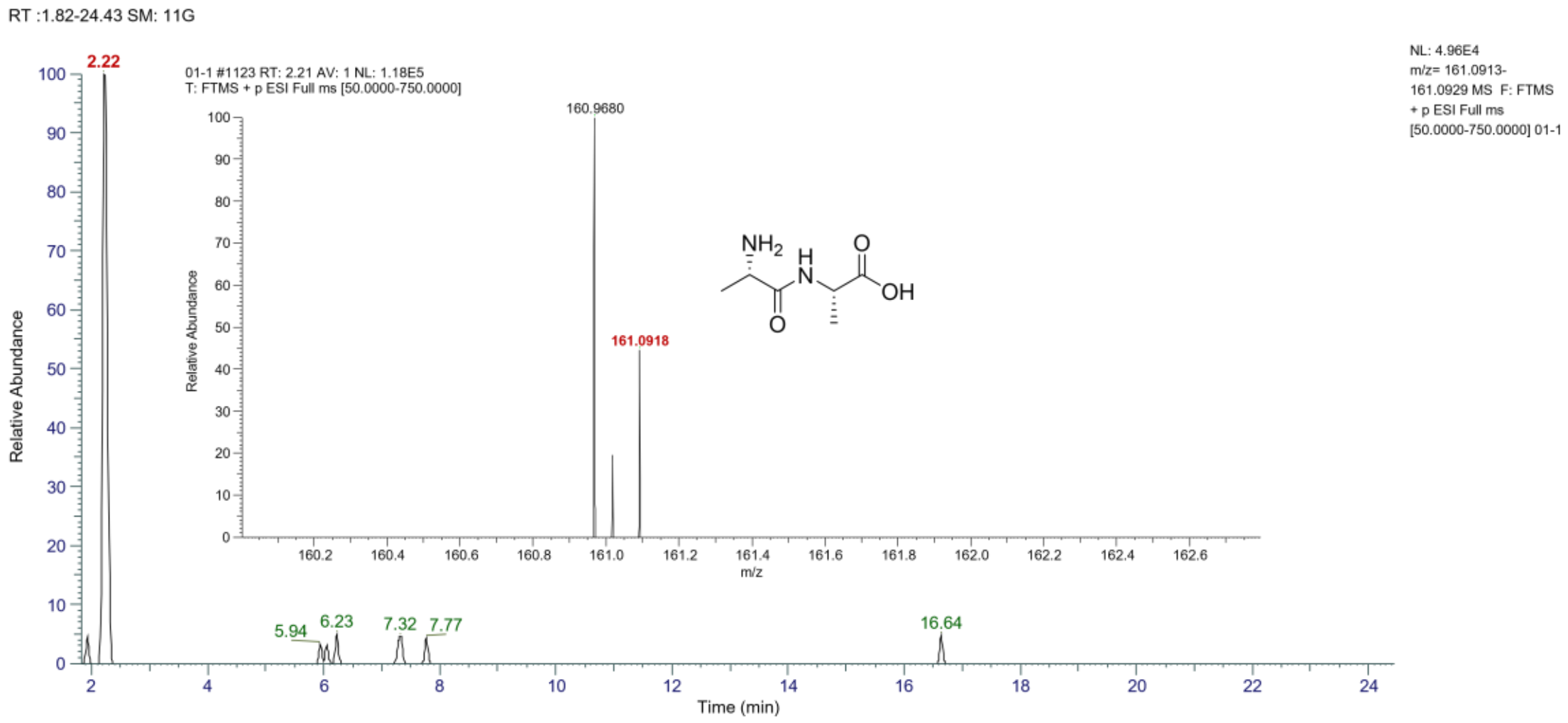

Fig. S14 The extraction ion chromatography-mass spectrometry (EIC-MS) data of Ala_2. The analysis results confirm that Ala_2 was formed in the reaction system containing Ala, CAP, and 0.1 M Mg^{2+}. The extraction ion chromatogram (EIC) shows a peak at m/z 161.0918, corresponding to Ala_2, with a retention time of 2.22 minutes.

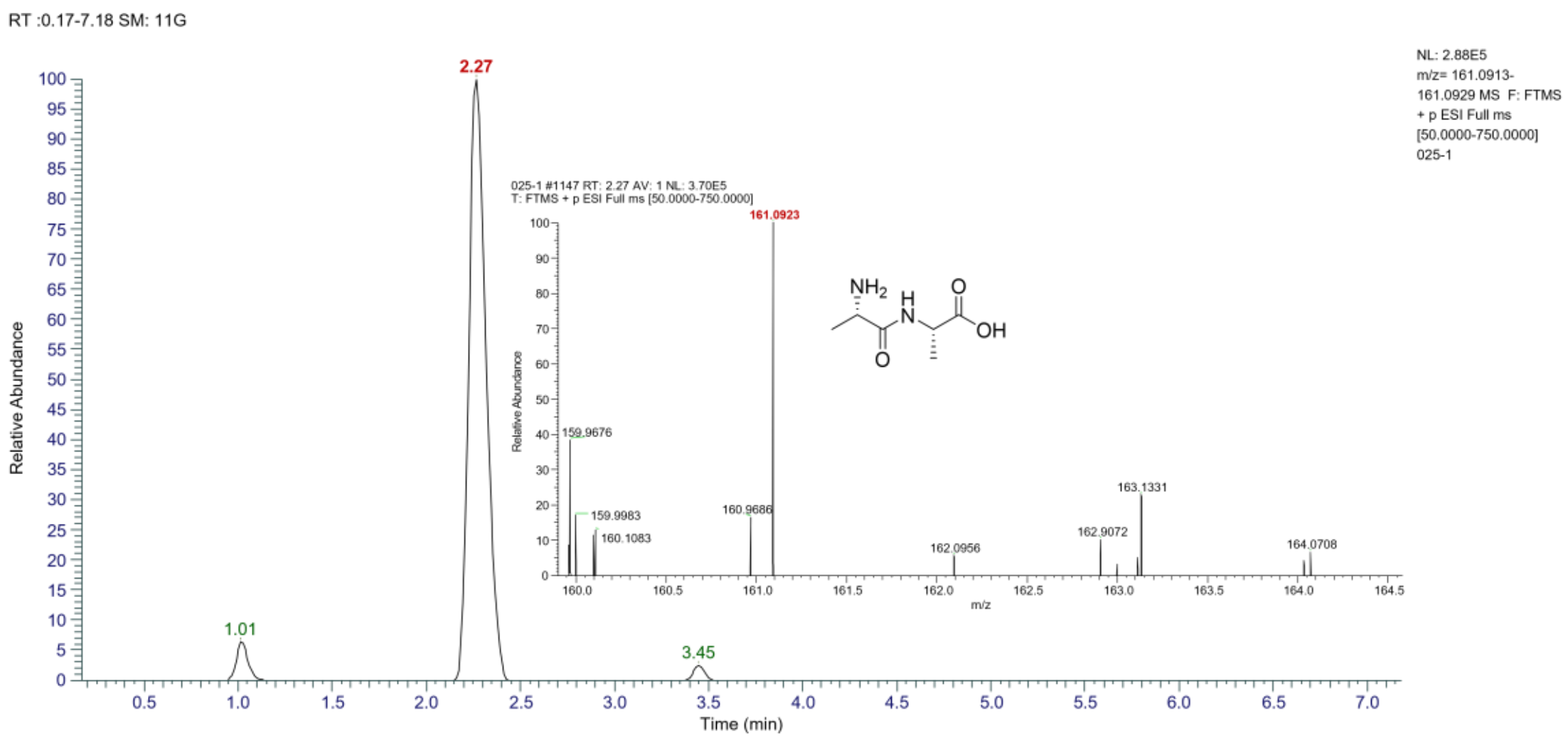

Fig. S15 The extraction ion chromatography-mass spectrometry (EIC-MS) data of Ala_2. The analysis results confirm that Ala_2 was formed in the reaction system containing Ala, CAP, and 0.25 M Mg^{2+}. The extraction ion chromatogram (EIC) shows a peak at m/z 161.0918, corresponding to Ala_2, with a retention time of 2.27 minutes.

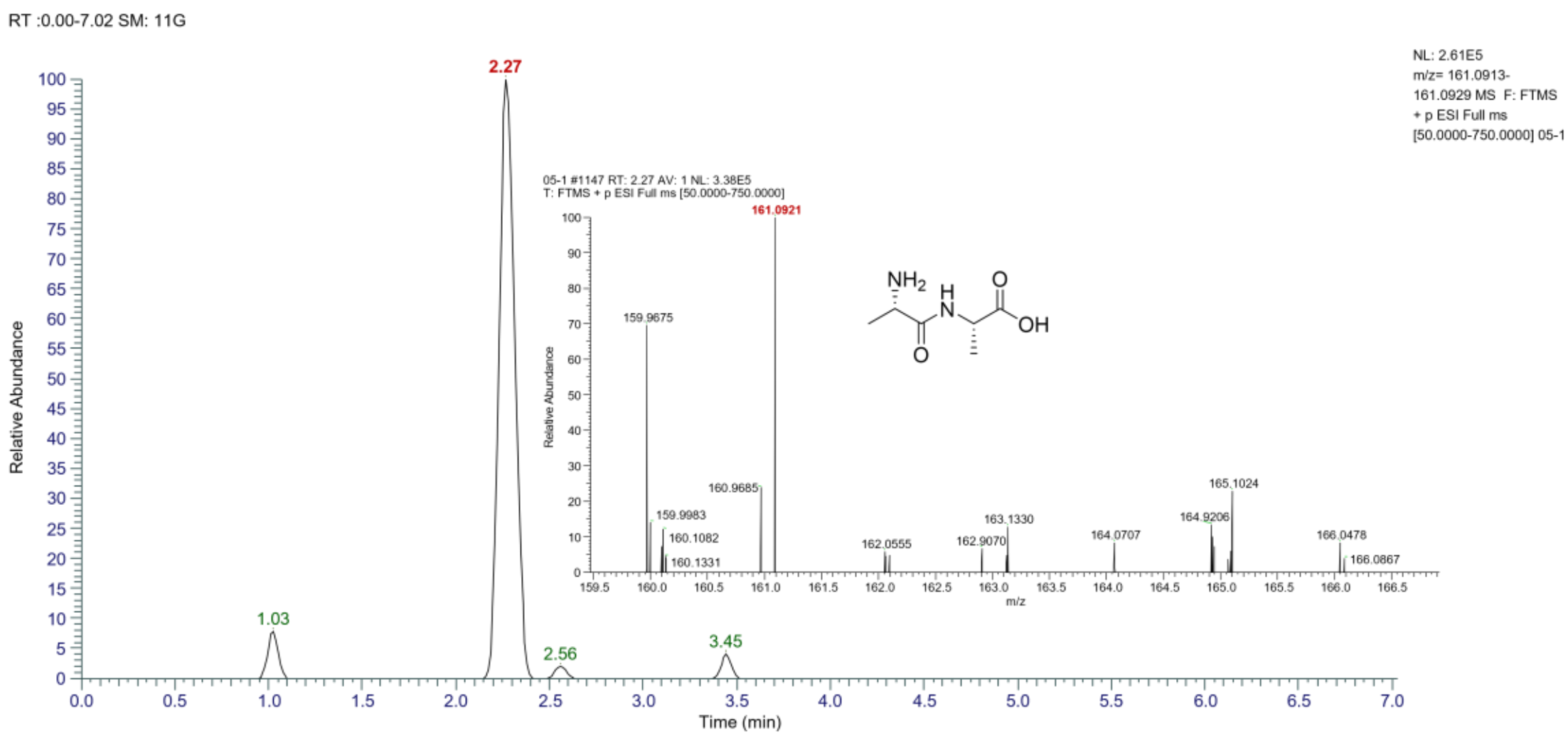

Fig. S16 The extraction ion chromatography-mass spectrometry (EIC-MS) data of Ala_2. The analysis results confirm that Ala_2 was formed in the reaction system containing Ala, CAP, and 0.5 M Mg^{2+}. The extraction ion chromatogram (EIC) shows a peak at m/z 161.0918, corresponding to Ala_2, with a retention time of 2.27 minutes.

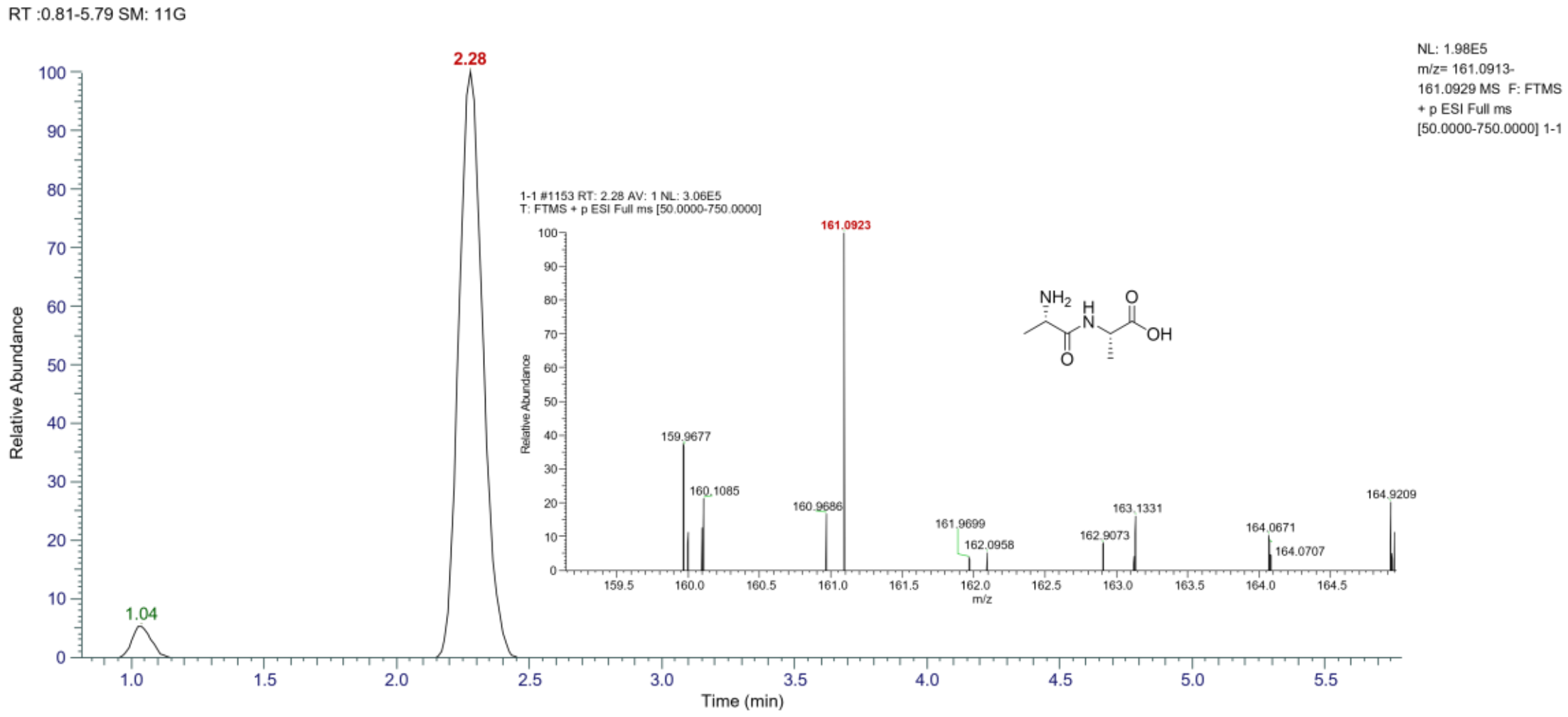

Fig. S17 The extraction ion chromatography-mass spectrometry (EIC-MS) data of Ala$_2$. The analysis results confirm that Ala$_2$ was formed in the reaction system containing Ala, CAP, and 1.0 M Mg^{2+}. The extraction ion chromatogram (EIC) shows a peak at m/z 161.0923, corresponding to Ala$_2$, with a retention time of 2.28 minutes.

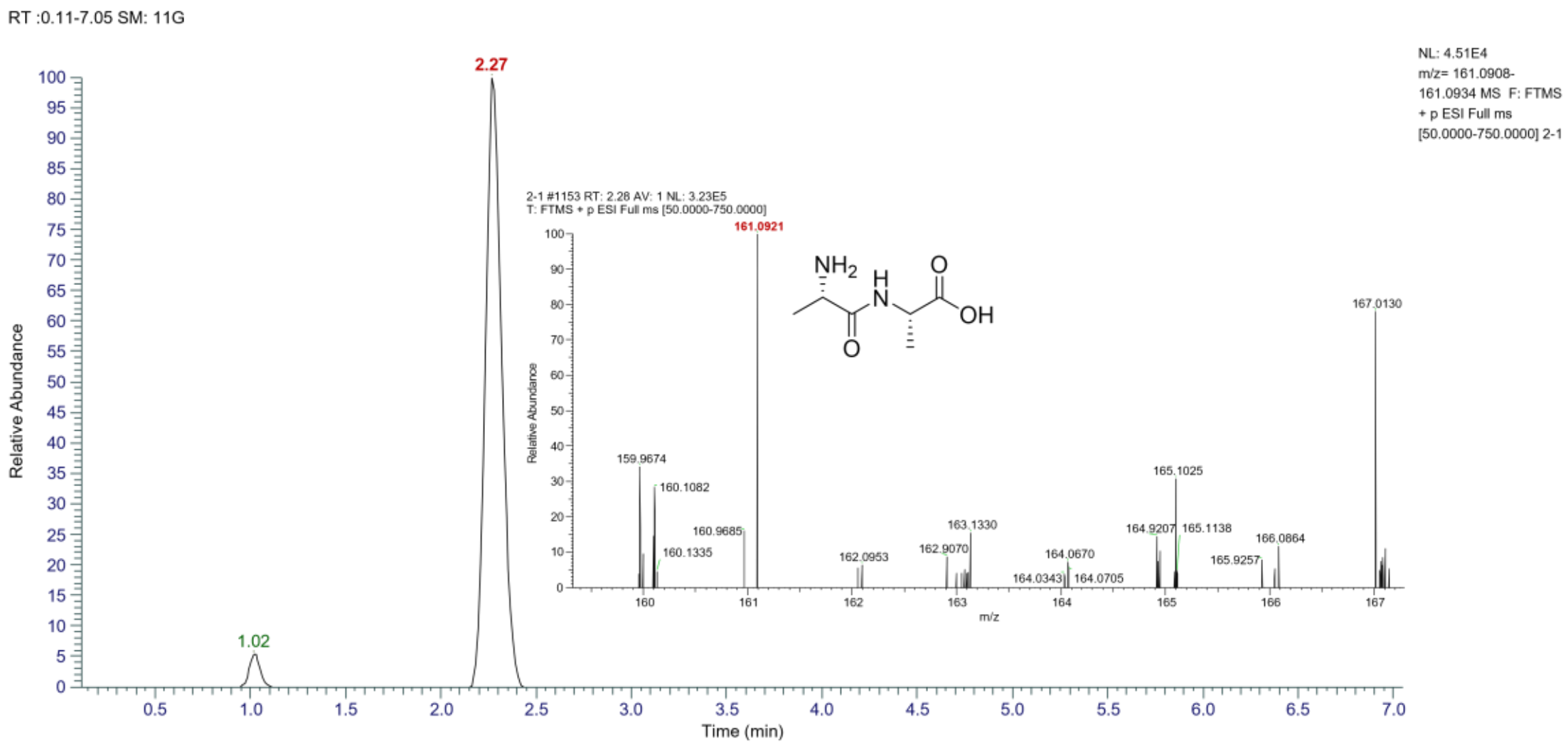

Fig. S18 The extraction ion chromatography-mass spectrometry (EIC-MS) data of Ala$_2$. The analysis results confirm that Ala$_2$ was formed in the reaction system containing Ala, CAP, and 2.0 M Mg^{2+}. The extraction ion chromatogram (EIC) shows a peak at m/z 161.0921, corresponding to Ala$_2$, with a retention time of 2.27 minutes.

CAP mediated amino acid peptide formation

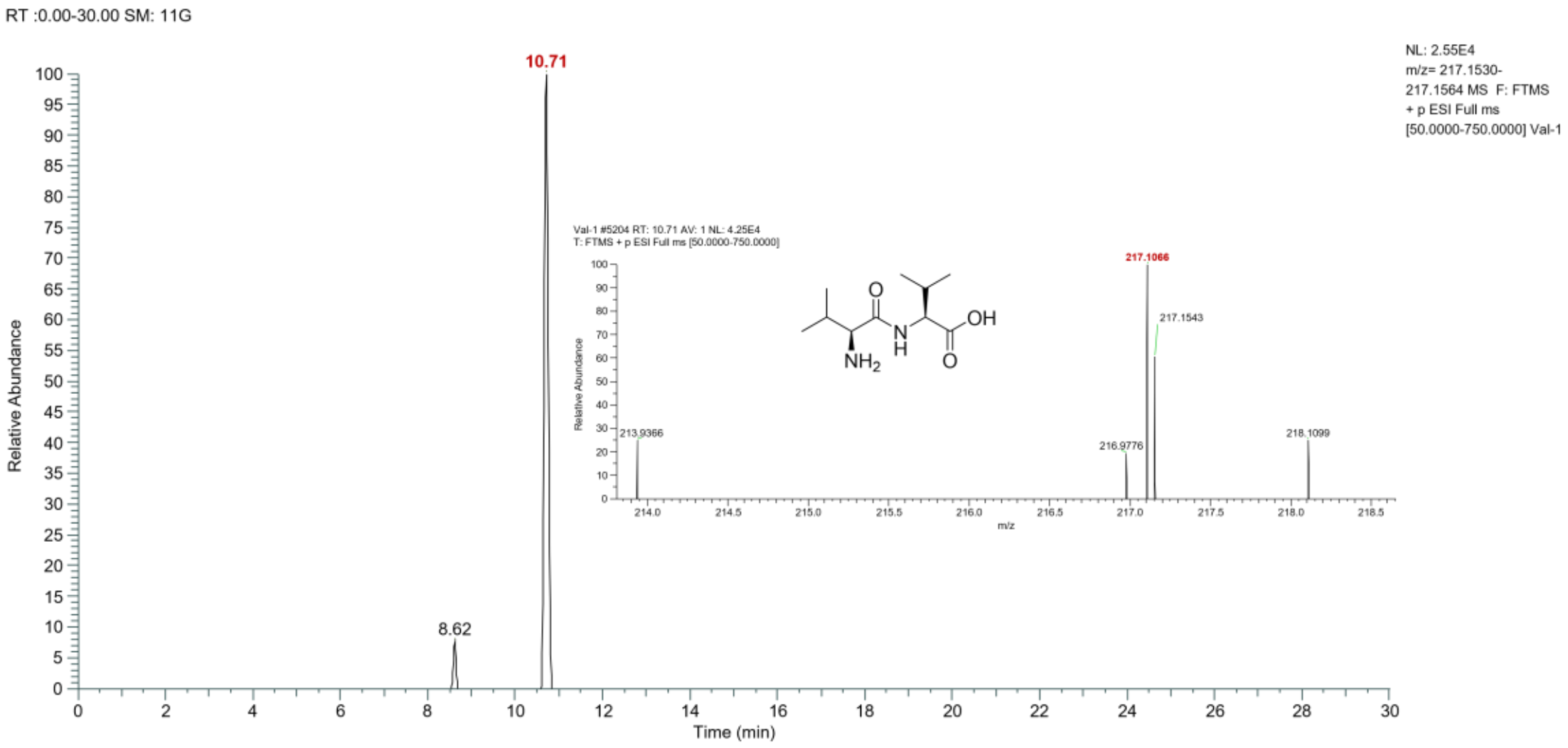

Fig. S19 The extraction ion chromatography-mass spectrometry (EIC-MS) data of Val_2. The analysis results confirm that Val_2 was formed in the reaction system containing Val, CAP, and 0.25 M Mg^{2+}. The extraction ion chromatogram (EIC) shows a peak at m/z 217.1066, corresponding to Val_2, with a retention time of 10.71 minutes.

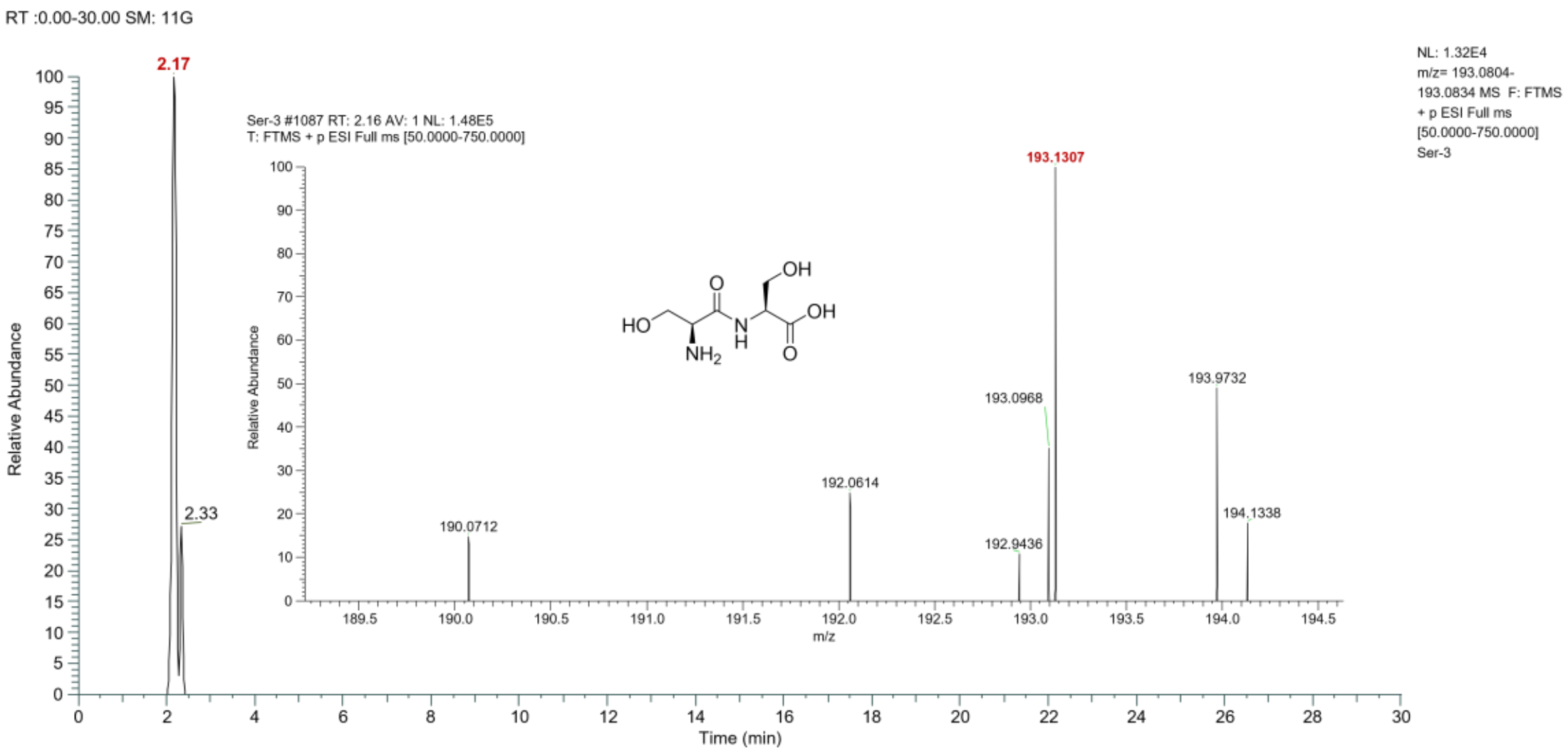

Fig. S20 The extraction ion chromatography-mass spectrometry (EIC-MS) data of Ser_2. The analysis results confirm that Ser_2 was formed in the reaction system containing Ser, CAP, and 0.25 M Mg^{2+}. The extraction ion chromatogram (EIC) shows a peak at m/z 193.1307, corresponding to Ser_2, with a retention time of 2.17 minutes.

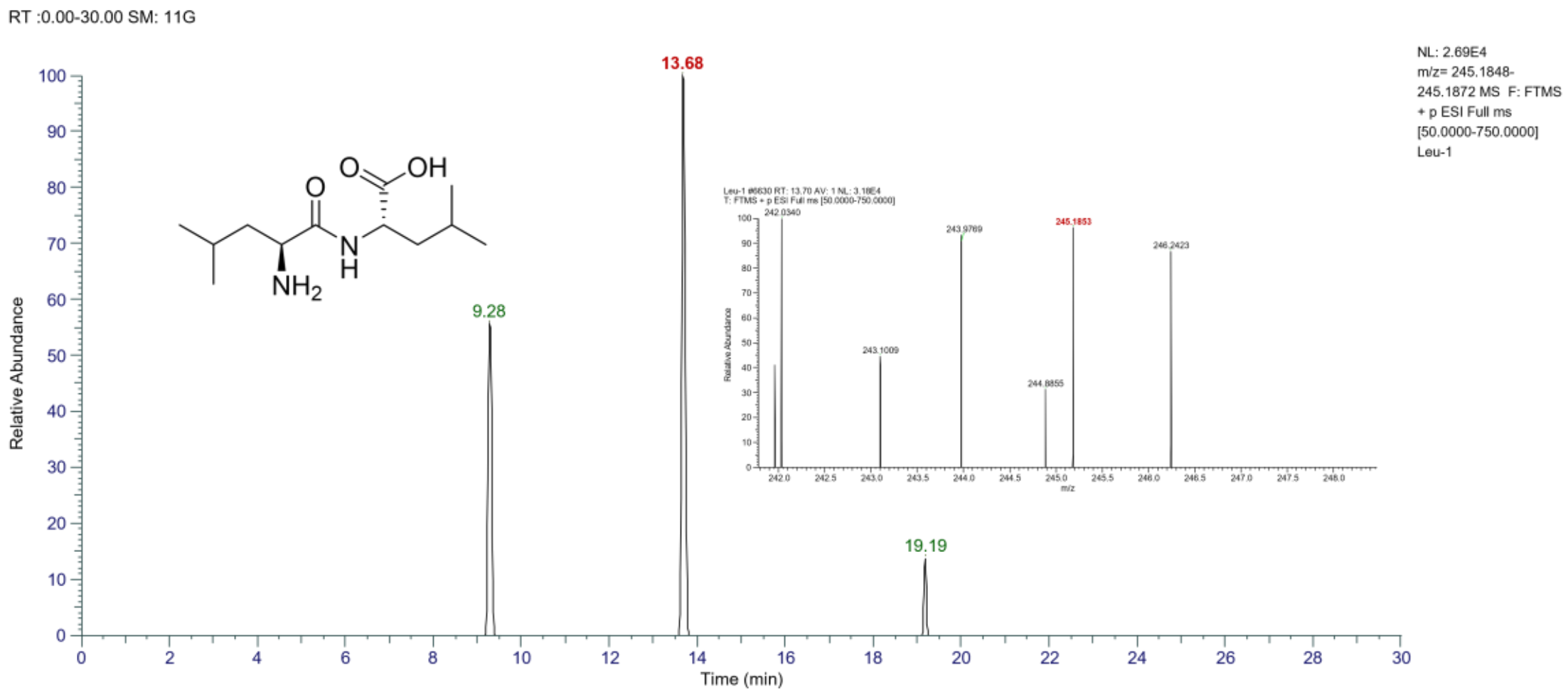

Fig. S21 The extraction ion chromatography-mass spectrometry (EIC-MS) data of Leu_2. The analysis results confirm that Leu_2 was formed in the reaction system containing Leu, CAP, and 0.25 M Mg^{2+}. The extraction ion chromatogram (EIC) shows a peak at m/z 245.1853, corresponding to Leu_2, with a retention time of 13.68 minutes.

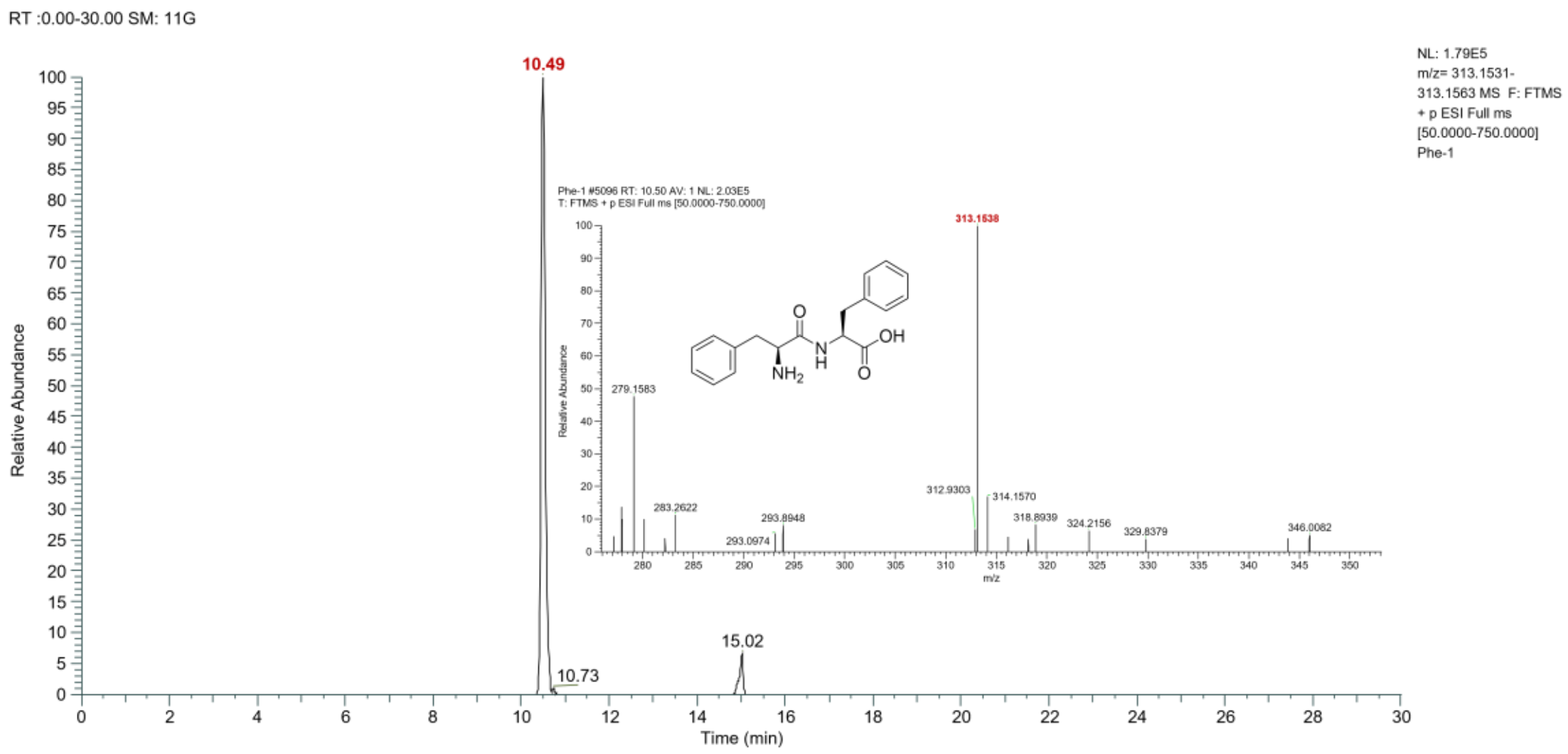

Fig. S22 The extraction ion chromatography-mass spectrometry (EIC-MS) data of Phe_2. The analysis results confirm that Phe_2 was formed in the reaction system containing Phe, CAP, and 0.25 M Mg^{2+}. The extraction ion chromatogram (EIC) shows a peak at m/z 313.1538, corresponding to Phe_2, with a retention time of 10.49 minutes.

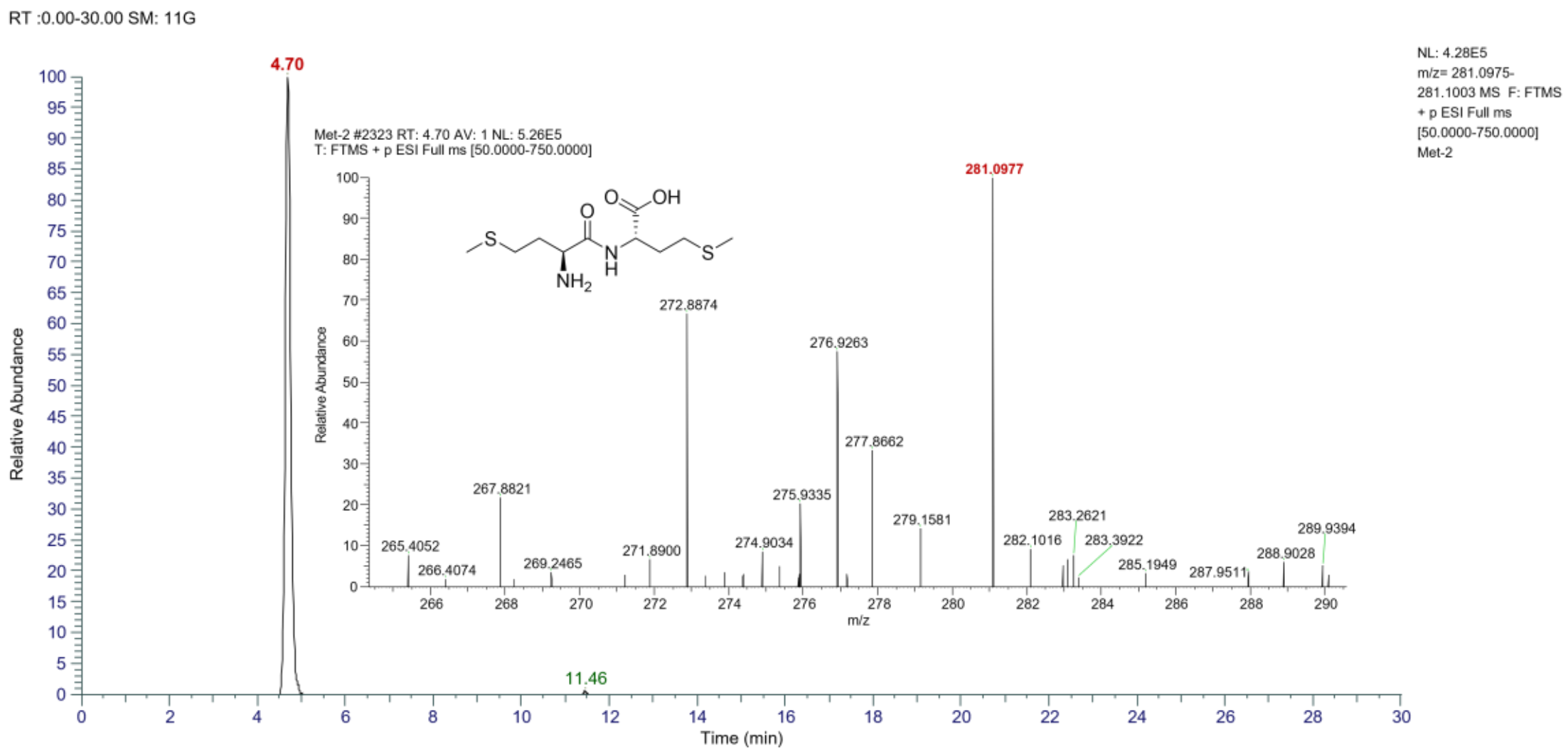

Fig. S23 The extraction ion chromatography-mass spectrometry (EIC-MS) data of Met_2. The analysis results confirm that Met_2 was formed in the reaction system containing Met, CAP, and 0.25 M Mg^{2+}. The extraction ion chromatogram (EIC) shows a peak at m/z 281.0977, corresponding to Met_2, with a retention time of 4.70 minutes.

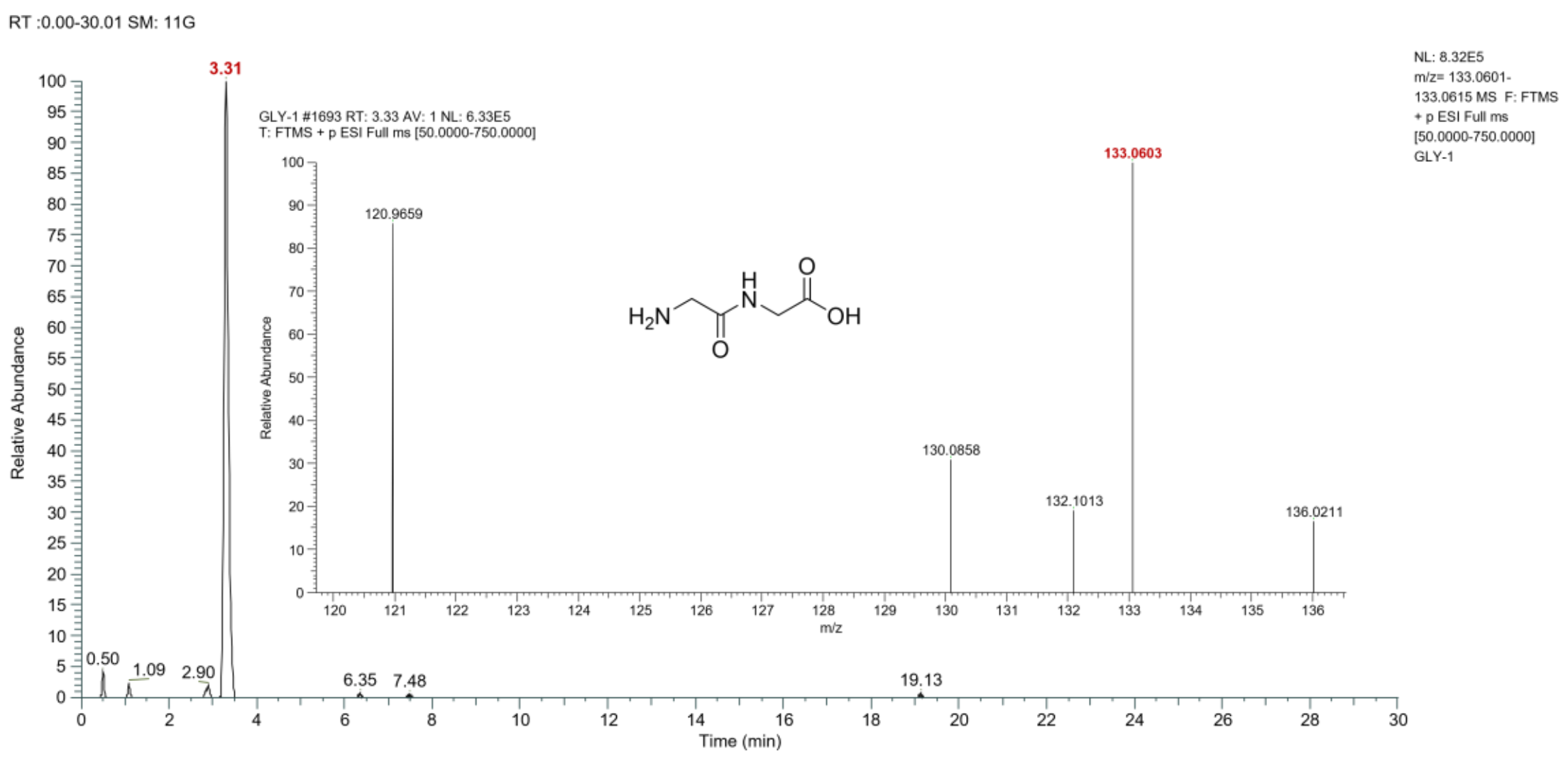

Fig. S24 The extraction ion chromatography-mass spectrometry (EIC-MS) data of Gly_2. The analysis results confirm that Gly_2 was formed in the reaction system containing Gly, CAP, and 0.25 M Mg^{2+}. The extraction ion chromatogram (EIC) shows a peak at m/z 133.0603, corresponding to Gly_2, with a retention time of 3.31 minutes.

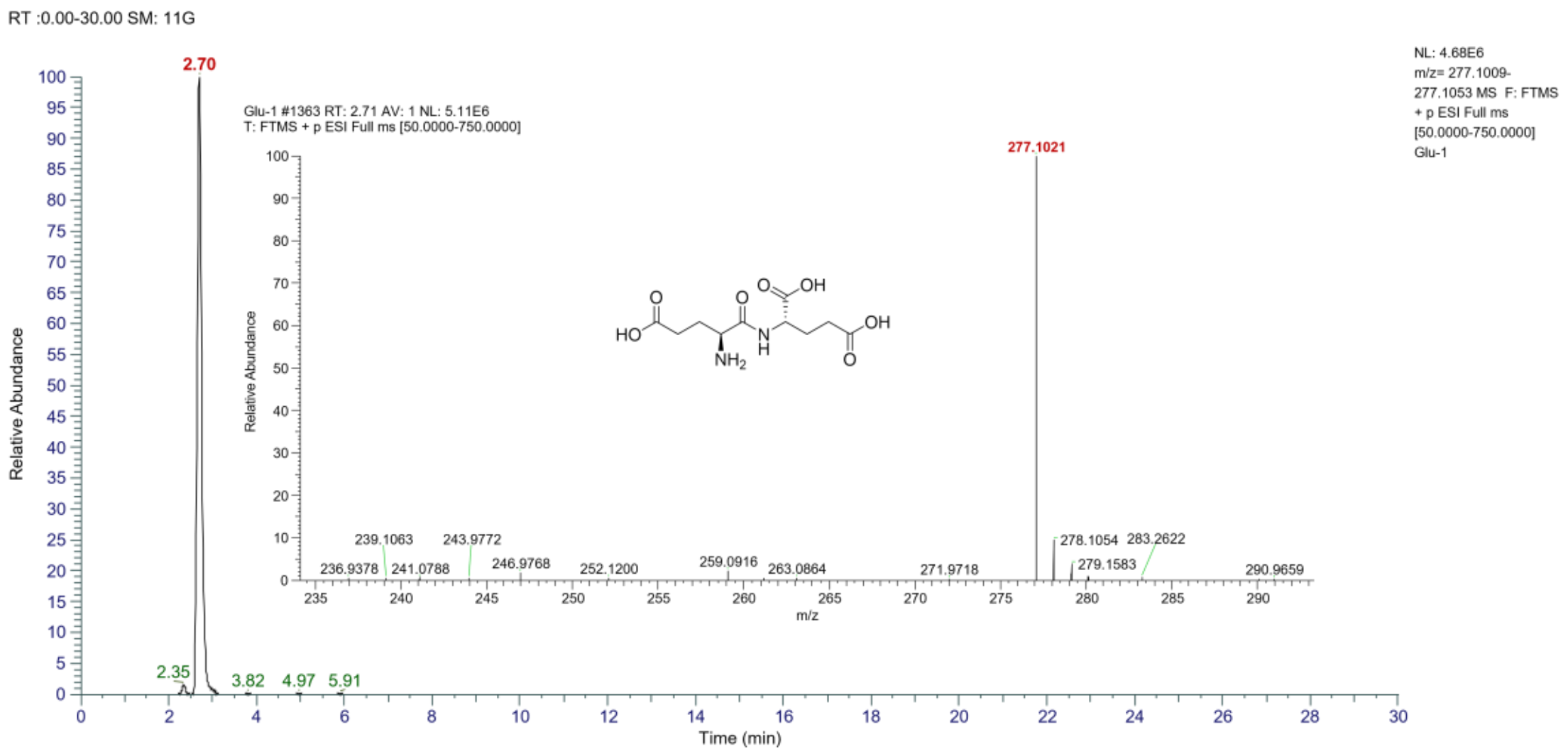

Fig. S25 The extraction ion chromatography-mass spectrometry (EIC-MS) data of Glu₂. The analysis results confirm that Glu_2 was formed in the reaction system containing Glu, CAP, and 0.25 M Mg^{2+}. The extraction ion chromatogram (EIC) shows a peak at m/z 277.1021, corresponding to Glu_2, with a retention time of 2.70 minutes.

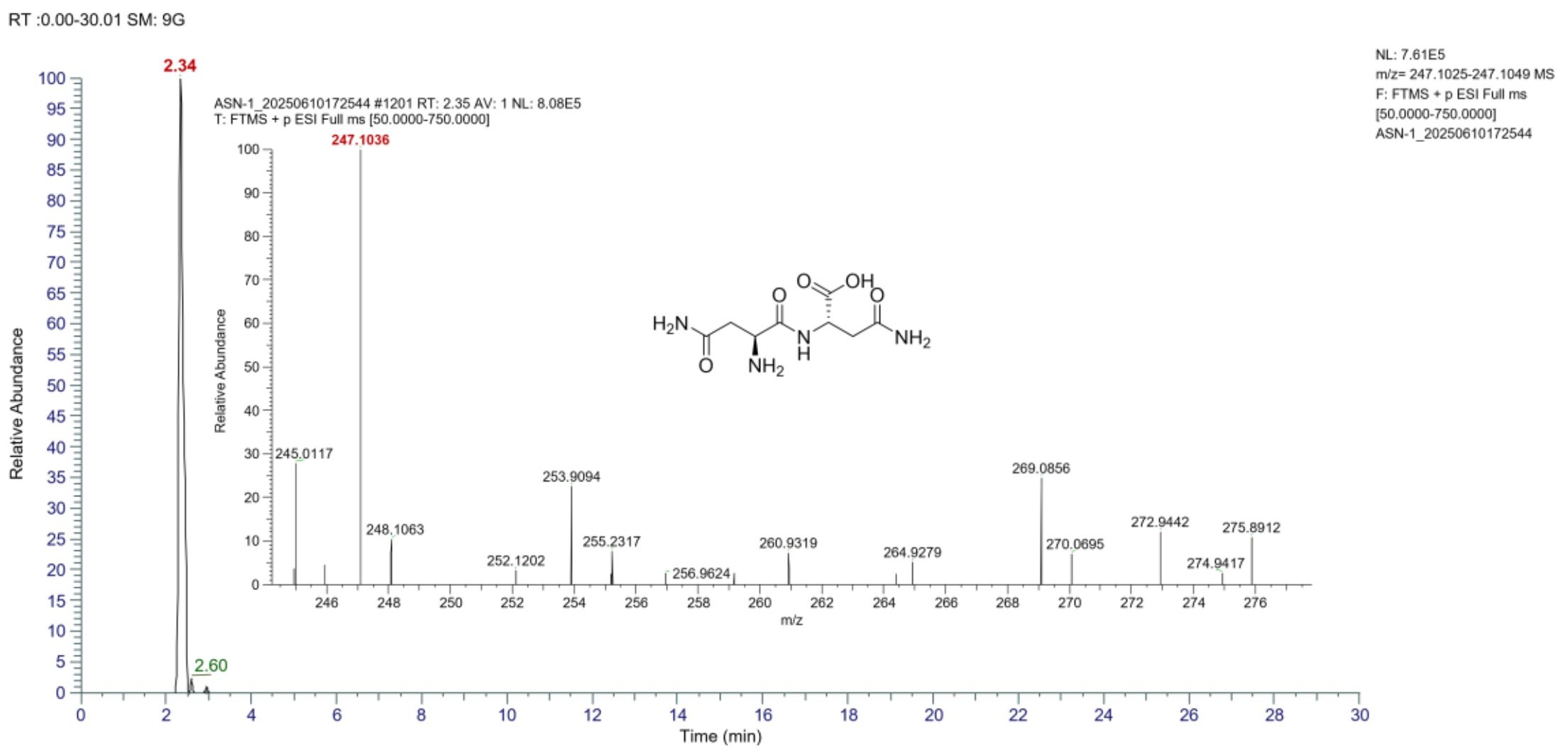

Fig. S26 The extraction ion chromatography-mass spectrometry (EIC-MS) data of Asn₂. The analysis results confirm that Asn_2 was formed in the reaction system containing Asn, CAP, and 0.25 M Mg^{2+}. The extraction ion chromatogram (EIC) shows a peak at m/z 247.1036, corresponding to Asn_2, with a retention time of 2.34 minutes.

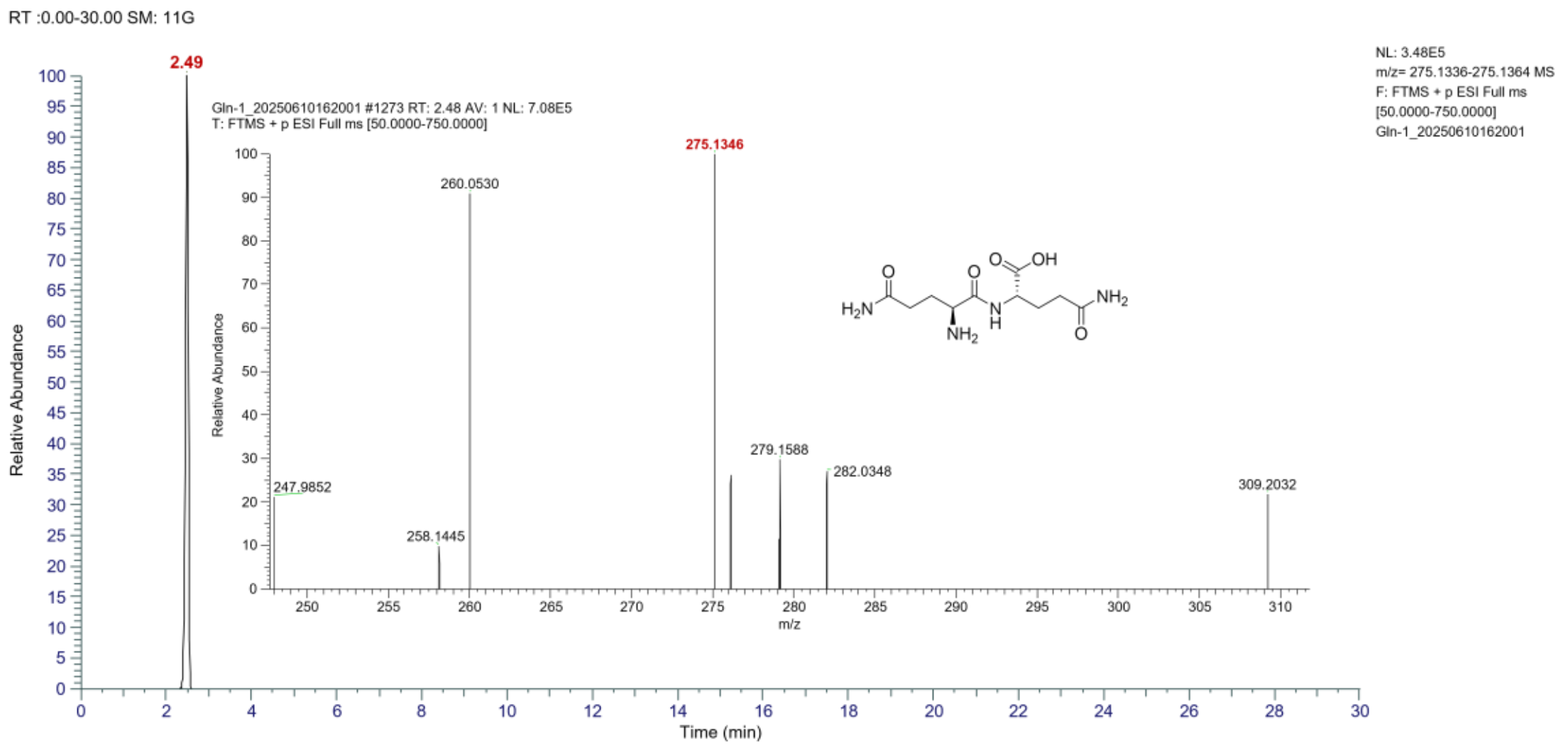

Fig. S27 The extraction ion chromatography-mass spectrometry (EIC-MS) data of Gln_2. The analysis results confirm that Gln_2 was formed in the reaction system containing Gln, CAP, and 0.25 M Mg^{2+}. The extraction ion chromatogram (EIC) shows a peak at m/z 275.1346, corresponding to Gln_2, with a retention time of 2.49 minutes.

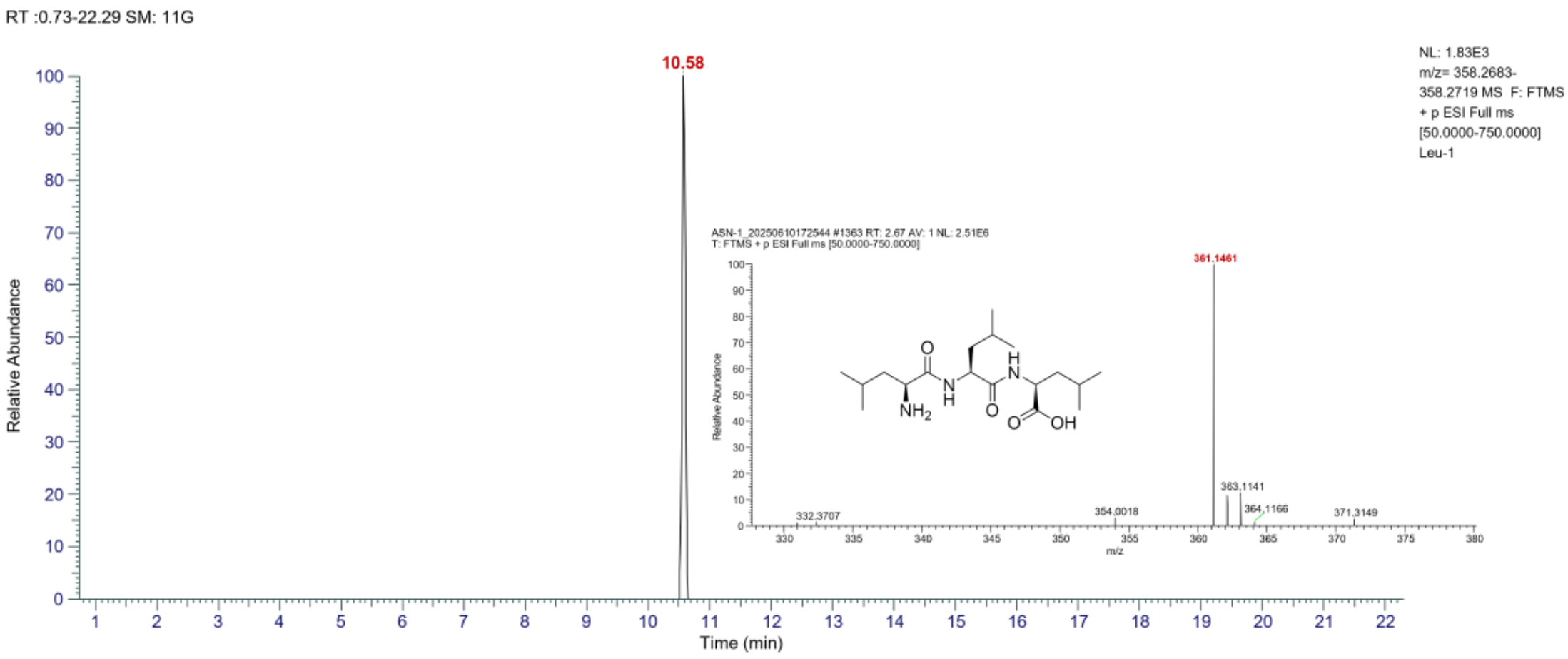

Fig. S28 The extraction ion chromatography-mass spectrometry (EIC-MS) data of Leu_3. The analysis results confirm that Leu_3 was formed in the reaction system containing Leu, CAP, and 0.25 M Mg^{2+}. The extraction ion chromatogram (EIC) shows a peak at m/z 358.2701, corresponding to Leu_3, with a retention time of 10.58 minutes.

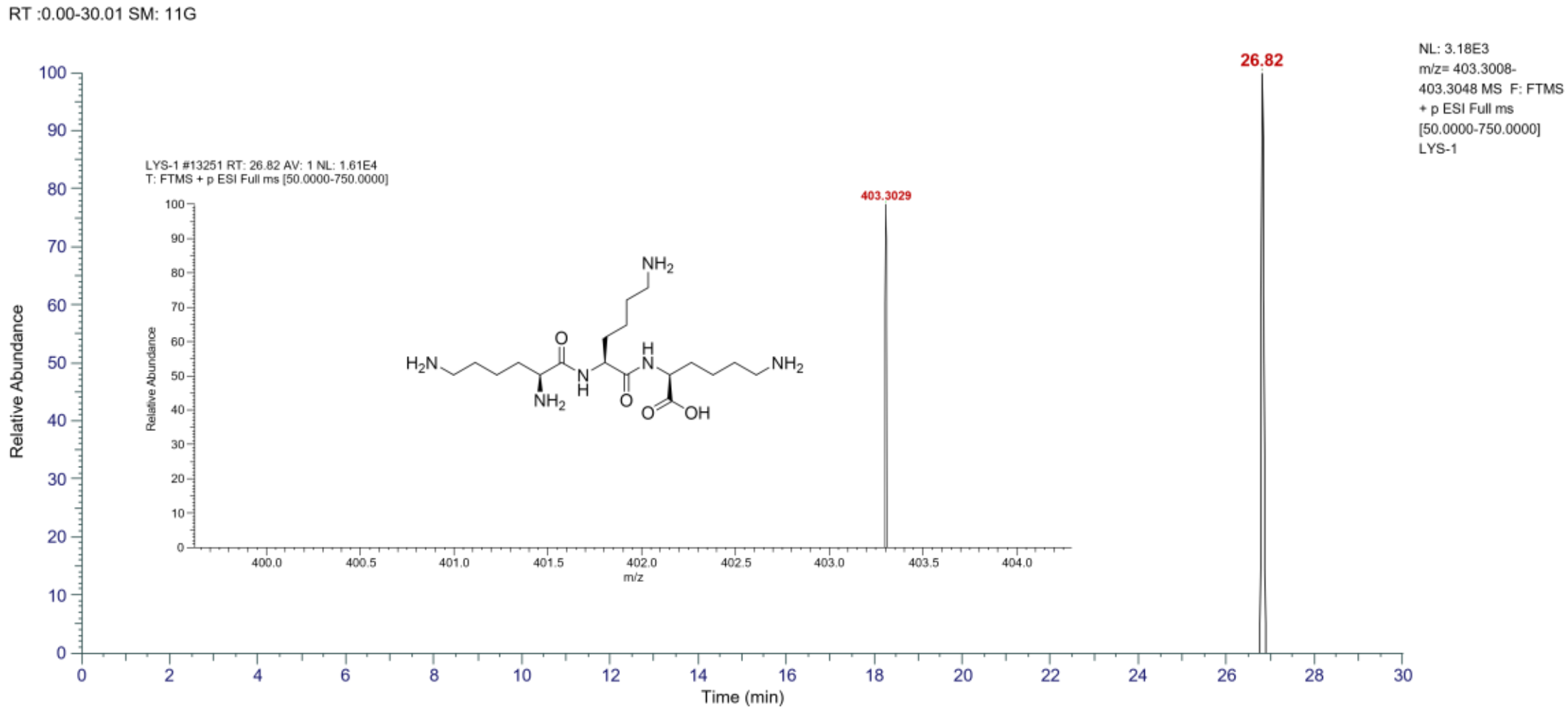

Fig. S29 The extraction ion chromatography-mass spectrometry (EIC-MS) data of Lys_3. The analysis results confirm that Lys_3 was formed in the reaction system containing Lys, CAP, and 0.25 M Mg^{2+}. The extraction ion chromatogram (EIC) shows a peak at m/z 403.3028, corresponding to Lys_3, with a retention time of 26.82 minutes

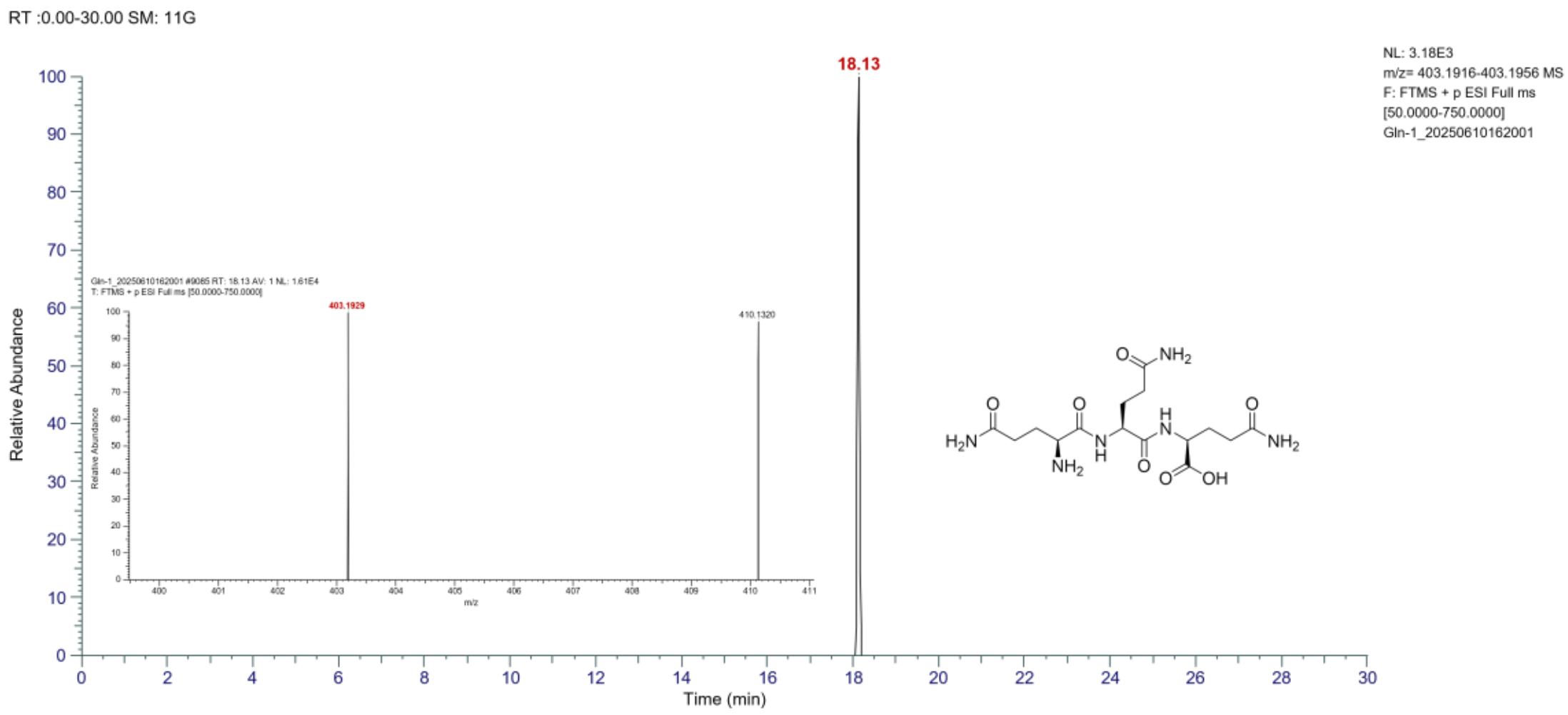

Fig. S30 The extraction ion chromatography-mass spectrometry (EIC-MS) data of Gln_3. The analysis results confirm that Gln_3 was formed in the reaction system containing Gln, CAP, and 0.25 M Mg^{2+}. The extraction ion chromatogram (EIC) shows a peak at m/z 403.1929, corresponding to Gln_3, with a retention time of 18.13 minutes.

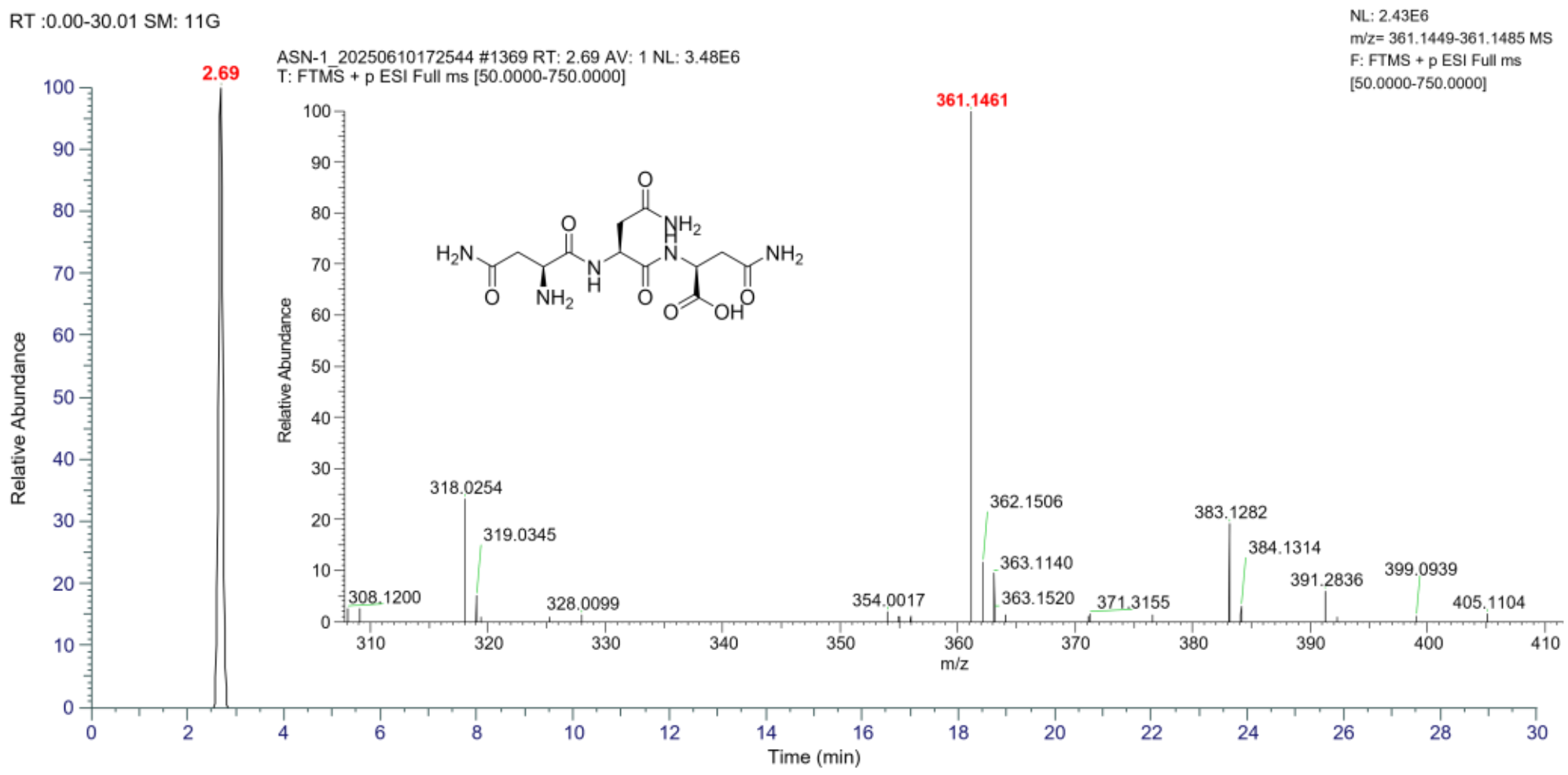

Fig. S31 The extraction ion chromatography-mass spectrometry (EIC-MS) data of Asn_3. The analysis results confirm that Asn_3 was formed in the reaction system containing Asn, CAP, and 0.25 M Mg^{2+}. The extraction ion chromatogram (EIC) shows a peak at m/z 361.1461, corresponding to Asn_3, with a retention time of 2.69 minutes.

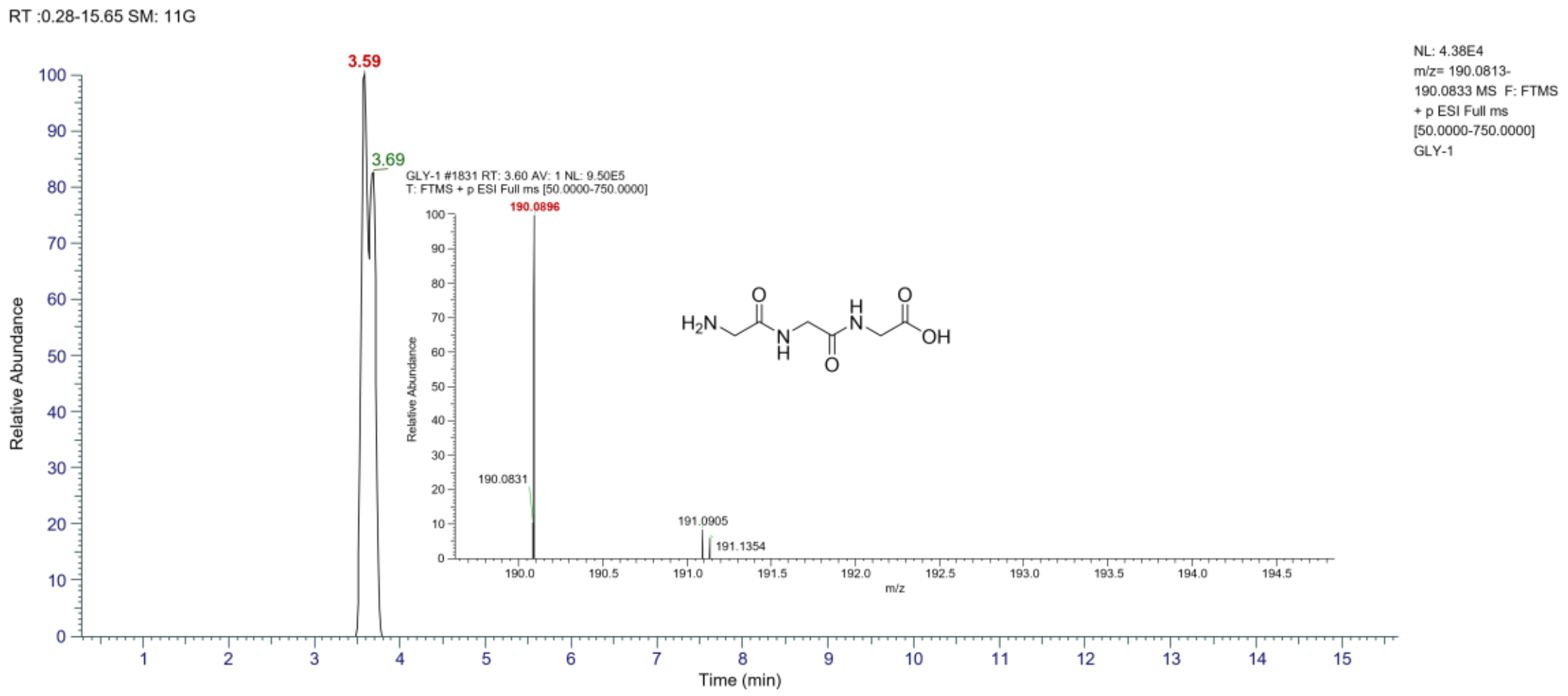

Fig. S32 The extraction ion chromatography-mass spectrometry (EIC-MS) data of Gly_3. The analysis results confirm that Gly_3 was formed in the reaction system containing Gly, CAP, and 0.25 M Mg^{2+}. The extraction ion chromatogram (EIC) shows a peak at m/z 190.0896, corresponding to Gly_3, with a retention time of 3.59 minutes.

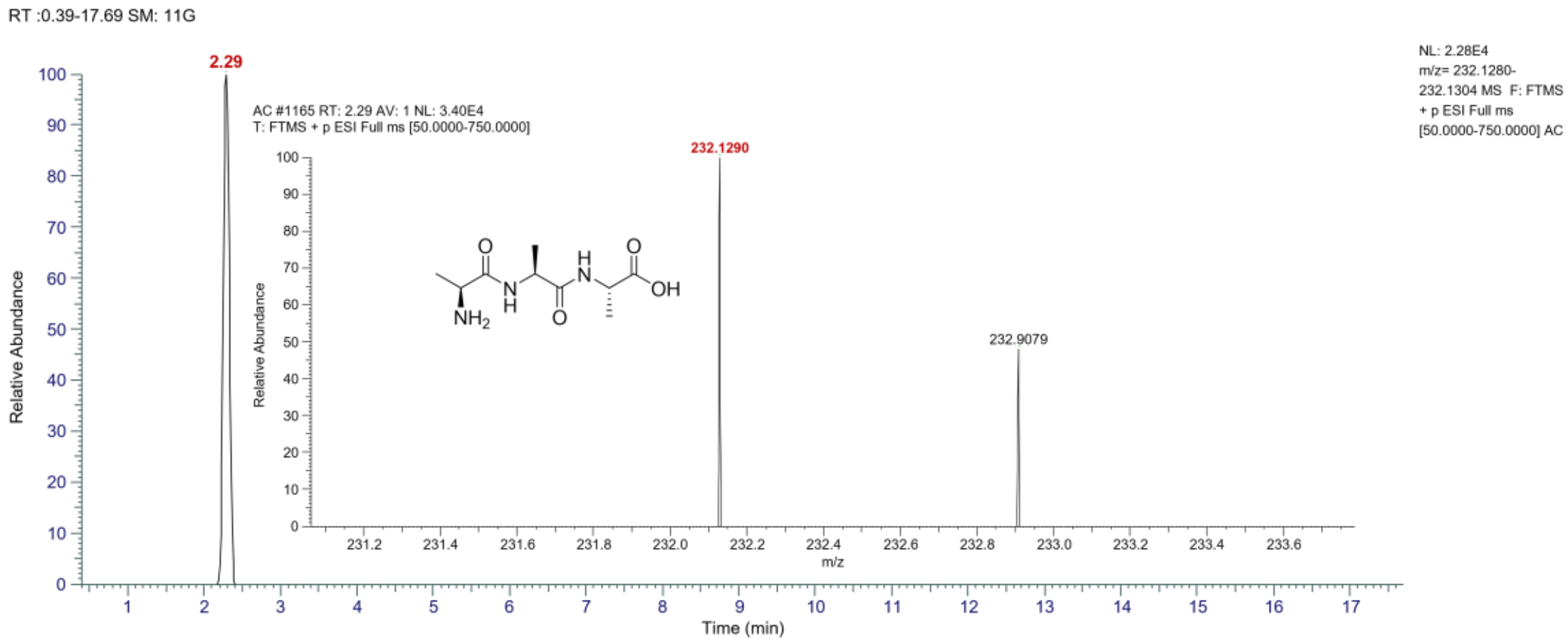

Fig. S33 The extraction ion chromatography-mass spectrometry (EIC-MS) data of Ala_3. The analysis results confirm that Ala_3 was formed in the reaction system containing Ala, CAP, and 0.25 M Mg^{2+}. The extraction ion chromatogram (EIC) shows a peak at m/z 232.1290, corresponding to Ala_3, with a retention time of 2.29 minutes.

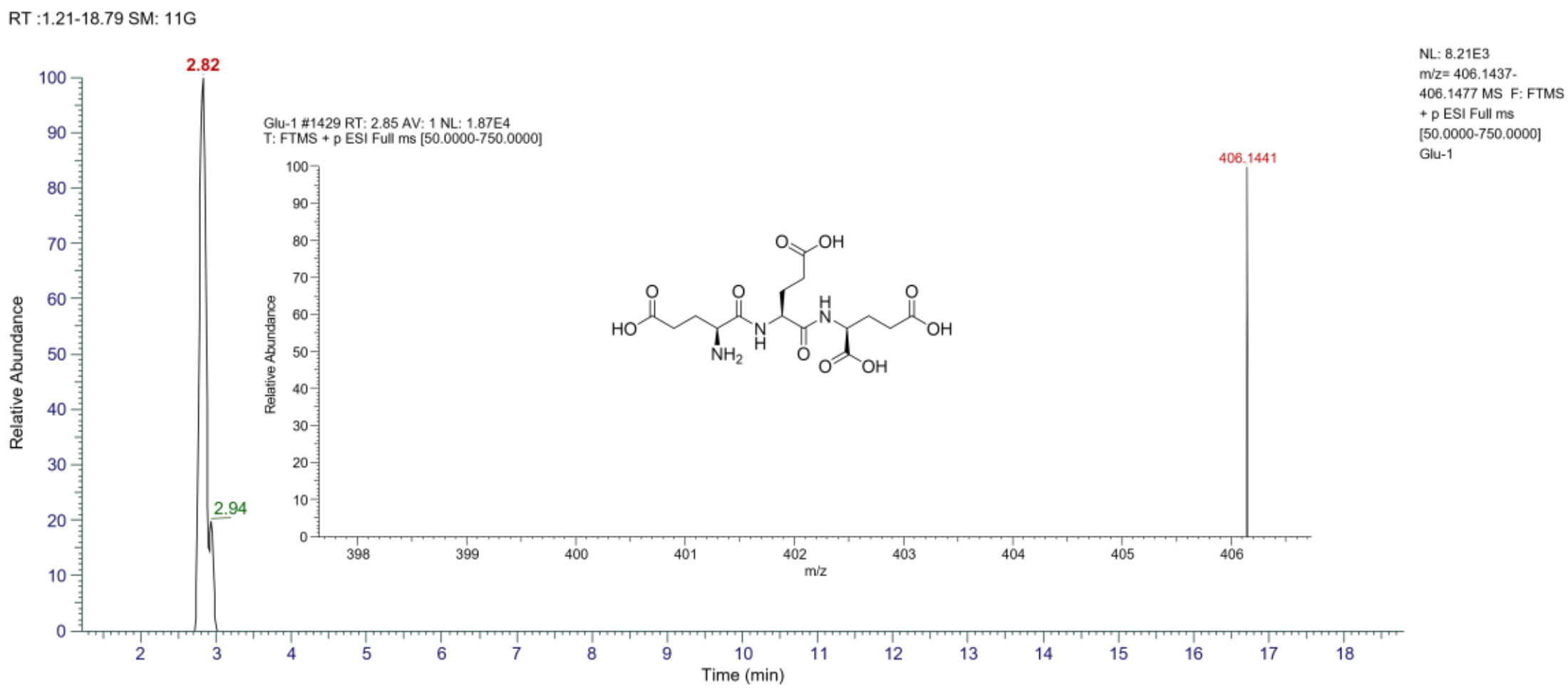

Fig. S34 The the extraction ion chromatography-mass spectrometry (EIC-MS) data of Glu_3. The analysis results confirm that Glu_3 was formed in the reaction system containing Glu, CAP, and 0.25 M Mg^{2+}. The extraction ion chromatogram (EIC) shows a peak at m/z 406.1441, corresponding to Glu_3, with a retention time of 2.82 minutes.

Diketopiperazines (DKPs) in products

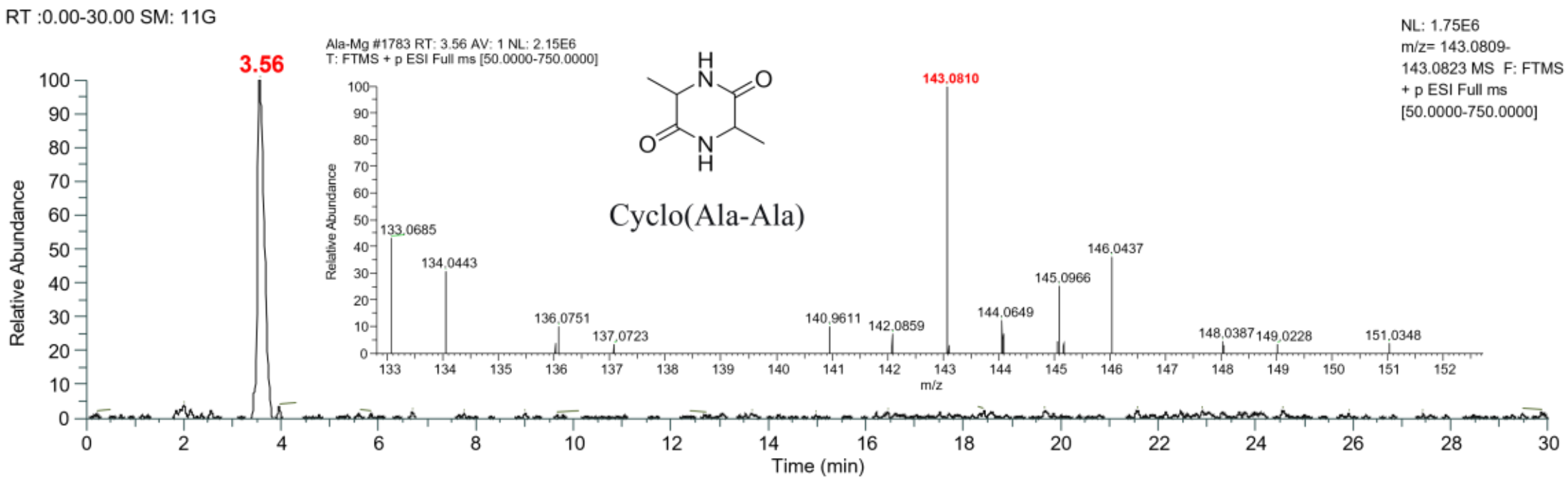

Fig. S35 presents the extraction ion chromatography-mass spectrometry (EIC-MS) data of Cyclo(Ala-Ala). The analysis results confirm that Cyclo(Ala-Ala) was formed in the reaction system containing Ala, CAP, and 0.25 M Mg^{2+}. The extraction ion chromatogram (EIC) shows a peak at m/z 143.0810, corresponding to Cyclo(Ala-Ala), with a retention time of 3.56 minutes.

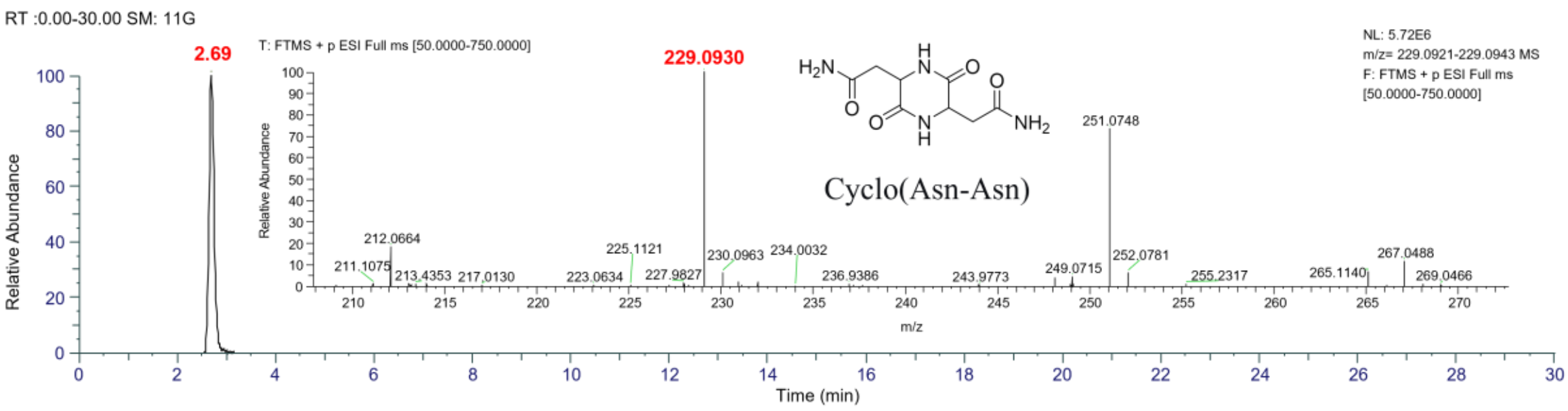

Fig. S36 presents the extraction ion chromatography-mass spectrometry (EIC-MS) data of Cyclo(Asn-Asn). The analysis results confirm that Cyclo(Asn-Asn) was formed in the reaction system containing Asn, CAP, and 0.25 M Mg^{2+}. The extraction ion chromatogram (EIC) shows a peak at m/z 229.0930, corresponding to Cyclo(Asn-Asn), with a retention time of 2.69 minutes.

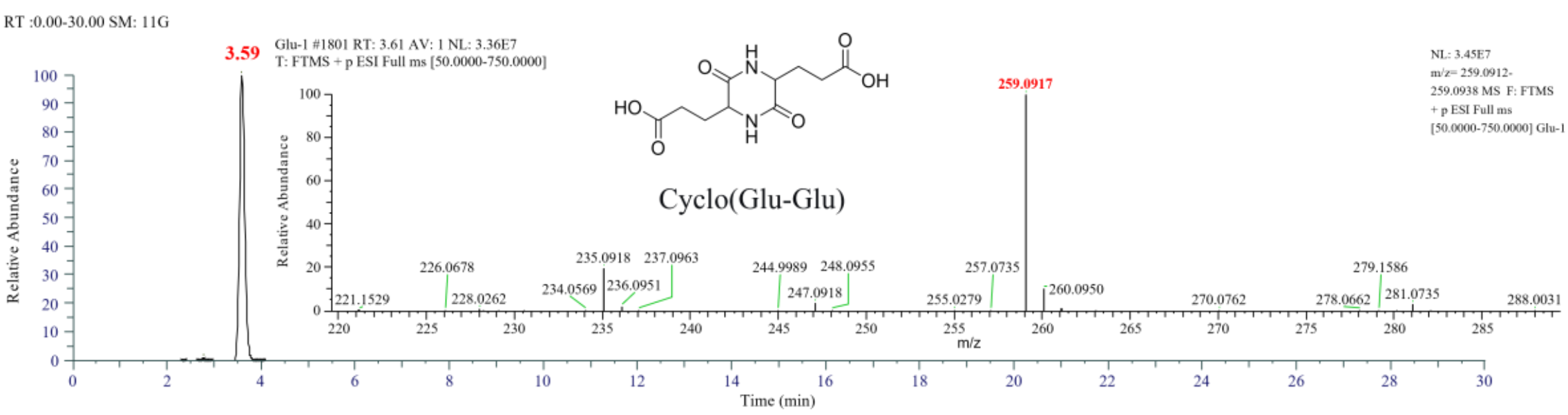

Fig. S37 presents the extraction ion chromatography-mass spectrometry (EIC-MS) data of Cyclo(Glu-Glu). The analysis results confirm that Cyclo(Glu-Glu) was formed in the reaction system containing Glu, CAP, and 0.25 M Mg^{2+}. The extraction ion chromatogram (EIC) shows a peak at m/z 259.0917, corresponding to Cyclo(Ala-Ala), with a retention time of 3.59 minutes.

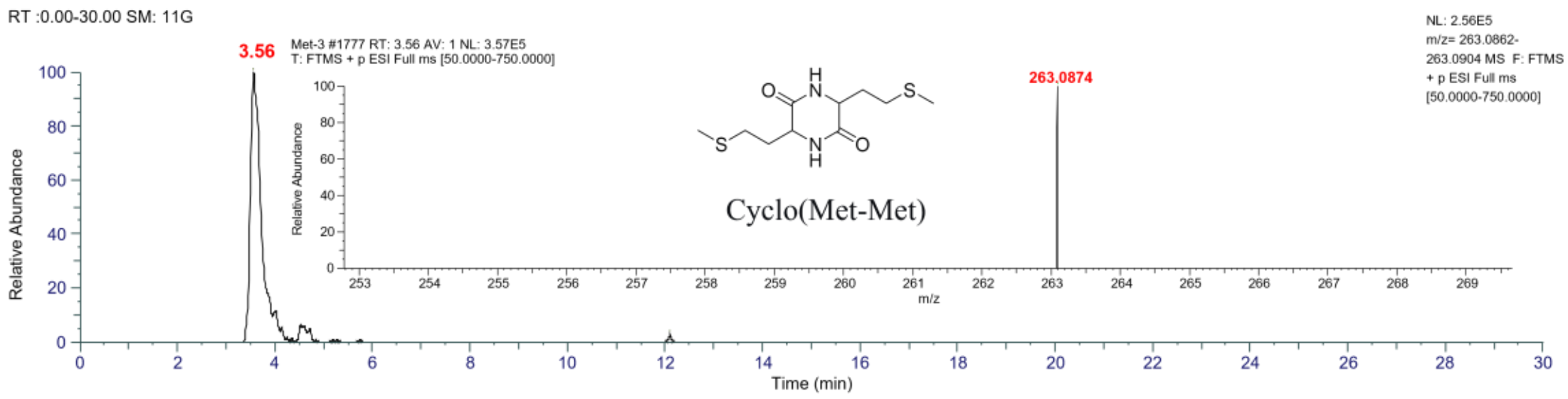

Fig. S38 presents the extraction ion chromatography-mass spectrometry (EIC-MS) data of Cyclo(Met-Met). The analysis results confirm that Cyclo(Met-Met) was formed in the reaction system containing Met, CAP, and 0.25 M Mg^{2+}. The extraction ion chromatogram (EIC) shows a peak at m/z 263.0874, corresponding to Cyclo(Met-Met), with a retention time of 3.56 minutes.

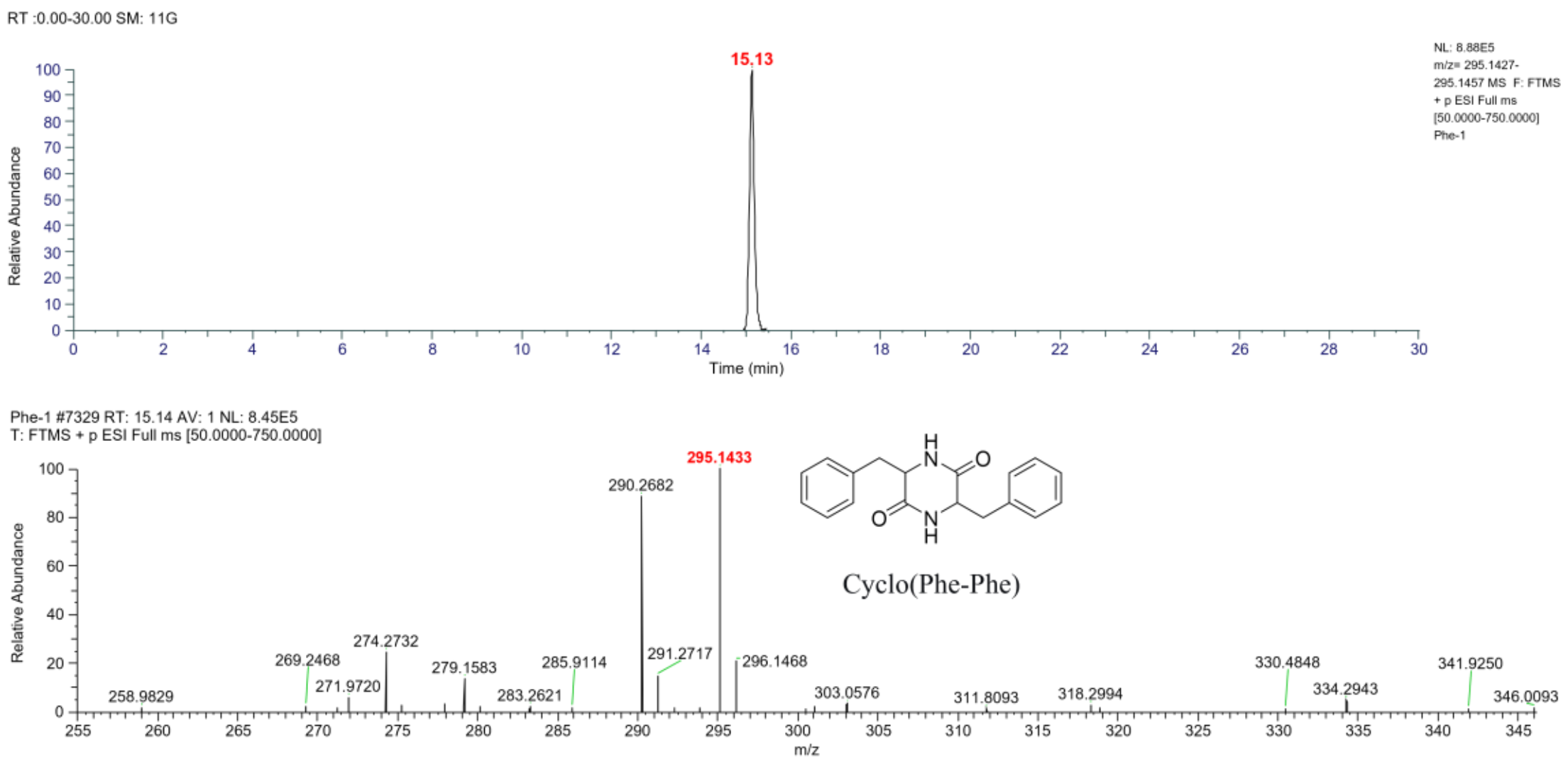

Fig. S39 presents the extraction ion chromatography-mass spectrometry (EIC-MS) data of Cyclo(Phe-Phe). The analysis results confirm that Cyclo(Phe-Phe) was formed in the reaction system containing Glu, CAP, and 0.25 M Mg^{2+}. The extraction ion chromatogram (EIC) shows a peak at m/z 295.1433, corresponding to Cyclo(Phe-Phe), with a retention time of 15.13 minutes.

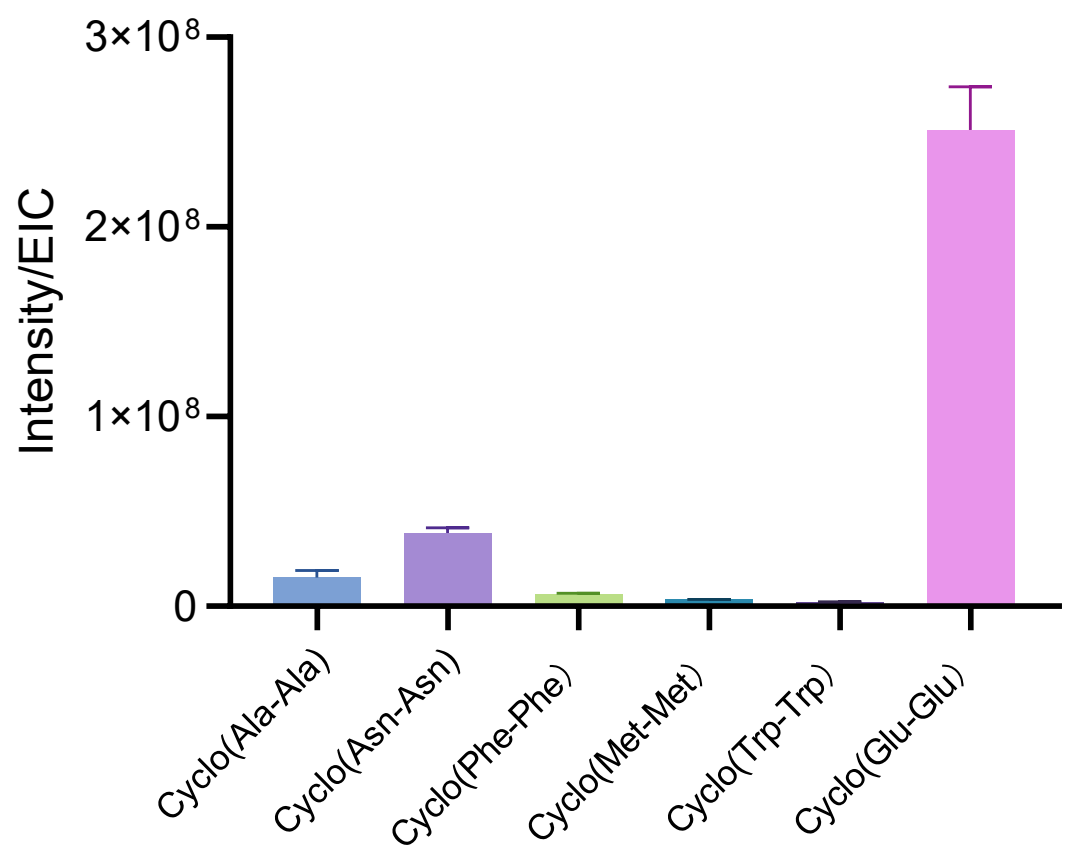

Fig. S40 Summary on DKPs formation.

N-Carbamoyl amino acids (CAAs) in products

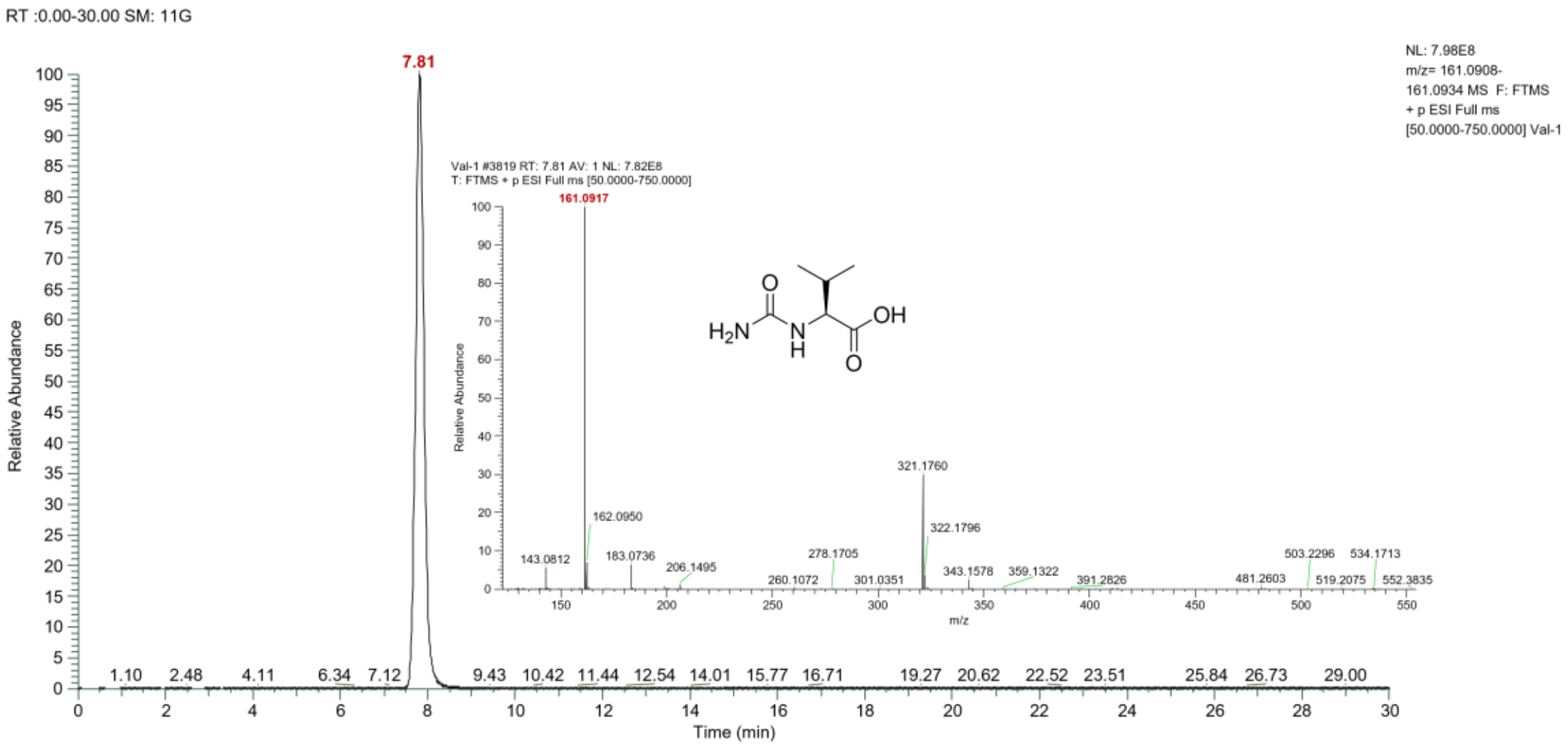

Fig. S41 The extraction ion chromatography-mass spectrometry (EIC-MS) data of Carbamoyl-Val. The analysis results confirm that Carbamoyl-Val was formed in the reaction system containing Val, CAP and 0.25 M Mg^{2+}. The extraction ion chromatogram (EIC) shows a peak at m/z 161.0917, corresponding to Carbamoyl-Val, with a retention time of 7.81 minutes.

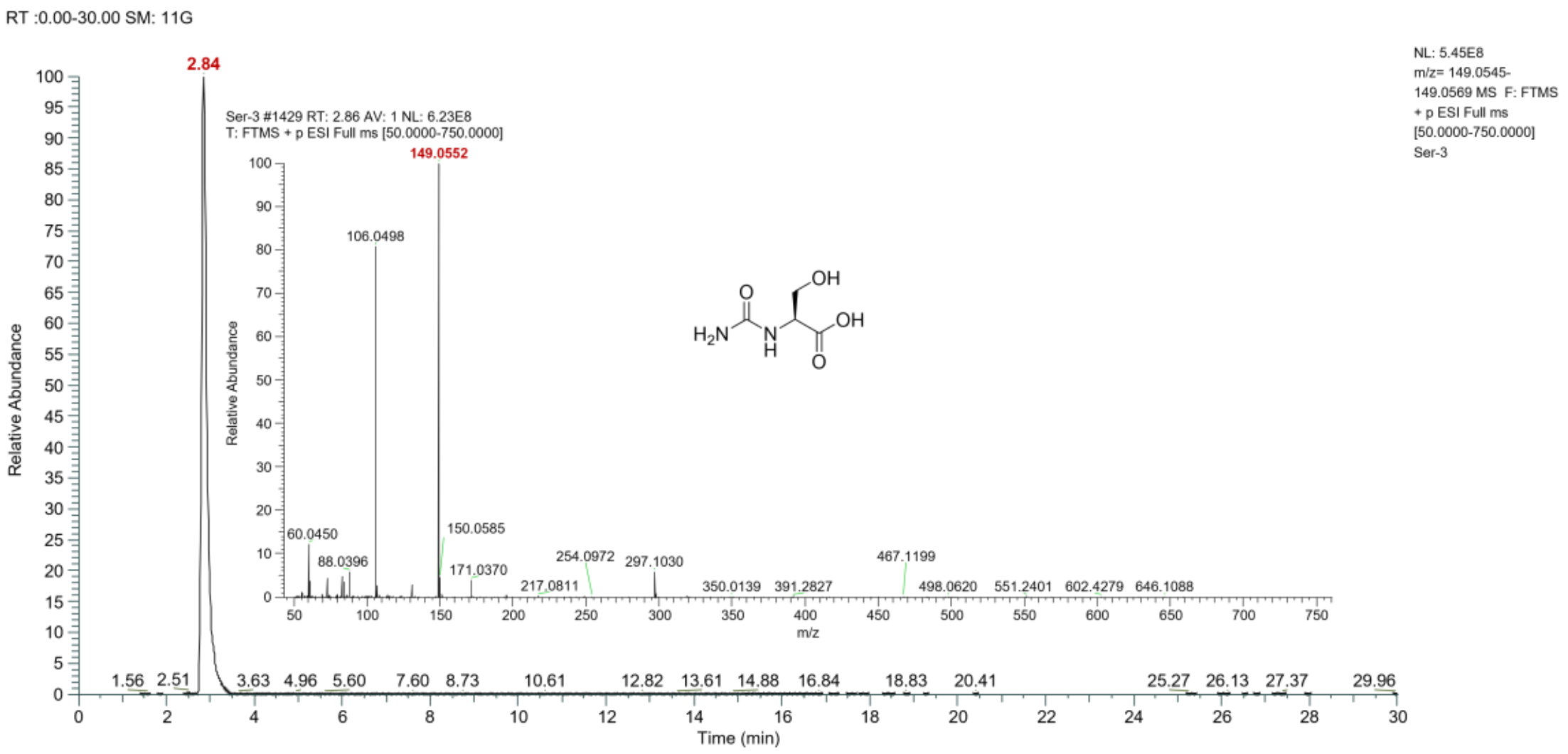

Fig. S42 The extraction ion chromatography-mass spectrometry (EIC-MS) data of Carbamoyl-Ser. The analysis results confirm that Carbamoyl-Ser was formed in the reaction system containing Ser, CAP and 0.25 M Mg^{2+}. The extraction ion chromatogram (EIC) shows a peak at m/z 149.0552, corresponding to Carbamoyl-Ser, with a retention time of 2.84 minutes.

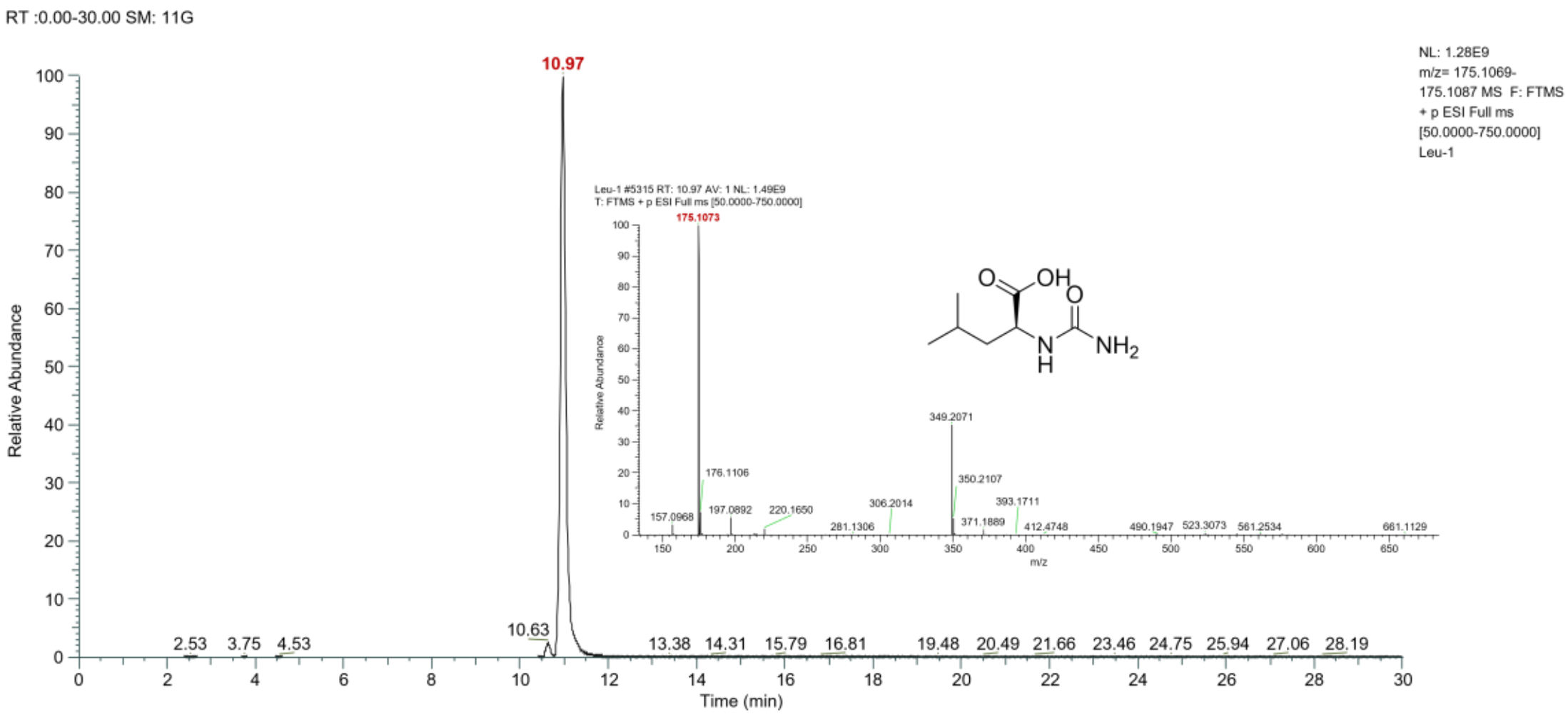

Fig. S43 The extraction ion chromatography-mass spectrometry (EIC-MS) data of Carbamoyl-Leu. The analysis results confirm that Carbamoyl-Leu was formed in the reaction system containing Leu, CAP and 0.25 M Mg^{2+}. The extraction ion chromatogram (EIC) shows a peak at m/z 175.1073, corresponding to Carbamoyl-Leu, with a retention time of 10.97 minutes.

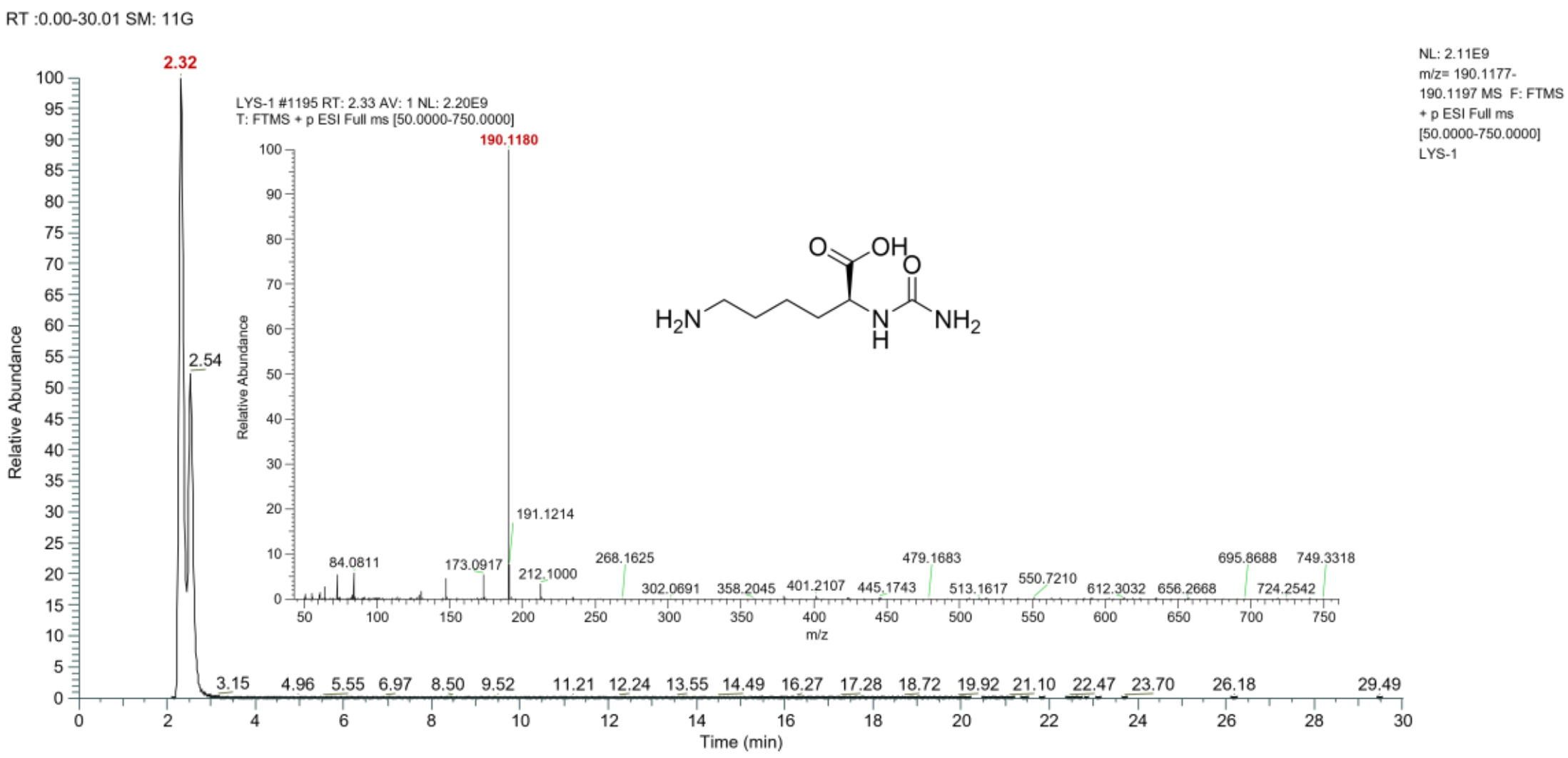

Fig. S44 The extraction ion chromatography-mass spectrometry (EIC-MS) data of Carbamoyl-Lys. The analysis results confirm that Carbamoyl-Lys was formed in the reaction system containing Lys, CAP and 0.25 M Mg^{2+}. The extraction ion chromatogram (EIC) shows a peak at m/z 190.1180, corresponding to Carbamoyl-Lys, with a retention time of 2.32 minutes.

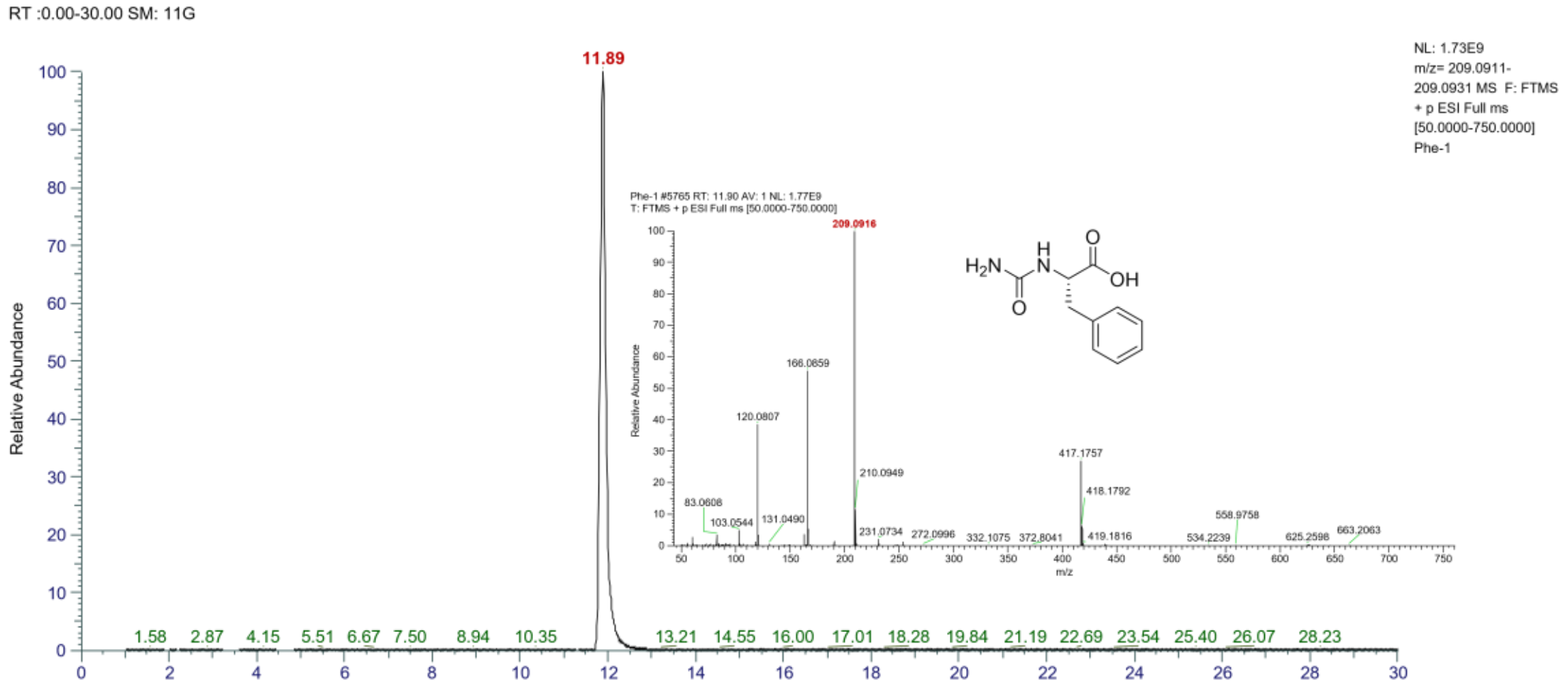

Fig. S45 The extraction ion chromatography-mass spectrometry (EIC-MS) data of Carbamoyl-Phe. The analysis results confirm that Carbamoyl-Phe was formed in the reaction system containing Phe, CAP and 0.25 M Mg^{2+}. The extraction ion chromatogram (EIC) shows a peak at m/z 209.0916, corresponding to Carbamoyl-Phe, with a retention time of 11.89 minutes.

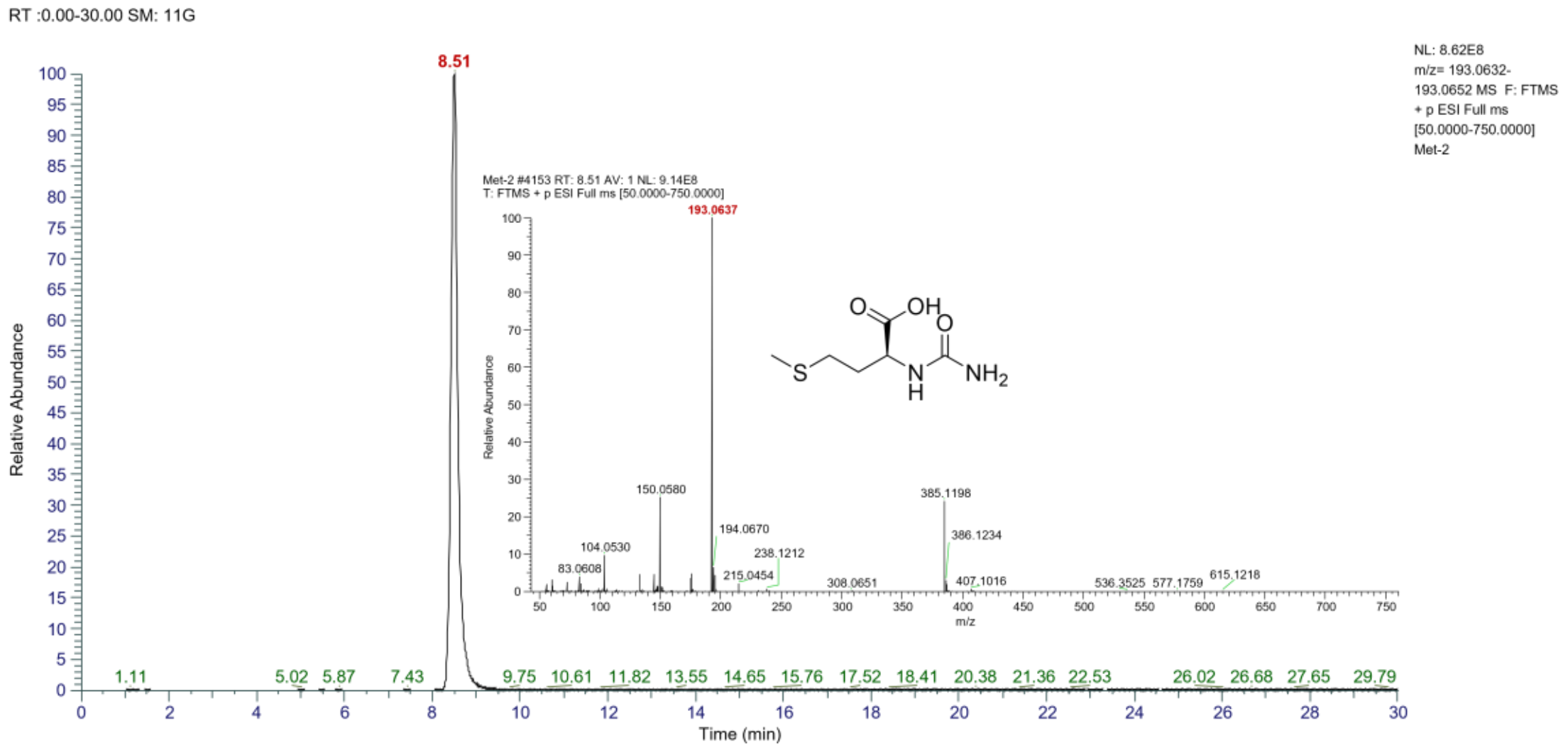

Fig. S46 The extraction ion chromatography-mass spectrometry (EIC-MS) data of Carbamoyl-Met. The analysis results confirm that Carbamoyl-Met was formed in the reaction system containing Met, CAP and 0.25 M Mg^{2+}. The extraction ion chromatogram (EIC) shows a peak at m/z 193.0637, corresponding to Carbamoyl-Met, with a retention time of 8.51 minutes.

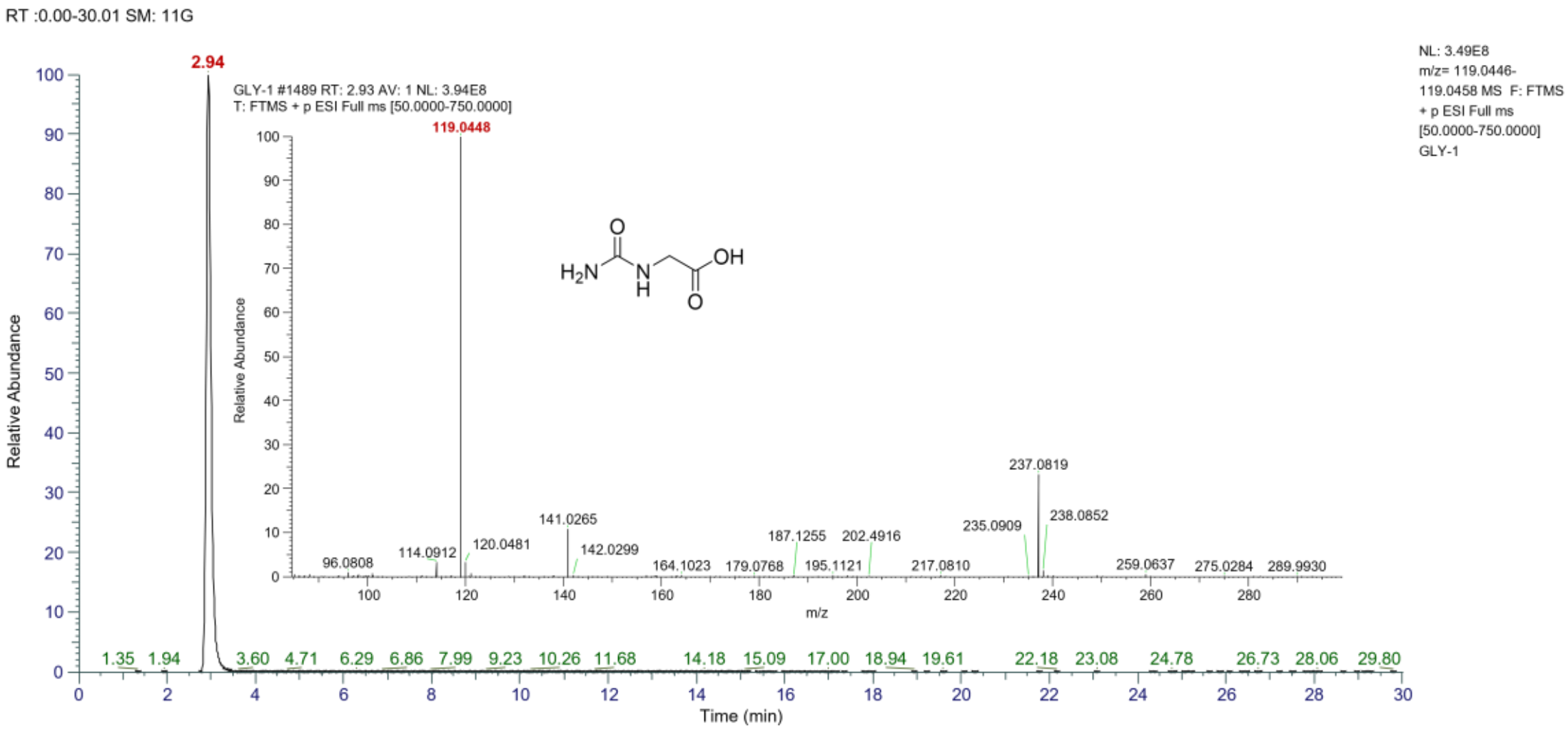

Fig. S47 The extraction ion chromatography-mass spectrometry (EIC-MS) data of Carbamoyl-Gly. The analysis results confirm that Carbamoyl-Gly was formed in the reaction system containing Gly, CAP and 0.25 M Mg^{2+}. The extraction ion chromatogram (EIC) shows a peak at m/z 119.0448, corresponding to Carbamoyl-Gly, with a retention time of 2.94 minutes.

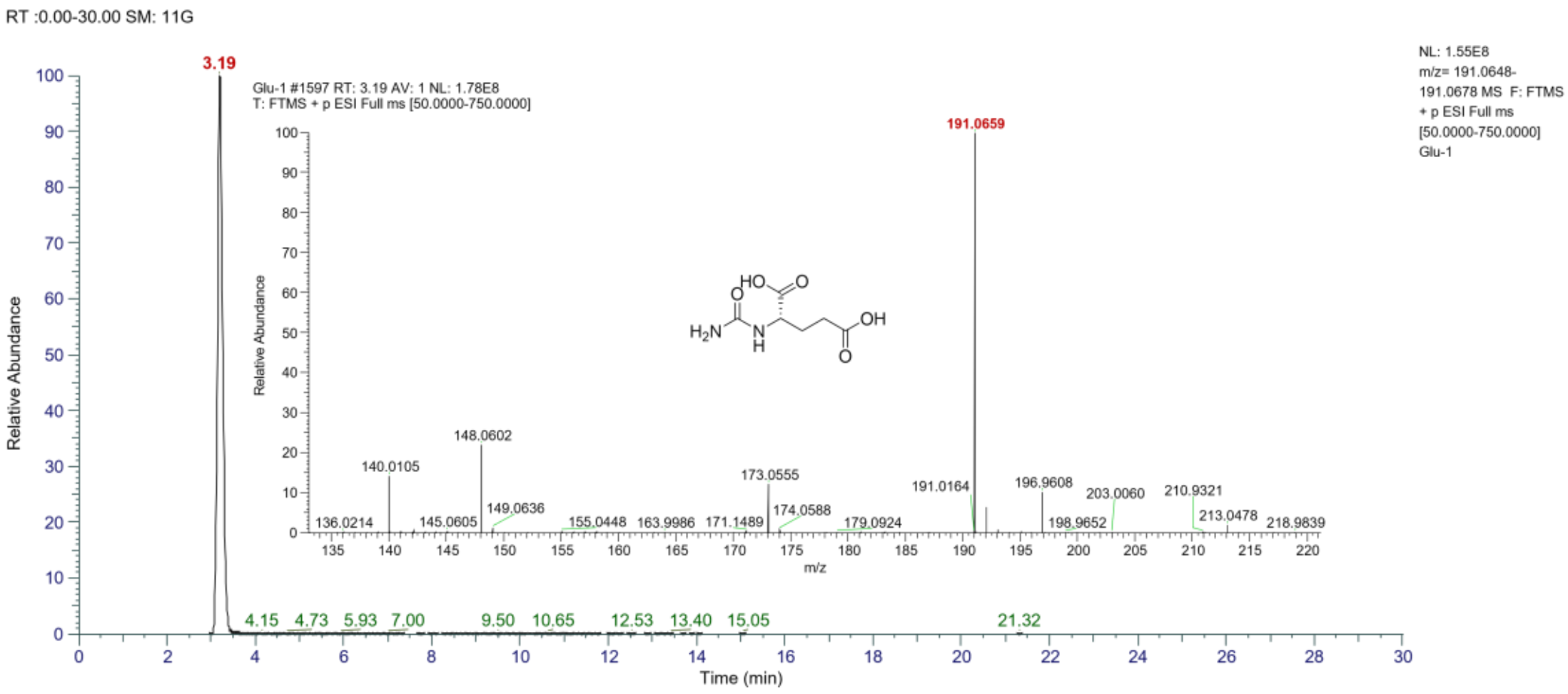

Fig. S48 The extraction ion chromatography-mass spectrometry (EIC-MS) data of Carbamoyl-Glu. The analysis results confirm that Carbamoyl-Glu was formed in the reaction system containing Glu, CAP and 0.25 M Mg^{2+}. The extraction ion chromatogram (EIC) shows a peak at m/z 191.0659, corresponding to Carbamoyl-Glu, with a retention time of 3.19 minutes.

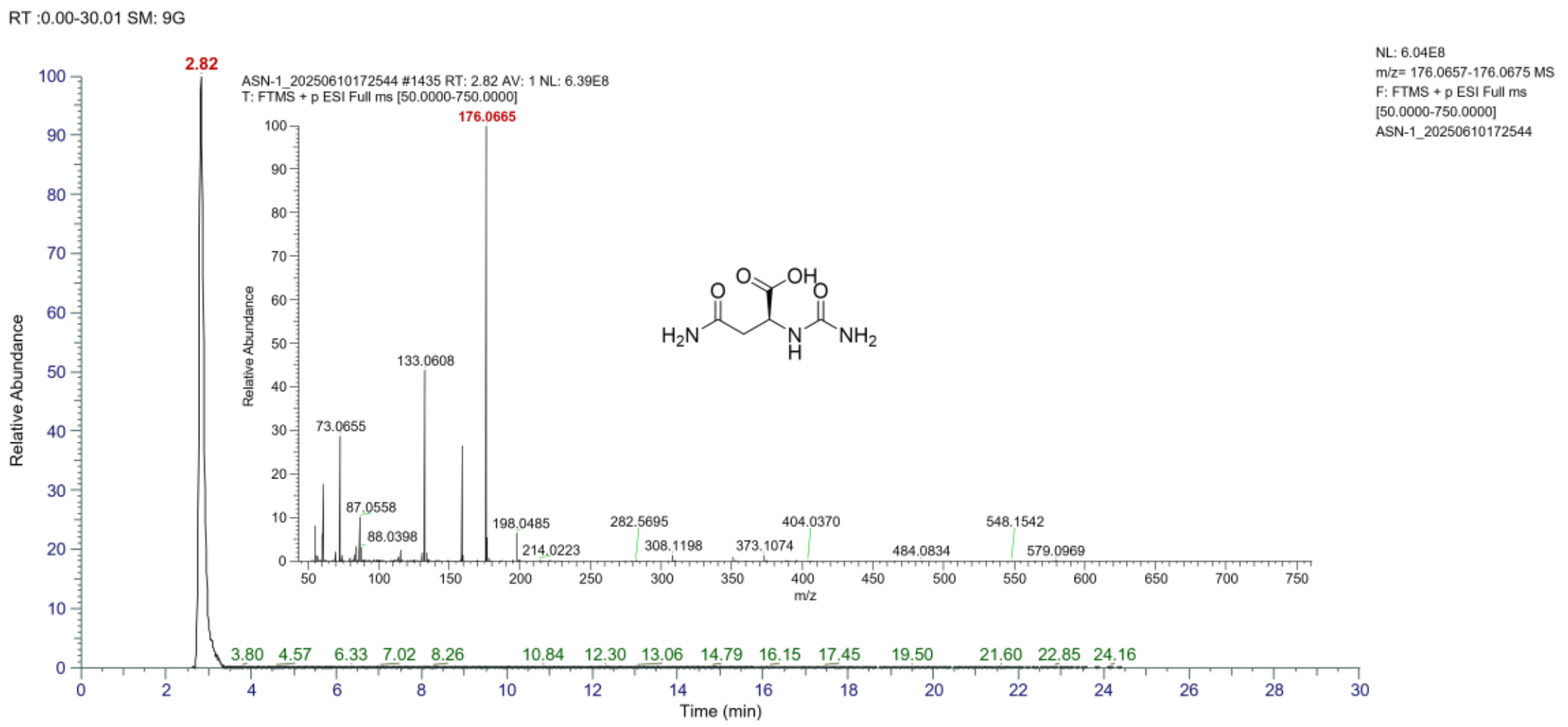

Fig. S49 The extraction ion chromatography-mass spectrometry (EIC-MS) data of Carbamoyl-Asn. The analysis results confirm that Carbamoyl-Asn was formed in the reaction system containing Asn, CAP and 0.25 M Mg^{2+}. The extraction ion chromatogram (EIC) shows a peak at m/z 176.0665, corresponding to Carbamoyl-Asn, with a retention time of 2.82 minutes.

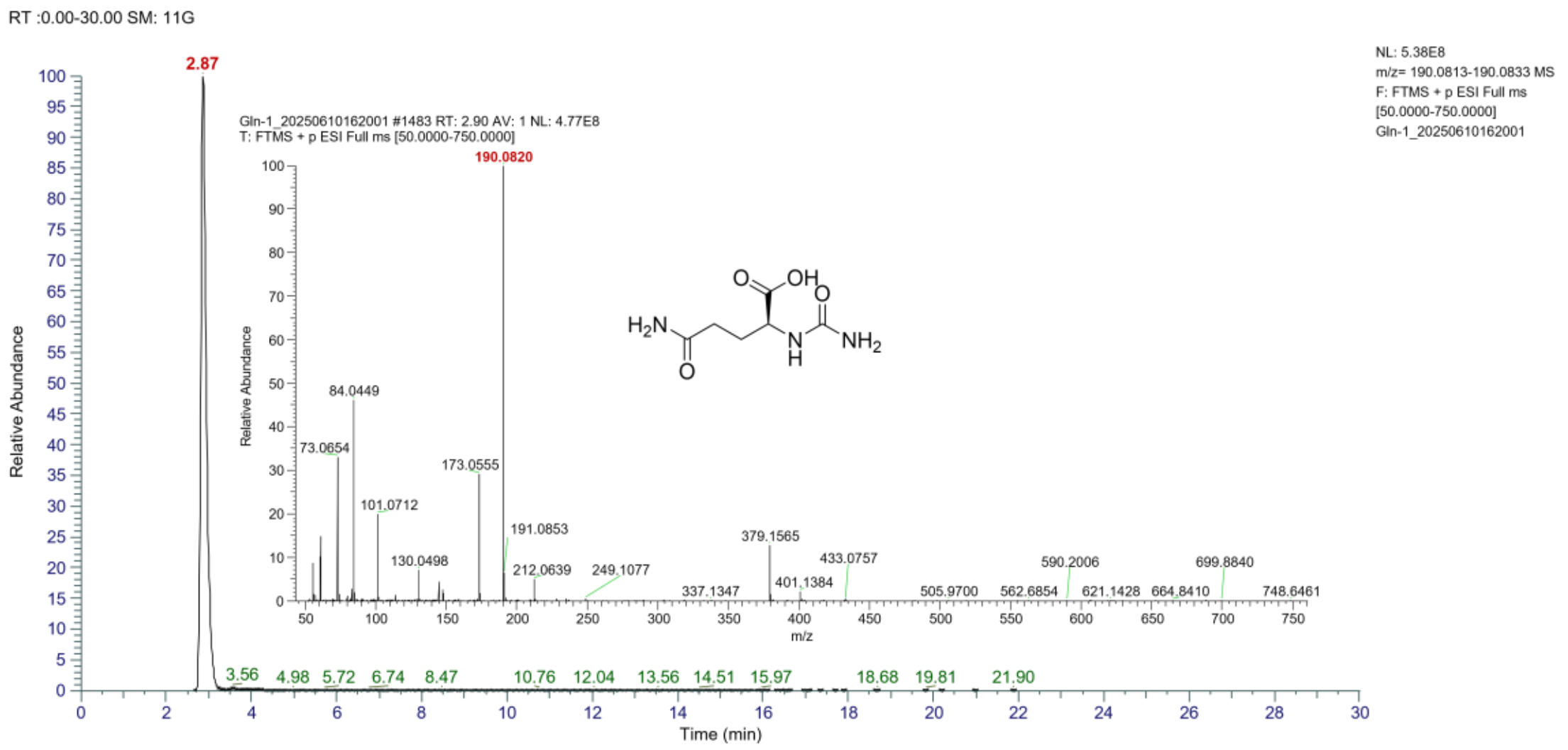

Fig. S50 The extraction ion chromatography-mass spectrometry (EIC-MS) data of Carbamoyl-Gln. The analysis results confirm that Carbamoyl-Gln was formed in the reaction system containing Gln, CAP and 0.25 M Mg^{2+}. The extraction ion chromatogram (EIC) shows a peak at m/z 190.0820, corresponding to Carbamoyl-Gln, with a retention time of 2.87 minutes.

N-carboxyanhydrides (NCAs) in products

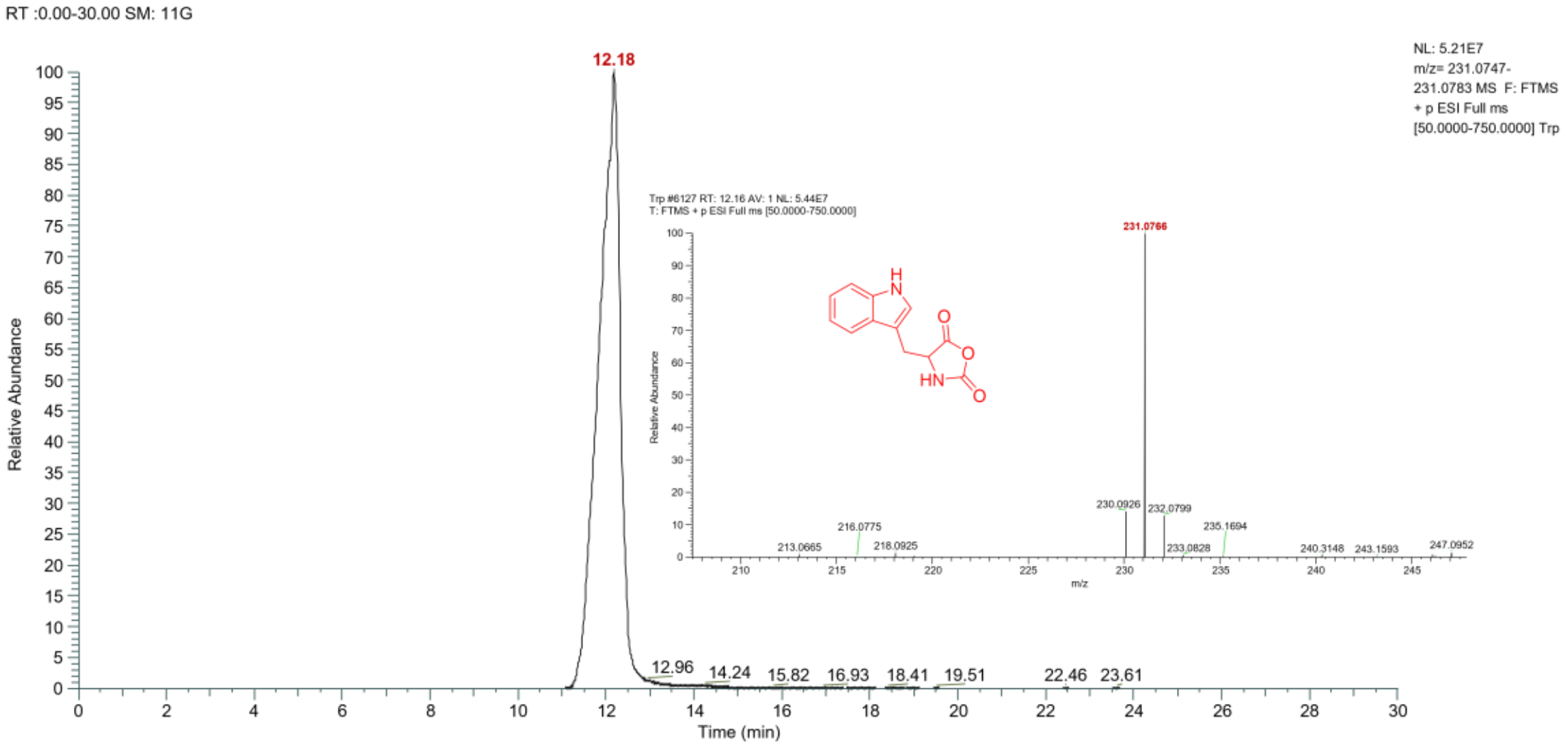

Fig. S51 The extraction ion chromatography-mass spectrometry (EIC-MS) data of N-carboxy [Trp] anhydride (NCA). The analysis results confirm that N-carboxy [Trp] anhydride (NCA) was formed in the reaction system containing Trp, CAP and 0.25 M Mg^{2+}. The extraction ion chromatogram (EIC) shows a peak at m/z 231.0766, corresponding to N-carboxy [Trp] anhydride (NCA), with a retention time of 12.18 minutes.

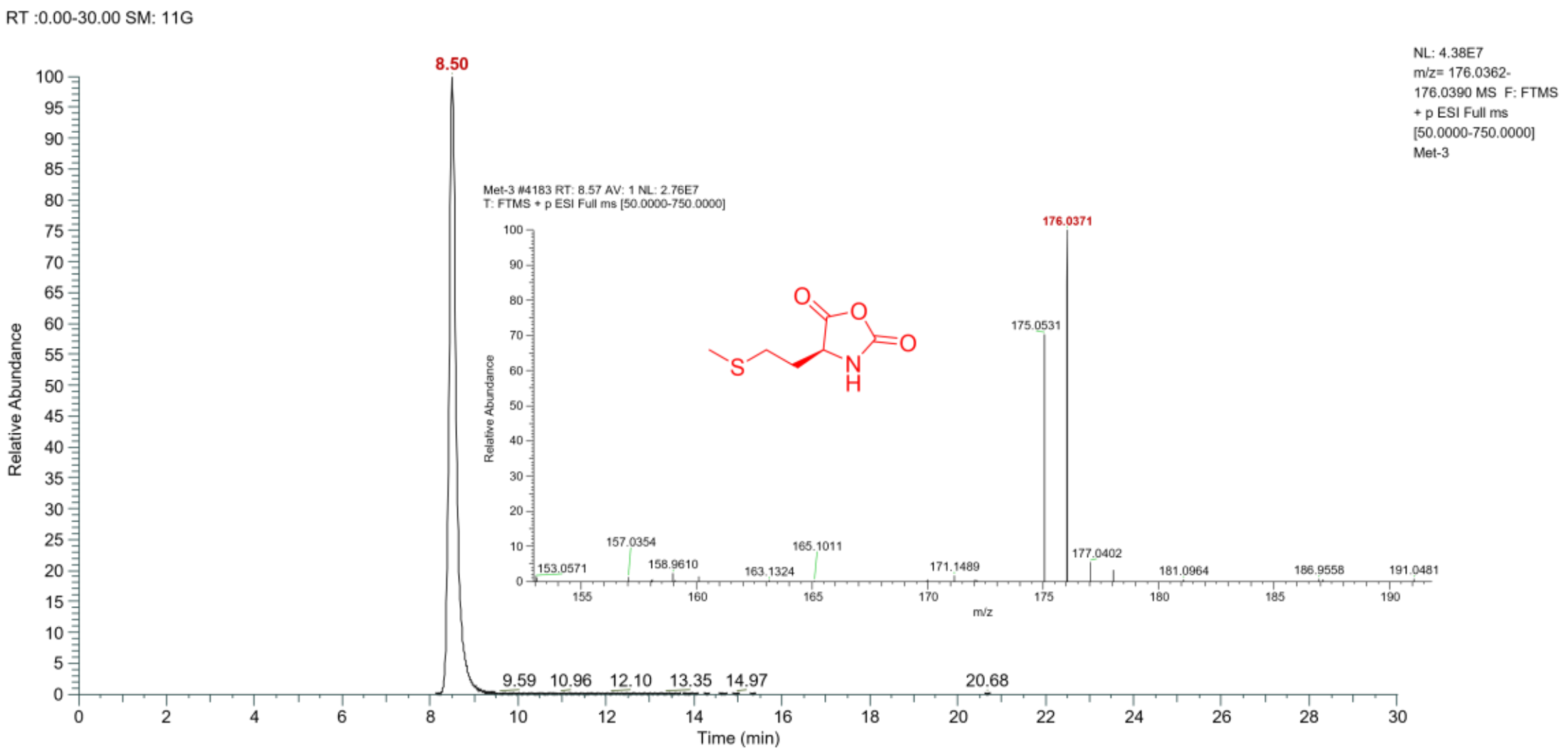

Fig. S52 The extraction ion chromatography-mass spectrometry (EIC-MS) data of N-carboxy [Met] anhydride (NCA). The analysis results confirm that N-carboxy [Met] anhydride (NCA) was formed in the reaction system containing Met, CAP and 0.25 M Mg^{2+}. The extraction ion chromatogram (EIC) shows a peak at m/z 176.0371, corresponding to N-carboxy [Met] anhydride (NCA), with a retention time of 8.50 minutes.

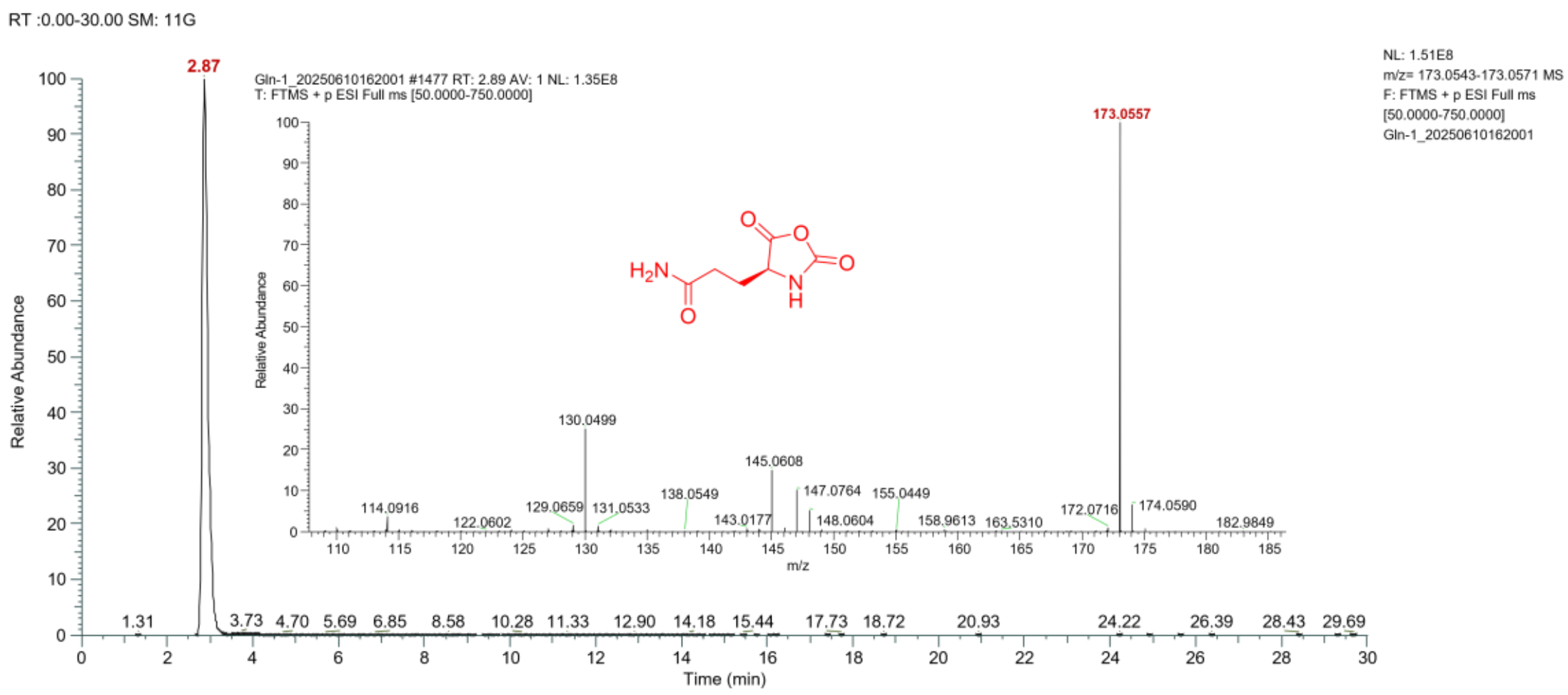

Fig. S53 The extraction ion chromatography-mass spectrometry (EIC-MS) data of N-carboxy [Gln] anhydride (NCA). The analysis results confirm that N-carboxy [Gln] anhydride (NCA) was formed in the reaction system containing Gln, CAP and 0.25 M Mg^{2+}. The extraction ion chromatogram (EIC) shows a peak at m/z 173.0557, corresponding to N-carboxy [Gln] anhydride (NCA), with a retention time of 2.87 minutes.

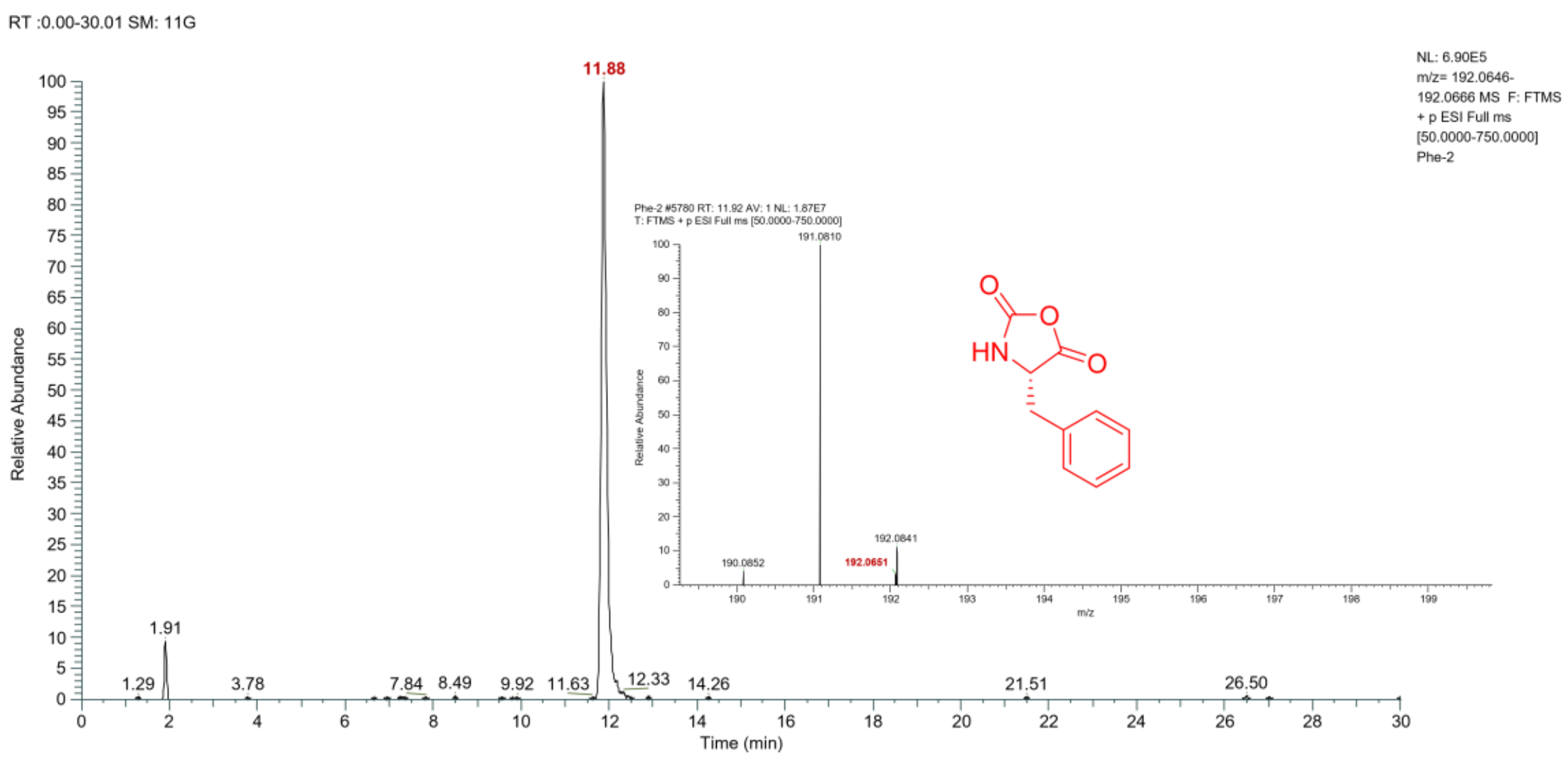

Fig. S54 The extraction ion chromatography-mass spectrometry (EIC-MS) data of N-carboxy [Phe] anhydride (NCA). The analysis results confirm that N-carboxy [Phe] anhydride (NCA) was formed in the reaction system containing Phe, CAP and 0.25 M Mg^{2+}. The extraction ion chromatogram (EIC) shows a peak at m/z 192.0651, corresponding to N-carboxy [Phe] anhydride (NCA), with a retention time of 11.88 minutes.

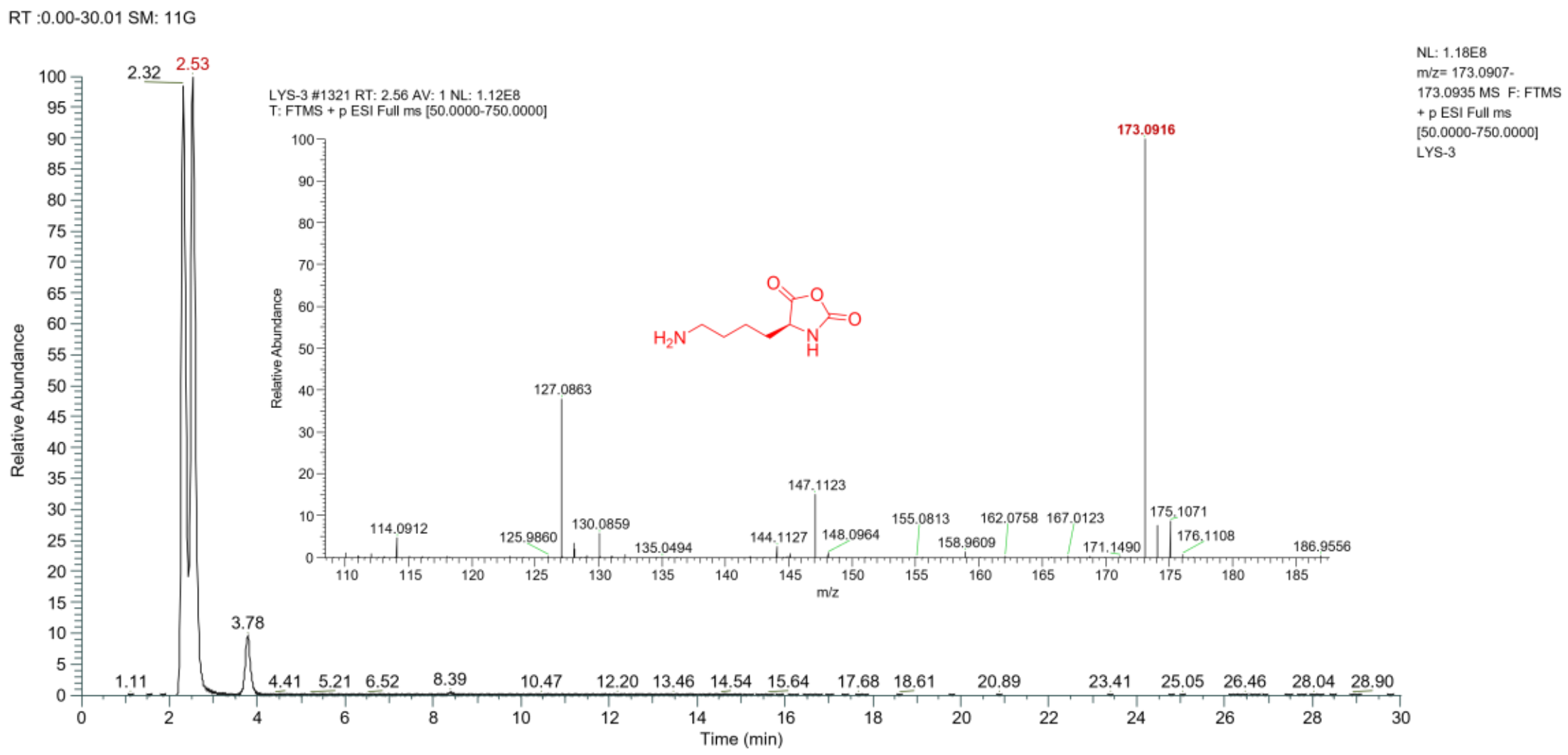

Fig. S55 The extraction ion chromatography-mass spectrometry (EIC-MS) data of N-carboxy [Lys] anhydride (NCA). The analysis results confirm that N-carboxy [Lys] anhydride (NCA) was formed in the reaction system containing Lys, CAP and 0.25 M Mg^{2+}. The extraction ion chromatogram (EIC) shows a peak at m/z 173.0916, corresponding to N-carboxy [Lys] anhydride (NCA), with a retention time of 2.53 minutes.

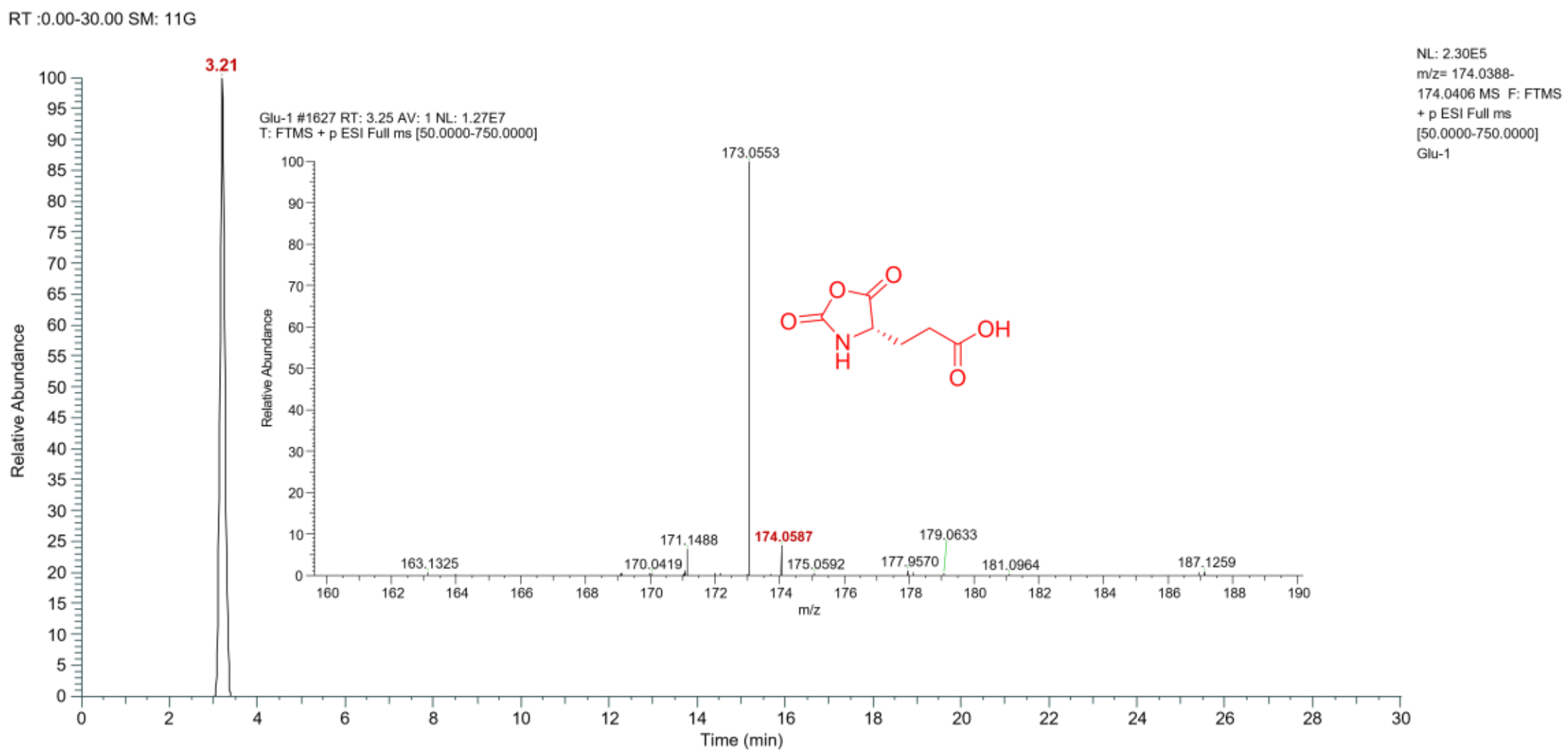

Fig. S56 The extraction ion chromatography-mass spectrometry (EIC-MS) data of N-carboxy [Glu] anhydride (NCA). The analysis results confirm that N-carboxy [Glu] anhydride (NCA) was formed in the reaction system containing Glu, CAP and 0.25 M Mg^{2+}. The extraction ion chromatogram (EIC) shows a peak at m/z 174.0587, corresponding to N-carboxy [Glu] anhydride (NCA), with a retention time of 3.21 minutes.

O-phosphoryl amino acid in products

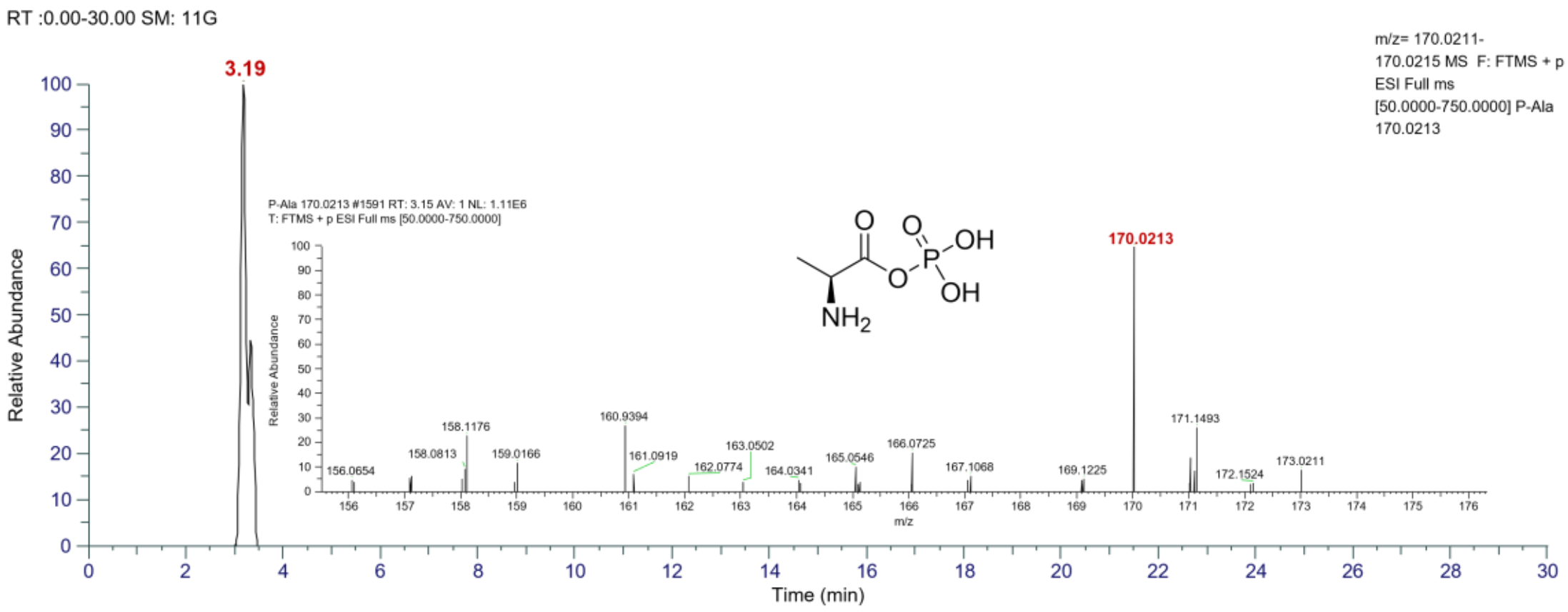

Fig. S57 The extraction ion chromatography-mass spectrometry (EIC-MS) data in positive ion mode of O-phosphoryl Ala. The positive ion mode analysis results confirm that O-phosphoryl Ala was formed in the reaction system containing Ala, CAP, and 0.25 M Mg^{2+}. The extraction ion chromatogram (EIC) shows a peak at m/z 170.0213, corresponding to O-phosphoryl Ala, with a retention time of 3.19 minutes.

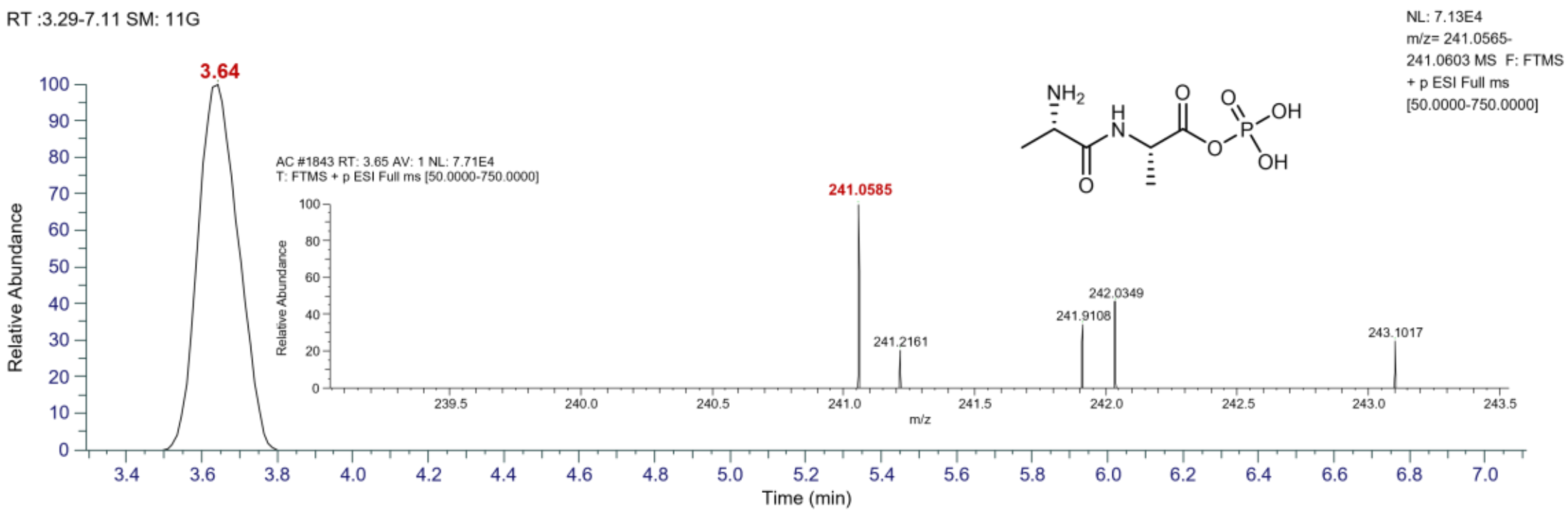

Fig. S58 The extraction ion chromatography-mass spectrometry (EIC-MS) data in positive ion mode of O-phosphoryl Ala_2. The positive ion mode analysis results confirm that O-phosphoryl Ala_2 was formed in the reaction system containing Ala, CAP, and 0.25 M Mg^{2+}. The extraction ion chromatogram (EIC) shows a peak at m/z 241.0585, corresponding to O-phosphoryl Ala_2, with a retention time of 3.46 minutes.

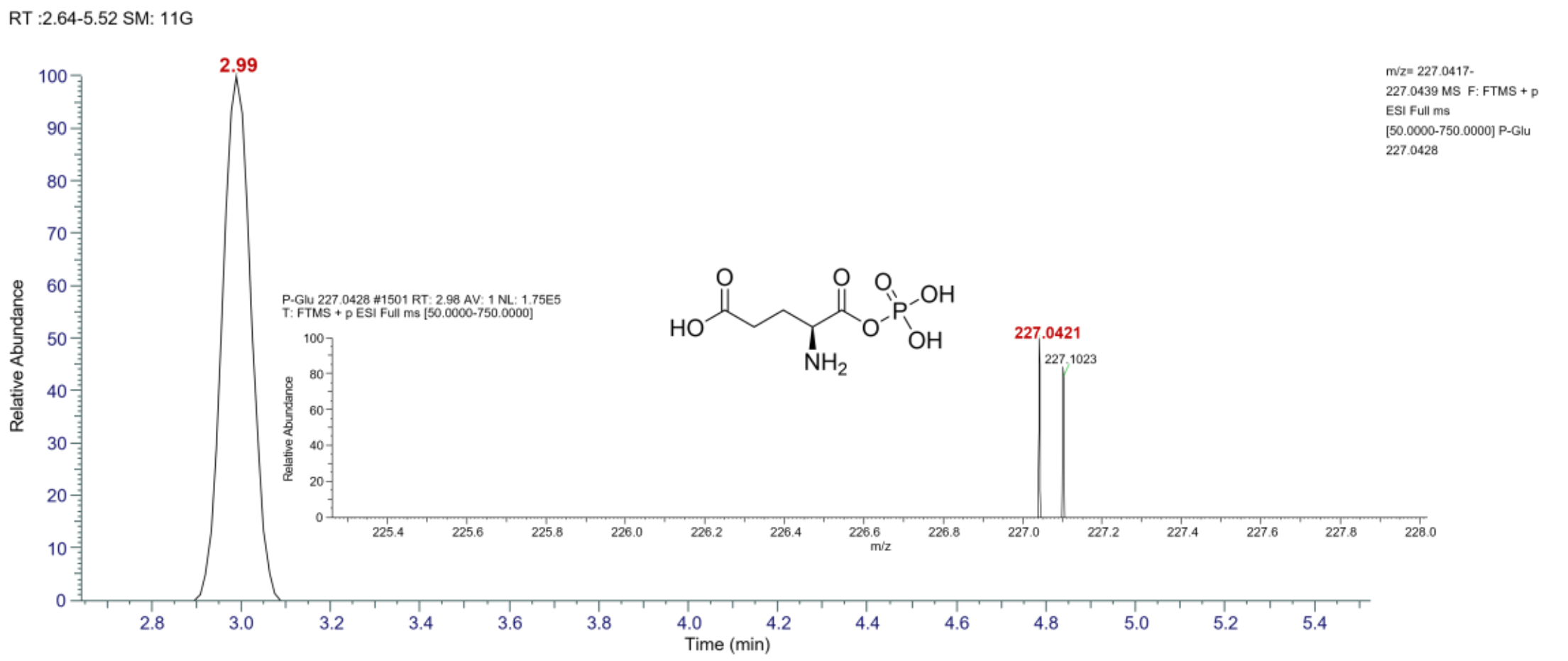

Fig. S59 The extraction ion chromatography-mass spectrometry (EIC-MS) data in positive ion mode of O-phosphoryl Glu. The positive ion mode analysis results confirm that O-phosphoryl Glu was formed in the reaction system containing Glu, CAP, and 0.25 M Mg^{2+}. The extraction ion chromatogram (EIC) shows a peak at m/z 227.0417, corresponding to O-phosphoryl Glu, with a retention time of 2.99 minutes.

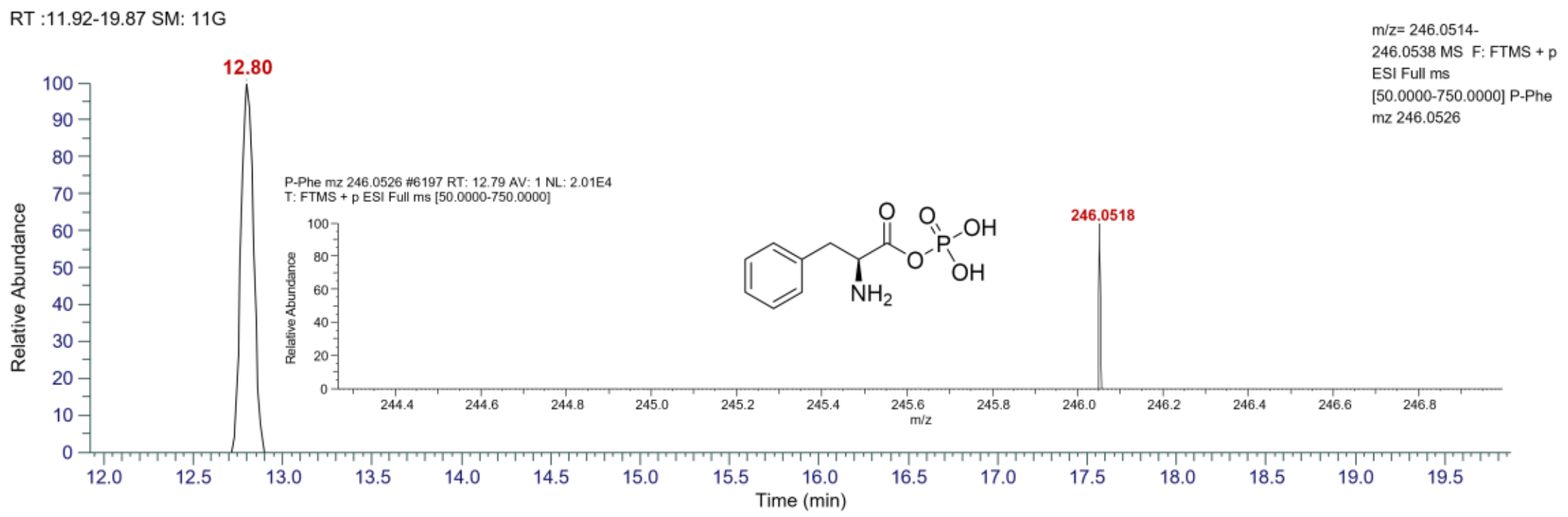

Fig. S60 The extraction ion chromatography-mass spectrometry (EIC-MS) data in positive ion mode of O-phosphoryl Phe. The positive ion mode analysis results confirm that O-phosphoryl Phe was formed in the reaction system containing Phe, CAP, and 0.25 M Mg^{2+}. The extraction ion chromatogram (EIC) shows a peak at m/z 246.0518, corresponding to O-phosphoryl Phe, with a retention time of 12.80 minutes.

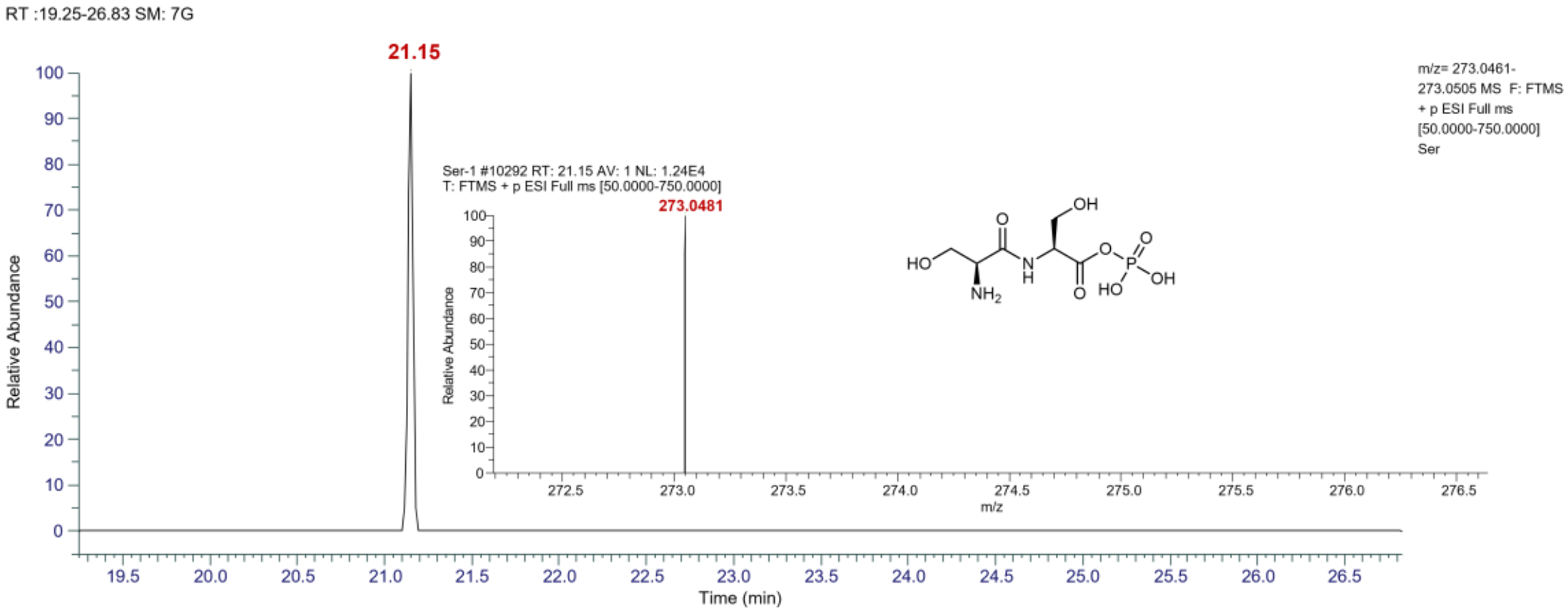

Fig. S61 The extraction ion chromatography-mass spectrometry (EIC-MS) data in positive ion mode of O-phosphoryl Ser$_2$. The positive ion mode analysis results confirm that O-phosphoryl Ser$_2$ was formed in the reaction system containing Ser, CAP, and 0.25 M Mg^{2+}. The extraction ion chromatogram (EIC) shows a peak at m/z 273.0481, corresponding to O-phosphoryl Ser$_2$, with a retention time of 21.15 minutes.

CAP and potassium cyanate (KCNO) promote peptide bond formation

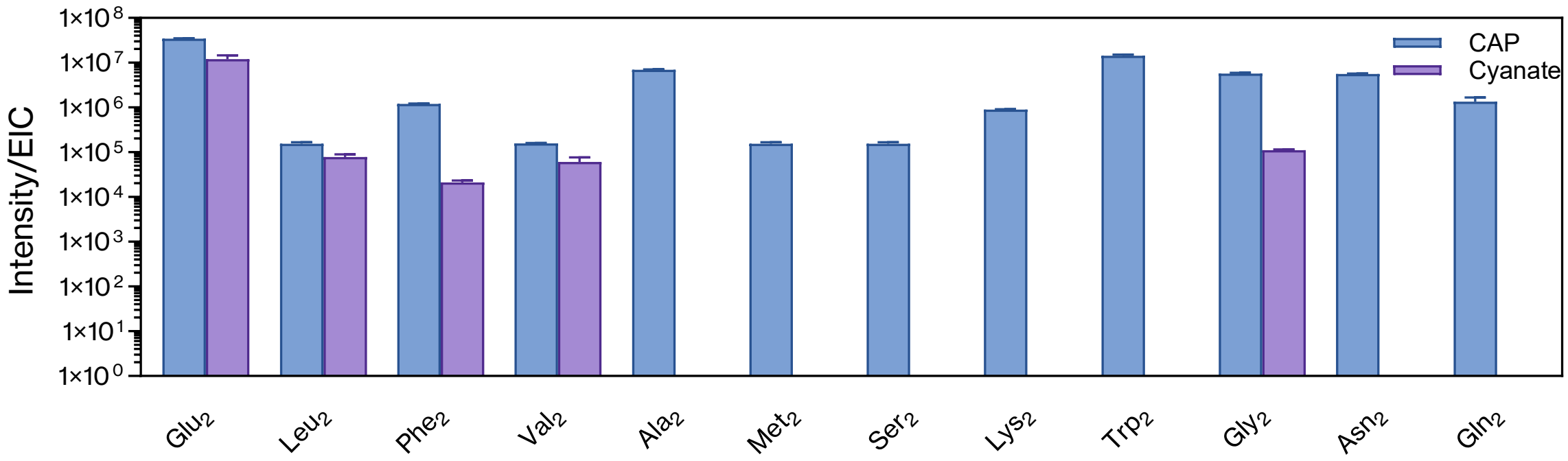

Fig. S62 Compared to potassium cyanate (KCNO) used alone, CAP offers greater advantages in promoting the polymerization of amino acids. EIC intensity of dipeptide formed when each of the 12 amino acids reacts with CAP or KCNO in the presence of 0.25 mM Mg^{2+}.

CAP can be in situ formed with urea and phosphate

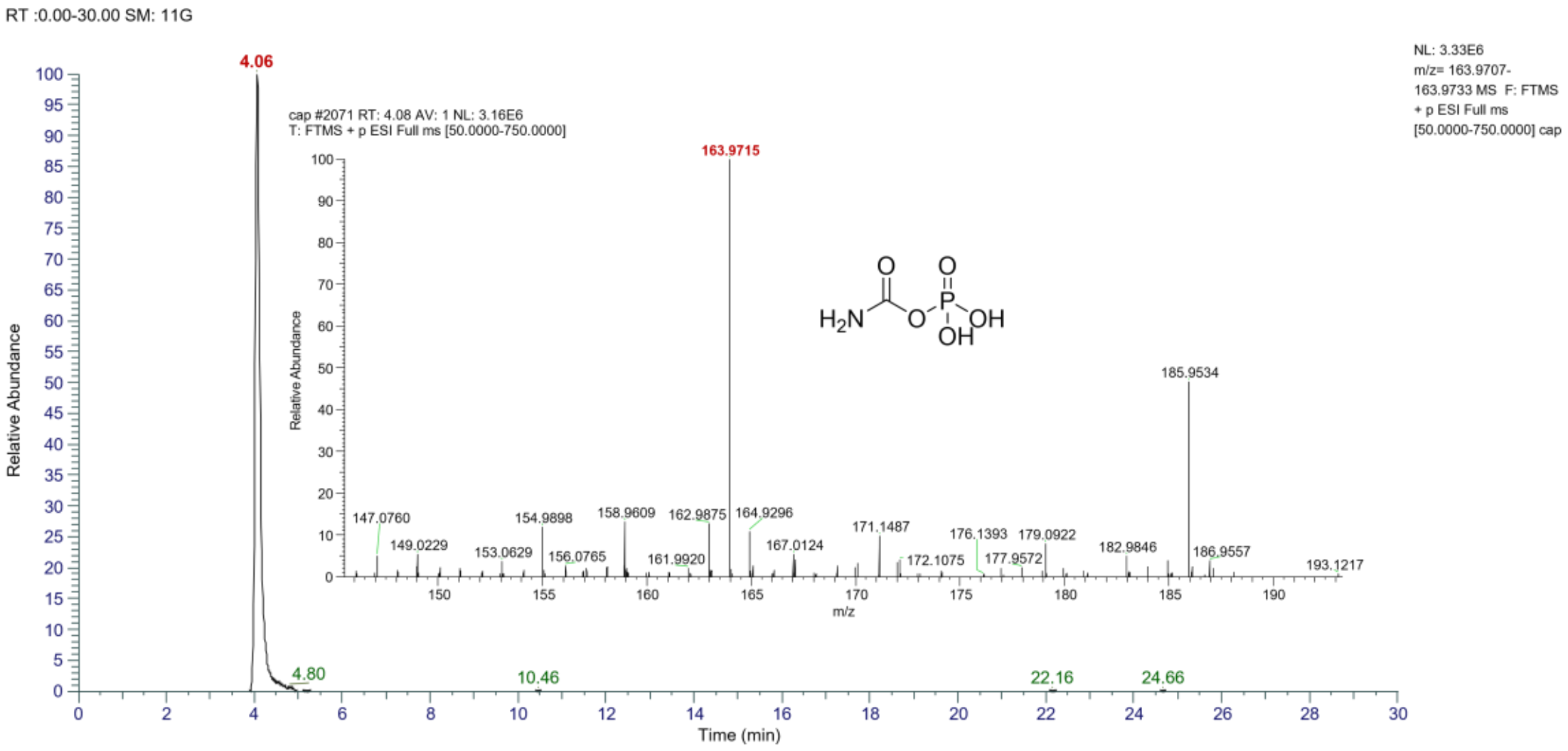

Fig. S63 The extracted ion chromatography-mass spectrometry (EIC-MS) data in positive ion mode for CAP. Analytical results confirmed that CAP was formed in the reaction system containing Urea and Phosphate. The extracted ion chromatogram (EIC) showed a peak at m/z 163.9715 corresponding to CAP, with a retention time of 4.06 minutes.

One-pot peptide synthesis

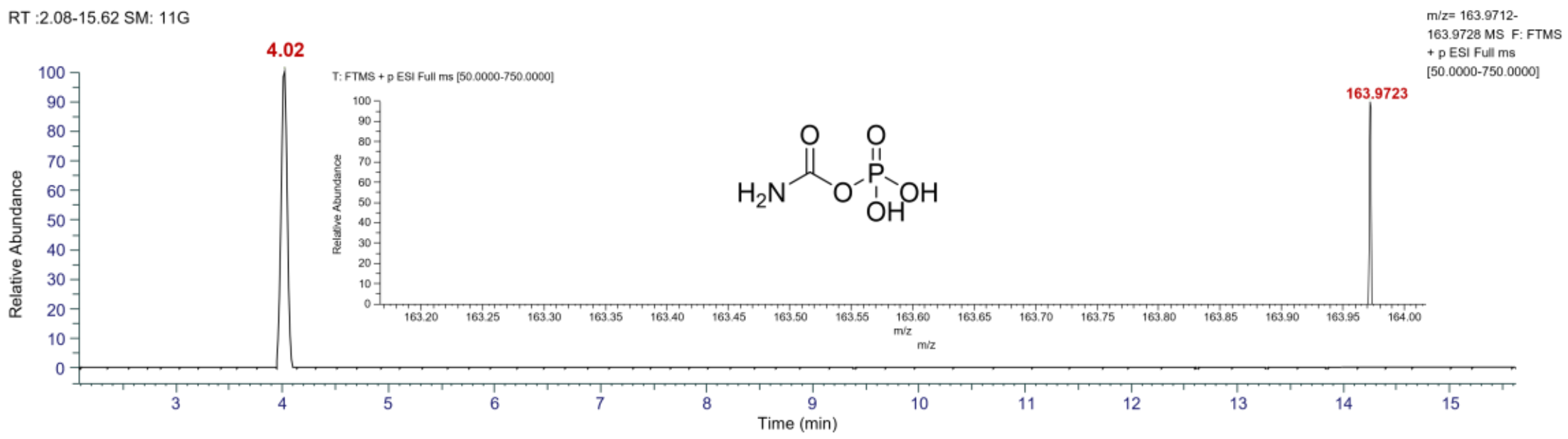

Fig. S64 The extracted ion chromatography-mass spectrometry (EIC-MS) data for CAP. Analytical results confirmed that CAP was generated in the reaction system at 37 °C. The extracted ion chromatogram (EIC) showed a peak at m/z 163.9723 corresponding to CAP, with a retention time of 4.02 minutes.

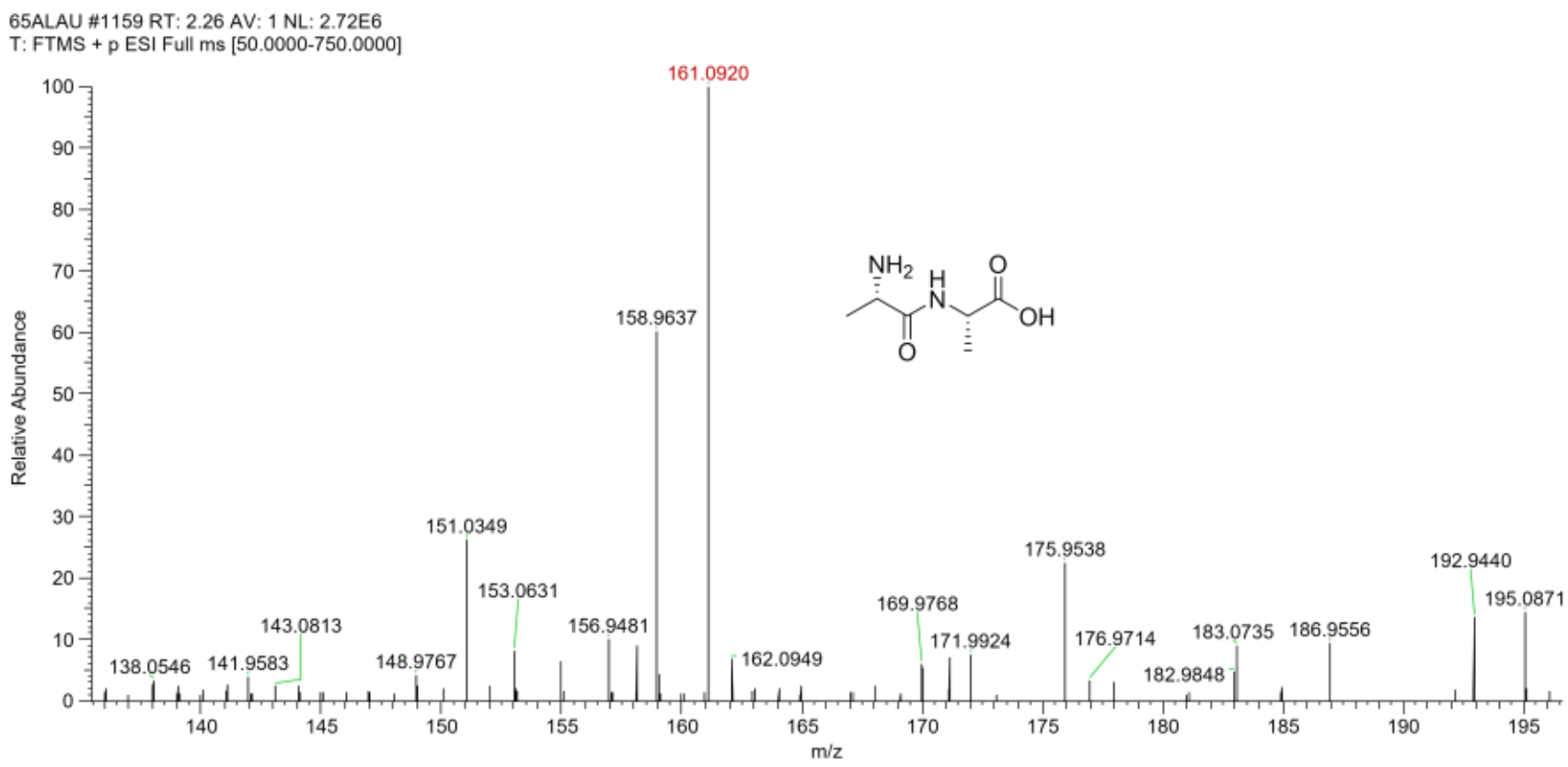

Fig. S65 The extracted ion chromatography-mass spectrometry (EIC-MS) data for Ala₂. The analysis results confirmed that Ala_2 was formed in the reaction system of the one-pot method. The extraction ion chromatogram (EIC) showed a corresponding peak at m/z 161.0920, which corresponded to Ala_2.

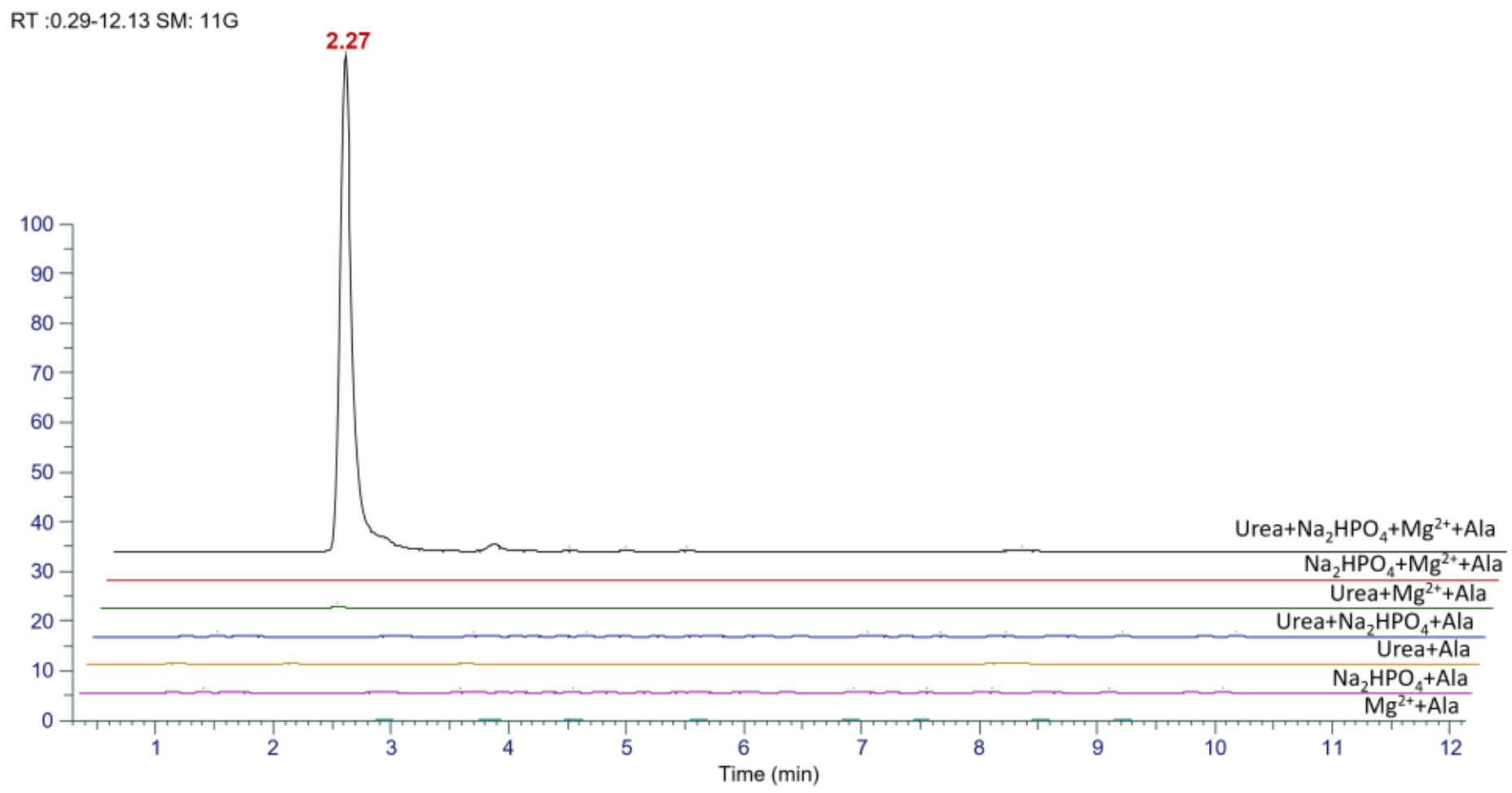

Fig. S66 The extracted ion chromatography-mass spectrometry (EIC-MS) data for Ala₂. Ala_2 can only be formed when Urea, Na_2HPO_4, Mg^{2+} and Ala are present simultaneously.

Table S3: The Ala_2-Intensity/EIC in different systems. Urea, phosphoric acid, and metal ions, respectively, have an impact on the peptide formation of amino acids.

Reactants	Ala_2-Intensity/EIC
Urea+Ala	0
Na_2HPO_4+Ala	0
Mg^{2+}+Ala	0
Urea+Na_2HPO_4+Ala	0
Urea+Mg^{2+}+Ala	0
Na_2HPO_4+Mg^{2+}+Ala	0
Urea+Na_2HPO_4+Mg^{2+}+Ala	1594167

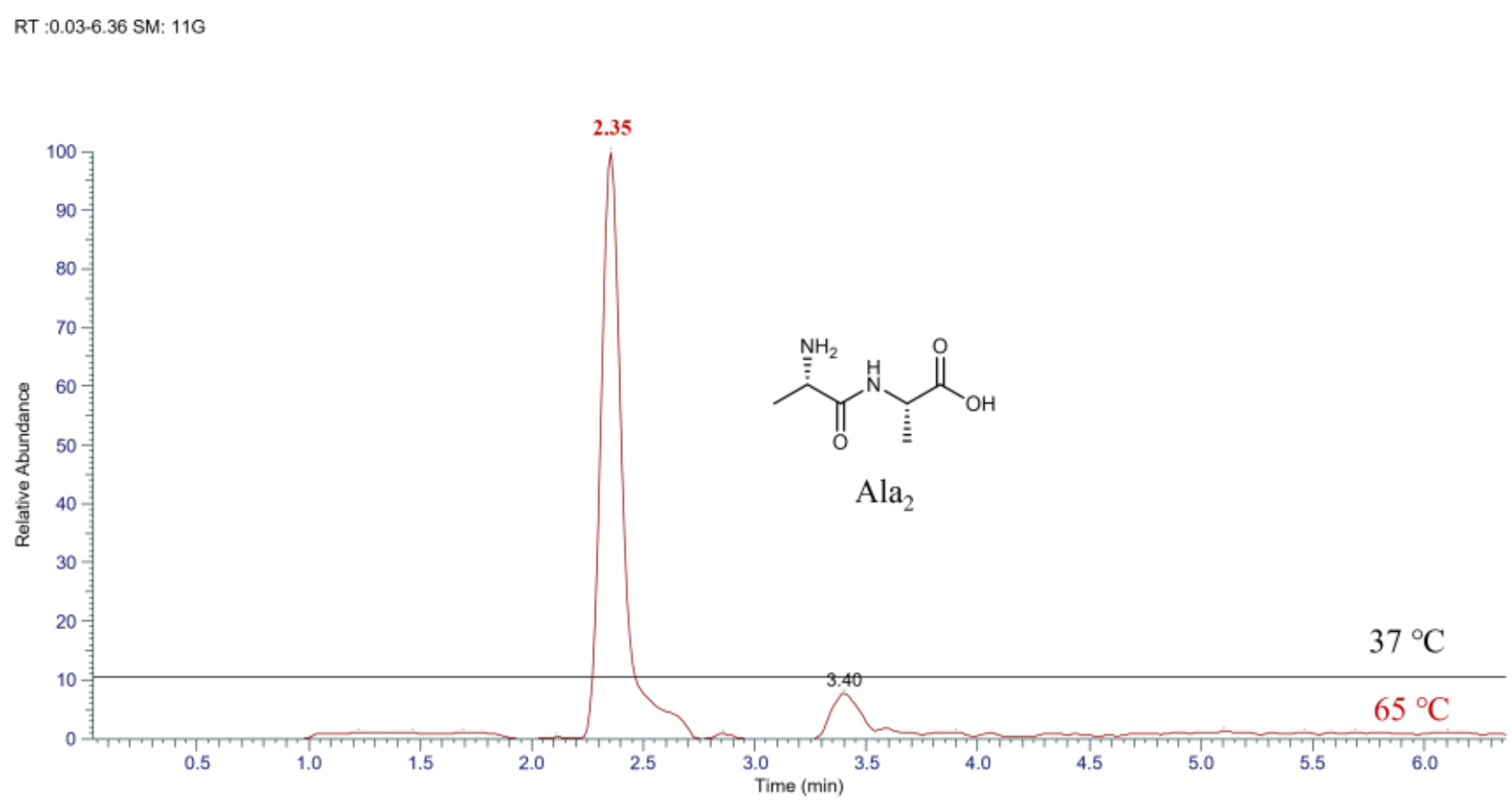

Fig. S67 The extracted ion chromatography-mass spectrometry (EIC-MS) data for Ala_2. A comparison of peptide formation from Urea, Ala, and Mg^{2+} at 37 °C and 65 °C showed that no Ala_2 was produced at 37 °C, whereas a signal for Ala_2 appeared at 65 °C.

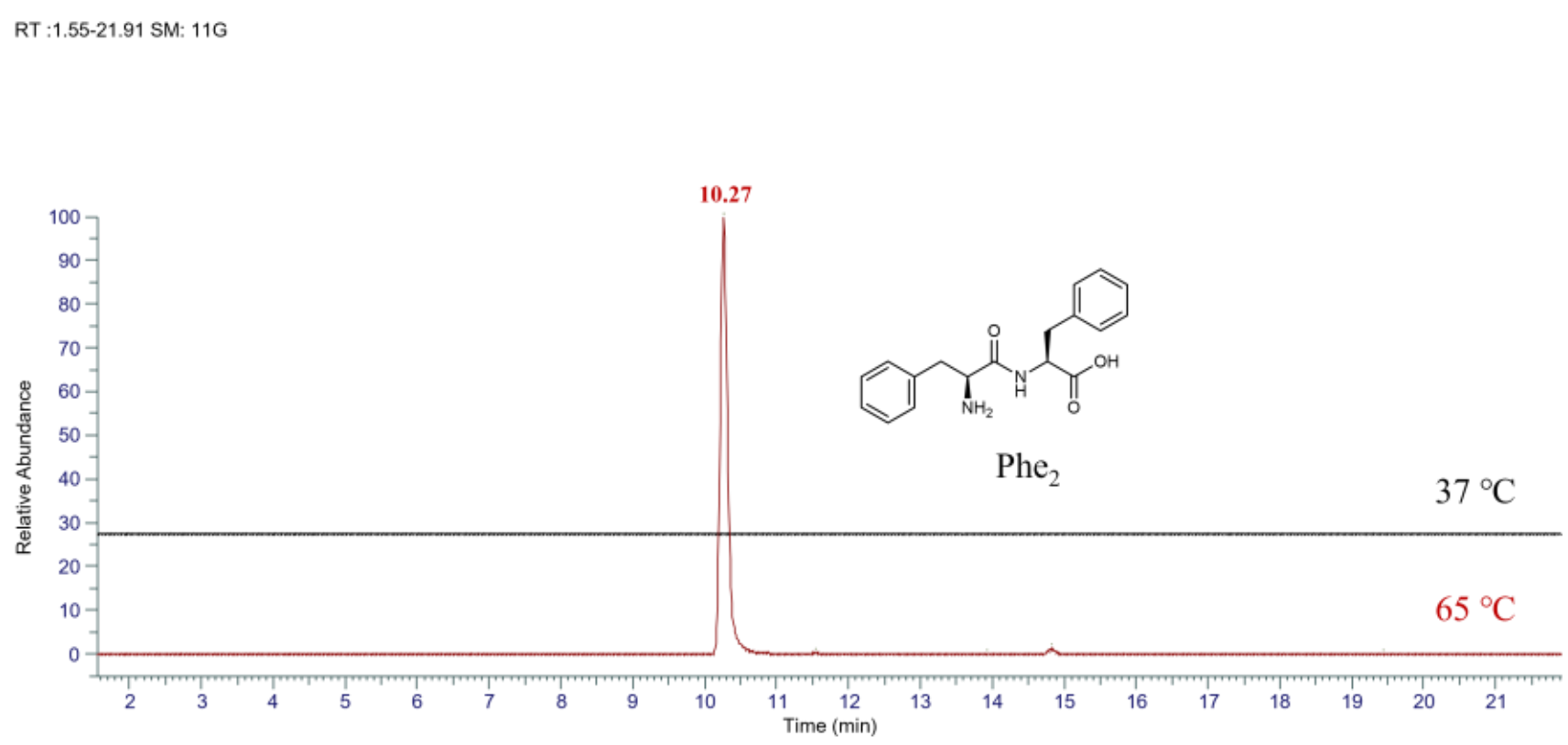

Fig. S68 The extracted ion chromatography-mass spectrometry (EIC-MS) data for $_2$. A comparison of peptide formation from Urea, Phe, and Mg^{2+} at 37 °C and 65 °C showed that no Phe_2 was produced at 37 °C, whereas a signal for Phe_2 appeared at 65 °C.

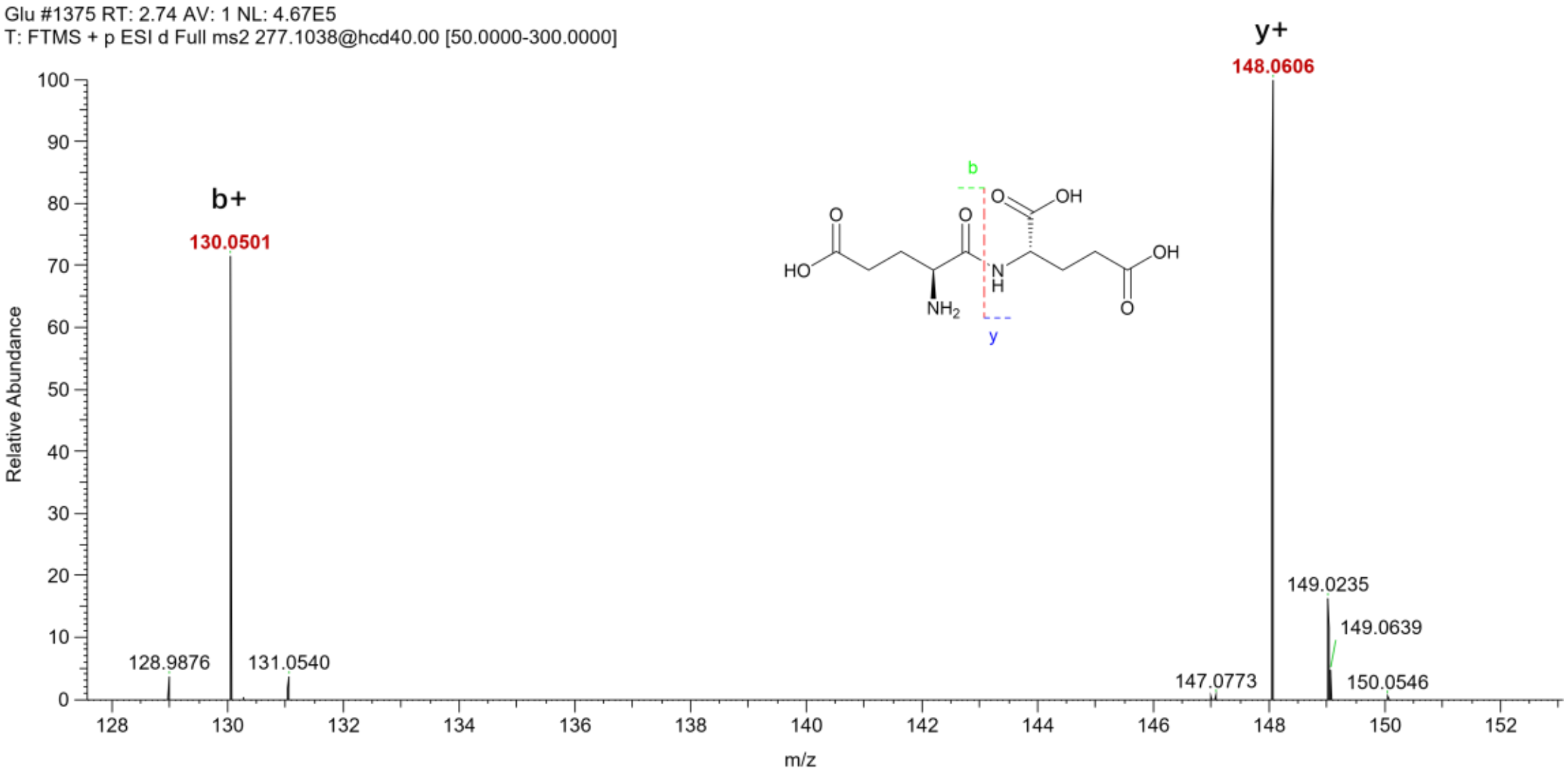

Fig. S69 The extraction ion chromatography-mass spectrometry and MS/MS spectroscopy of the Glu_2. MS² analysis of m/z 277.1034 revealed characteristic fragment ions, including corresponding a- and x-type series. The analytical data confirm the formation of the Glu_2 product in the reaction.

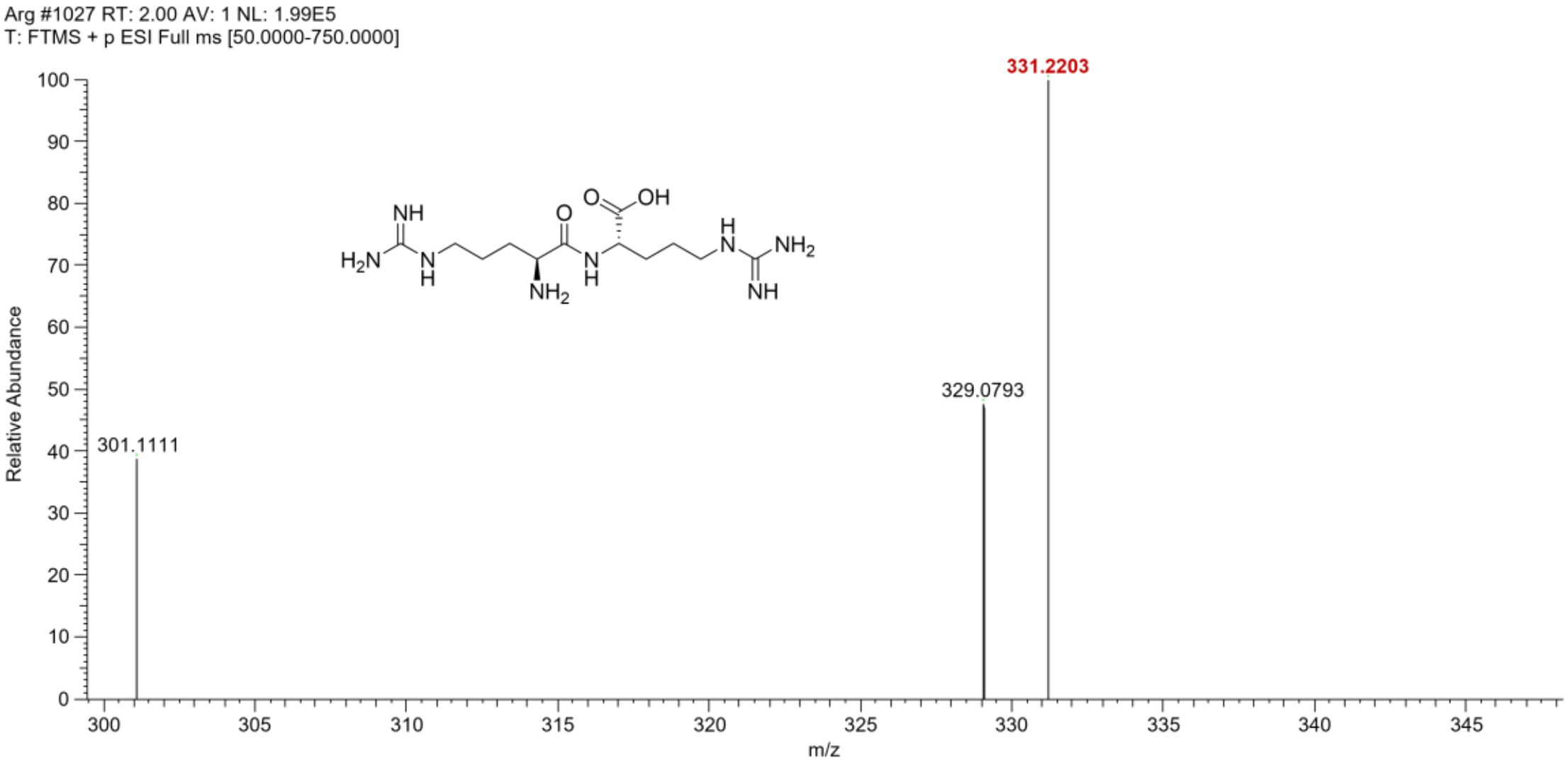

Fig. S70 The extracted ion chromatography-mass spectrometry (EIC-MS) data for Arg_2. The analysis results confirmed that Arg_2 was formed in the reaction system of the one-pot method. The extraction ion chromatogram (EIC) showed a corresponding peak at m/z 331.2203, which corresponded to Arg_2.

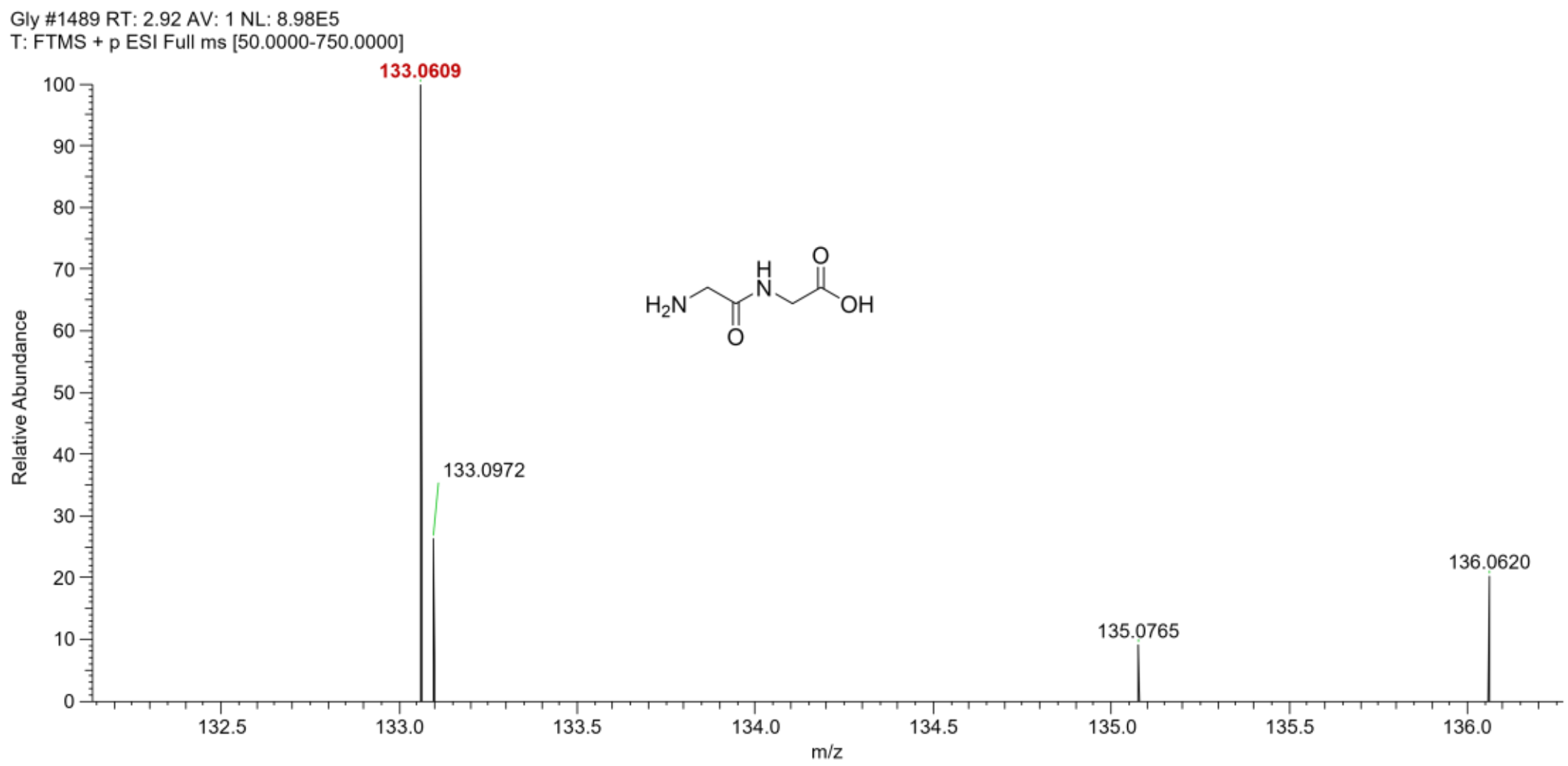

Fig. S71 The extracted ion chromatography-mass spectrometry (EIC-MS) data for Gly_2. The analysis results confirmed that Gly_2 was formed in the reaction system of the one-pot method. The extraction ion chromatogram (EIC) showed a corresponding peak at m/z 133.0609, which corresponded to Gly_2.

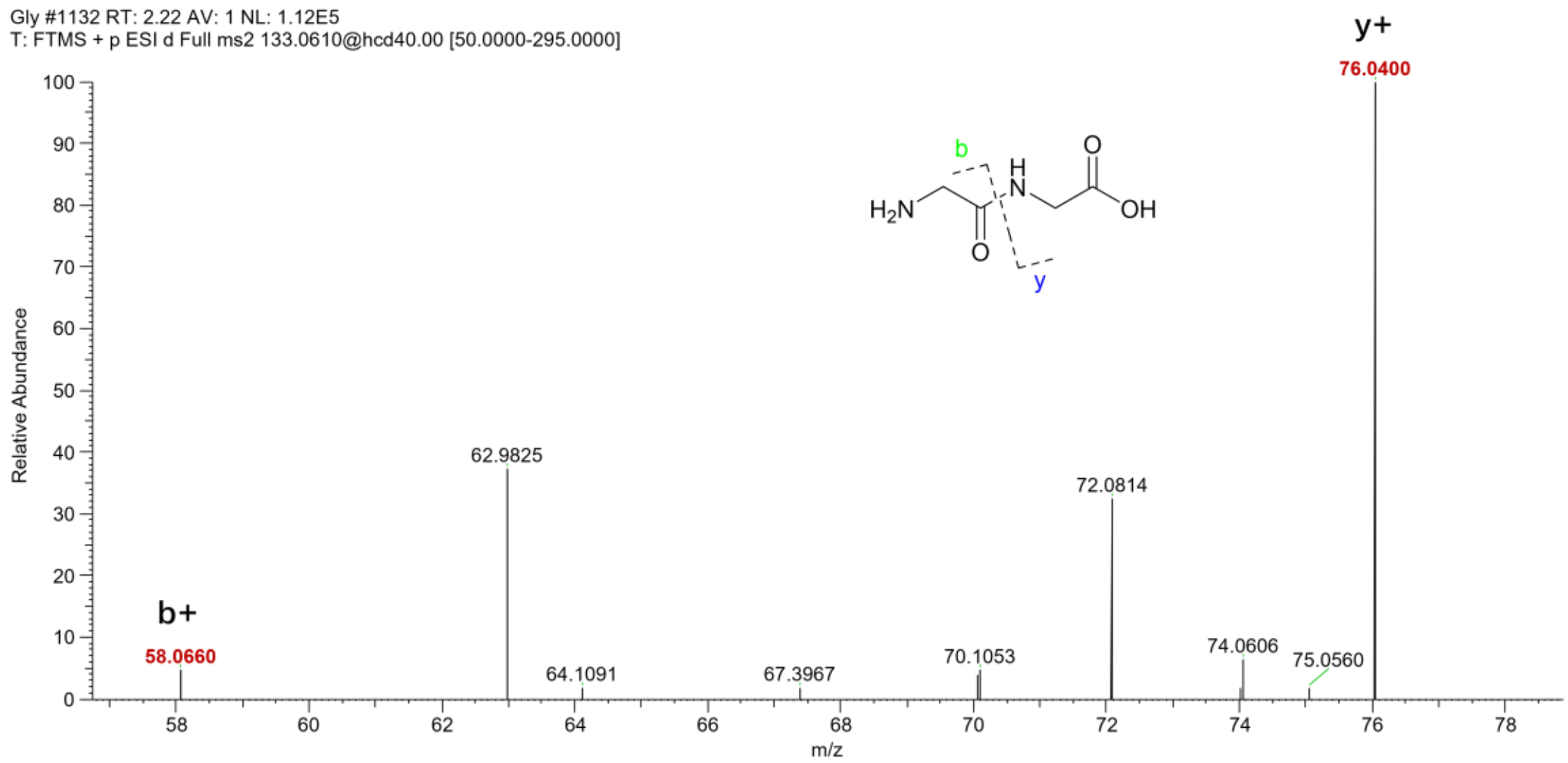

Fig. S72 The Extraction ion chromatography-mass spectrometry and MS/MS spectroscopy of the Gly_2. MS^2 analysis of m/z 133.0609 revealed characteristic fragment ions, including corresponding a- and x-type series. The analytical data confirm the formation of the Gly_2 product in the reaction.

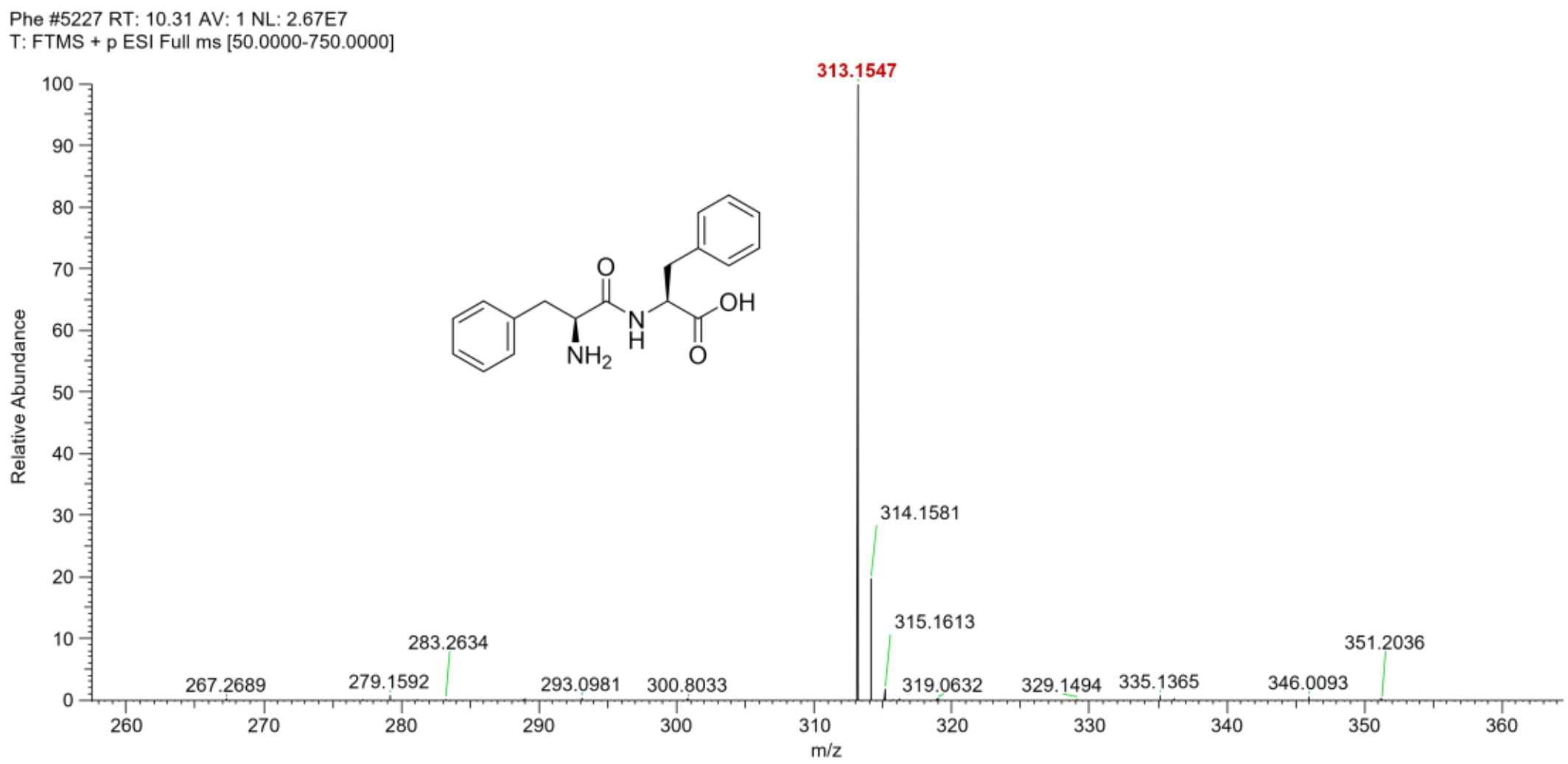

Fig. S73 The extracted ion chromatography-mass spectrometry (EIC-MS) data for Phe_2. The analysis results confirmed that Phe_2 was formed in the reaction system of the one-pot method. The extraction ion chromatogram (EIC) showed a corresponding peak at m/z 313.1547, which corresponded to Phe_2.

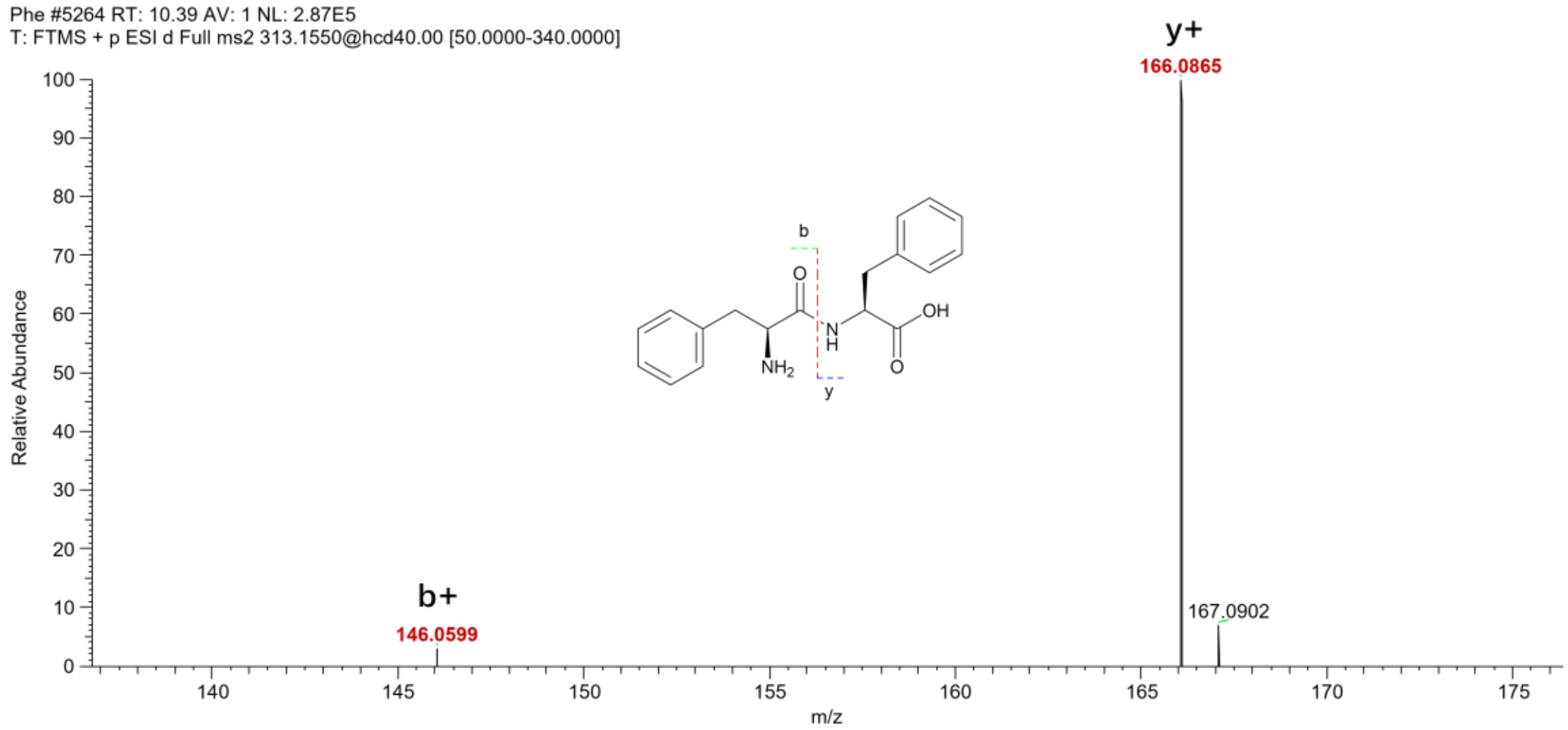

Fig. S74 The Extraction ion chromatography-mass spectrometry and MS/MS spectroscopy of the Phe_2. MS² analysis of m/z 313.1547 revealed characteristic fragment ions, including corresponding a- and x-type series. The analytical data confirm the formation of the Phe_2 product in the reaction.

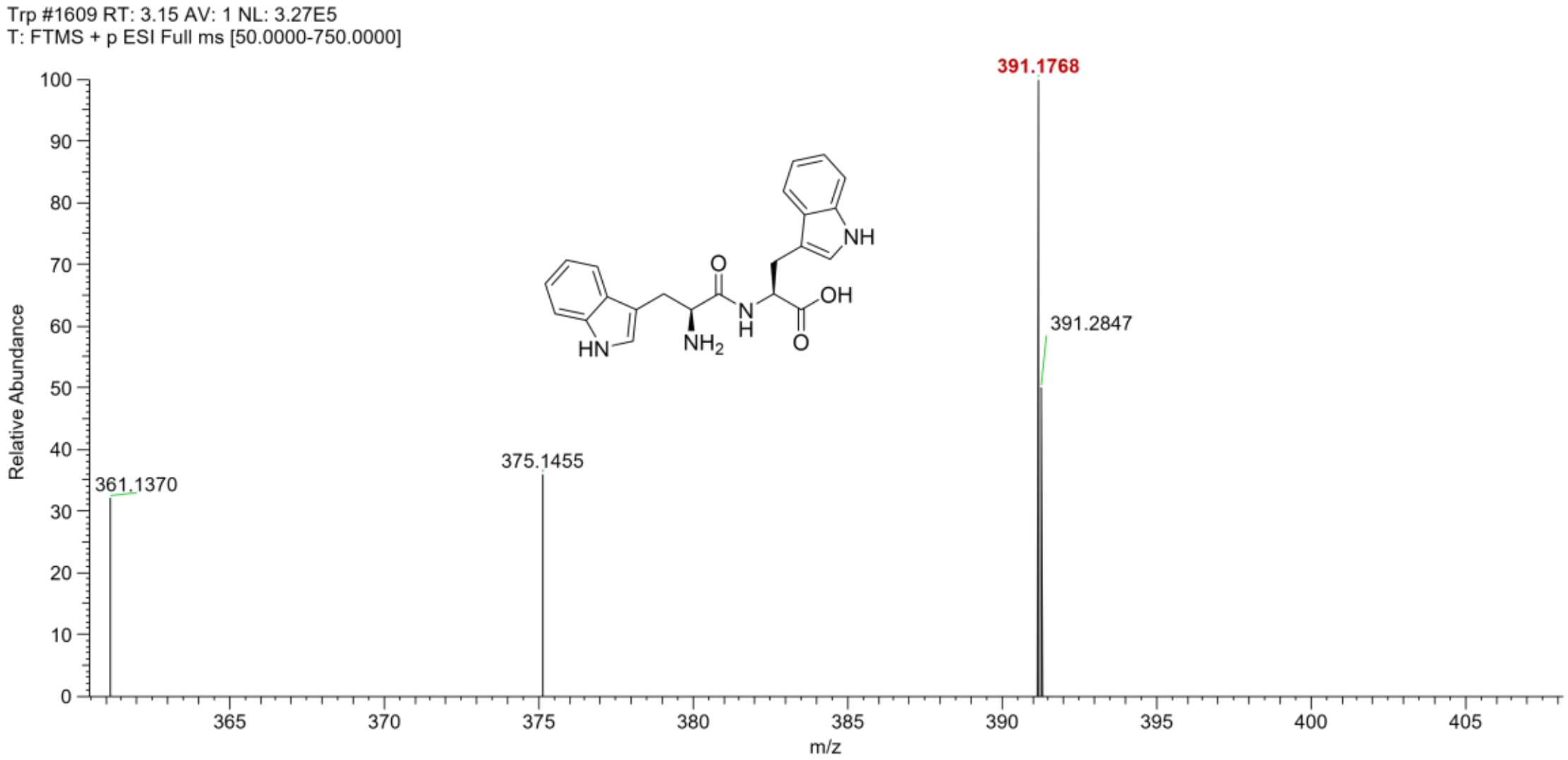

Fig. S75 The extracted ion chromatography-mass spectrometry (EIC-MS) data for Trp₂. The analysis results confirmed that Trp_2 was formed in the reaction system of the one-pot method. The extraction ion chromatogram (EIC) showed a corresponding peak at m/z 391.1768, which corresponded to Trp_2.

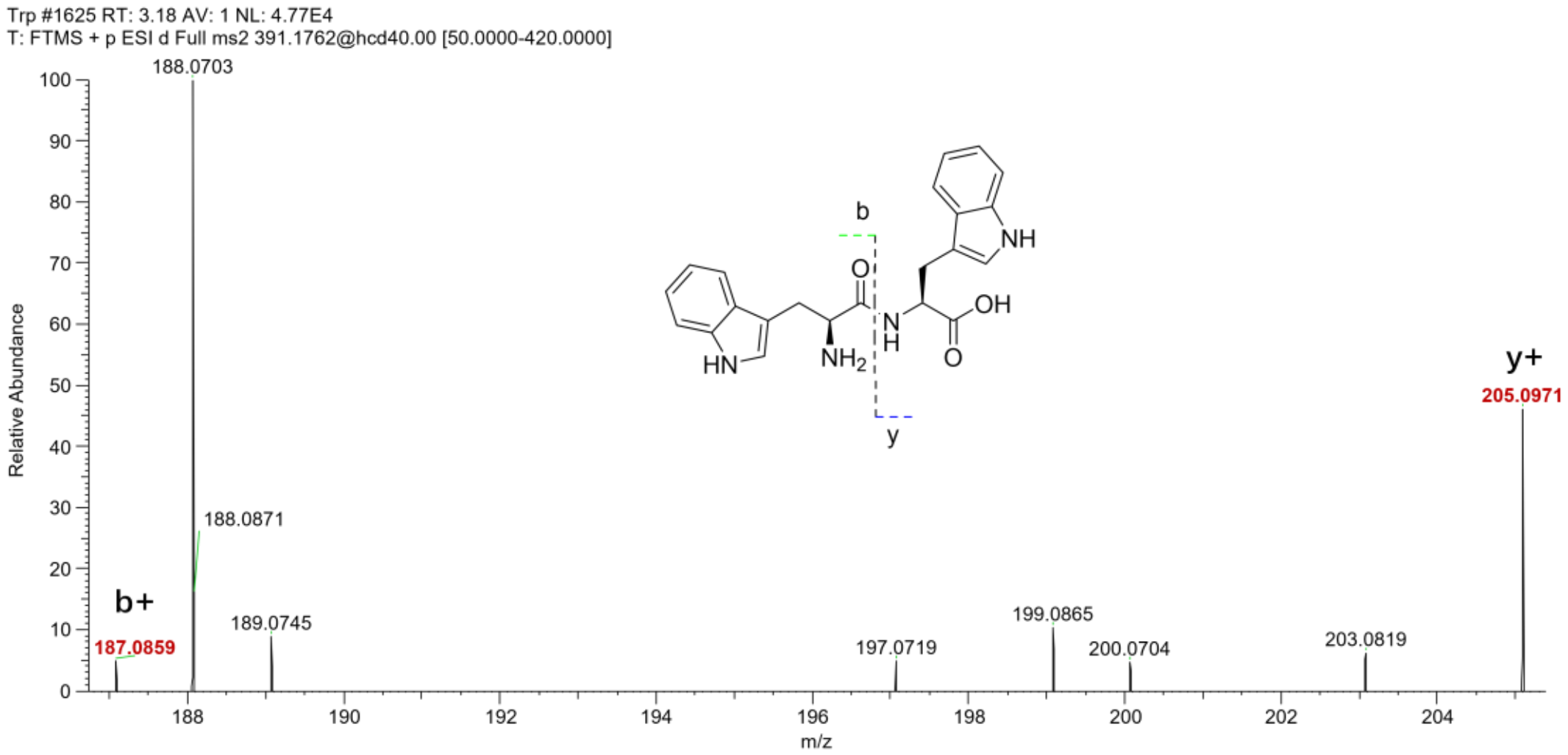

Fig. S76 The Extraction ion chromatography-mass spectrometry and MS/MS spectroscopy of the Trp_2. MS^2 analysis of m/z 391.1768 revealed characteristic fragment ions, including corresponding a- and x-type series. The analytical data confirm the formation of the Trp_2 product in the reaction.

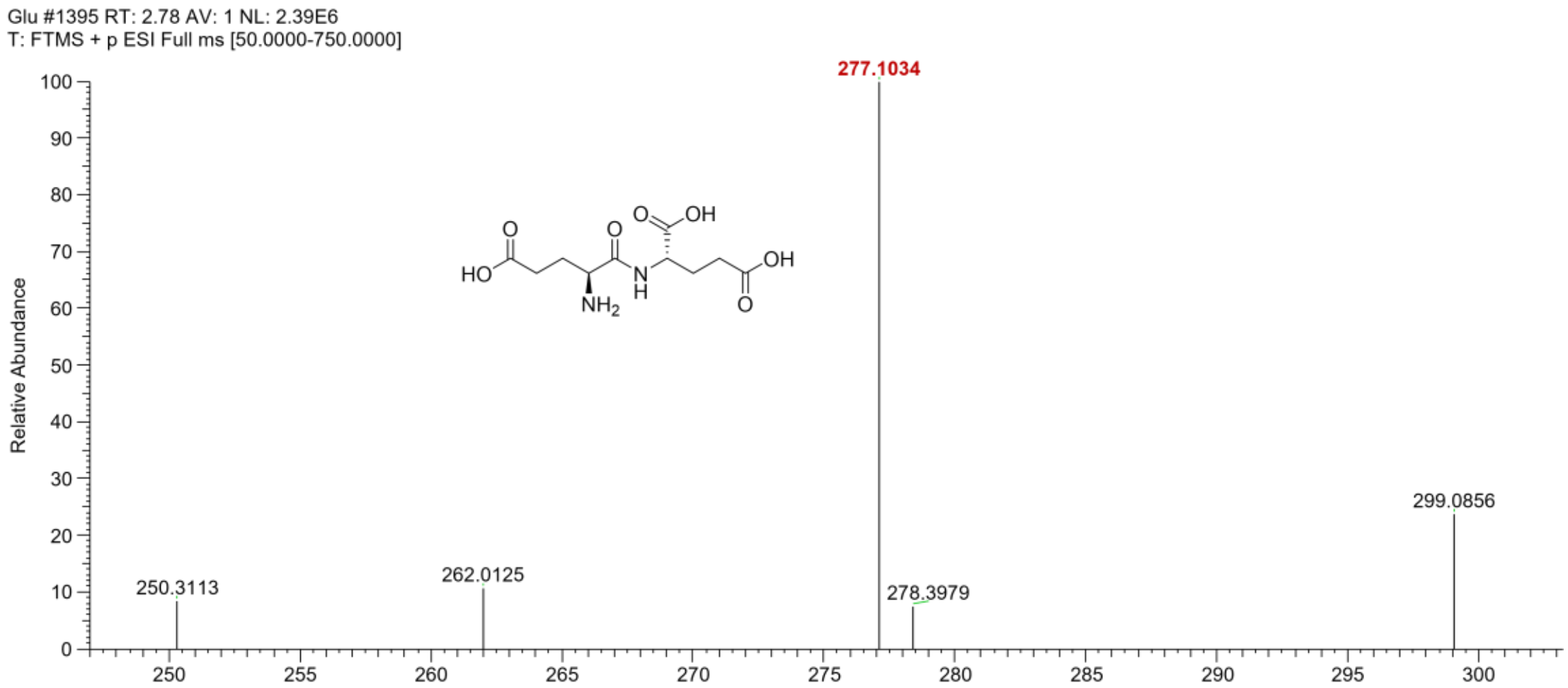

Fig. S77 The extracted ion chromatography-mass spectrometry (EIC-MS) data for Glu_2. The analysis results confirmed that Glu_2 was formed in the reaction system of the one-pot method. The extraction ion chromatogram (EIC) showed a corresponding peak at m/z 277.1034, which corresponded to Glu_2.

The influence of EDTA on reaction systems

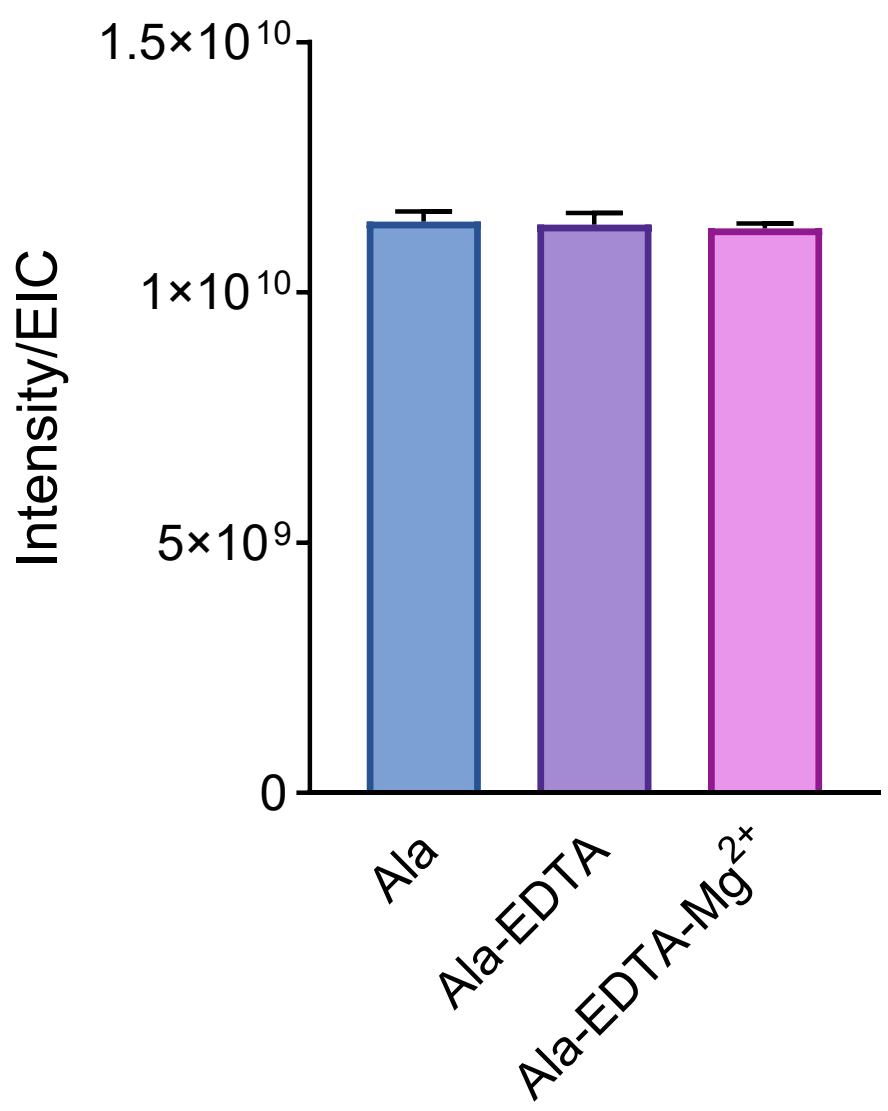

Fig. S78 The effect of EDTA on alanine. 0.02M of alanine, 0.001M of Mg2+, and 0.0023M of EDTA were divided into three groups: Ala, Ala-EDTA, and Ala-EDTA-Mg2+. They were left for the same period of time, and then analyzed by HPLC-MS to monitor and compare the differences in amino acid intensities.

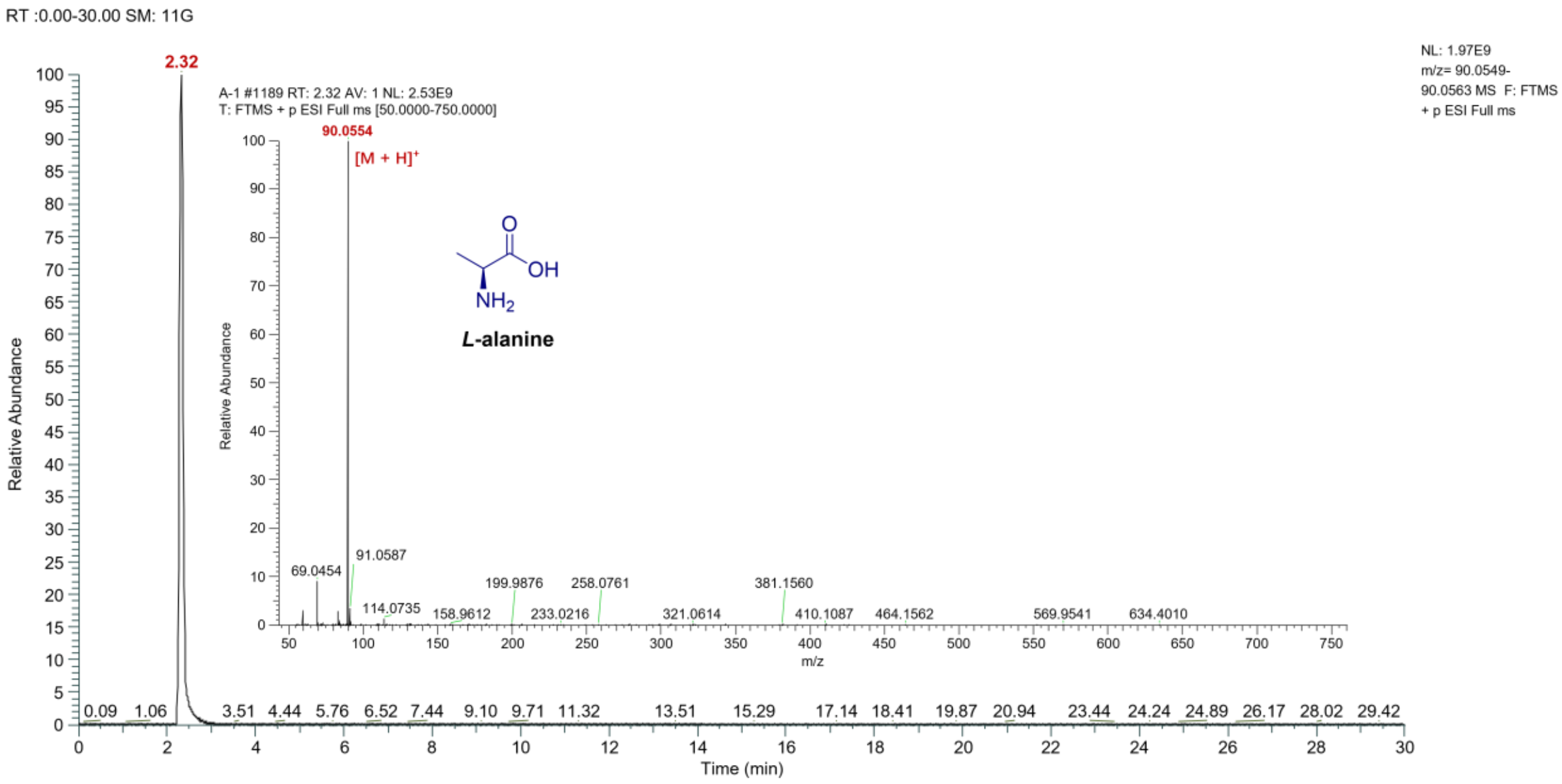

Fig. S79 Analysis of alanine by extraction ion chromatography-mass spectrometry. The analytical data confirm the extraction ion chromatography-mass spectrum of alanine. The extracted ion chromatogram with m/z 90.0550 was analyzed, which corresponds to alanine. The retention time is 2.32 minutes.

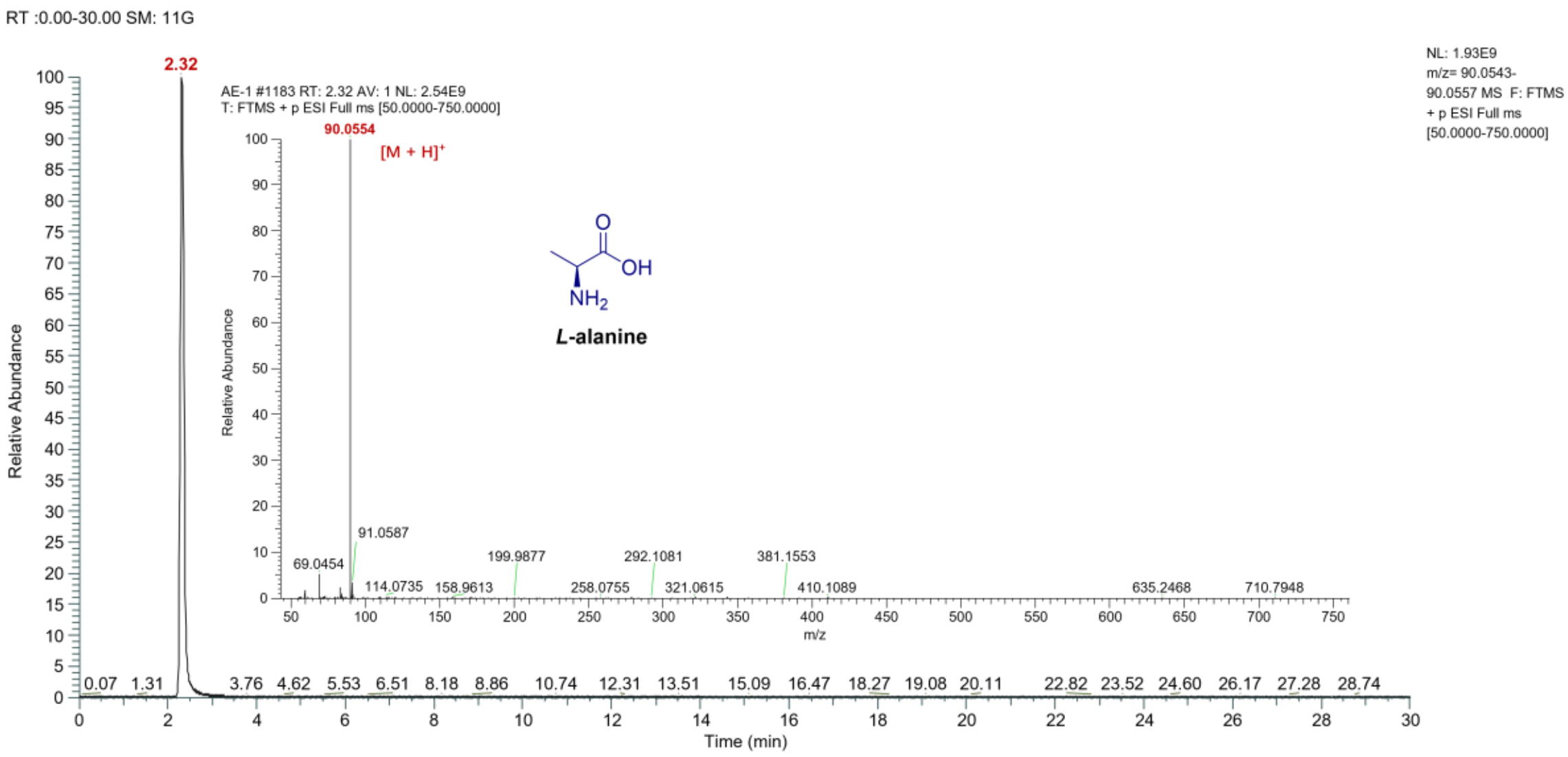

Fig. S80 Analysis of alanine by extraction ion chromatography-mass spectrometry. The analytical data confirm the extraction ion chromatography-mass spectrum of alanine and EDTA. The extracted ion chromatogram with m/z 90.0550 was analyzed, which corresponds to alanine. The retention time is 2.32 minutes.

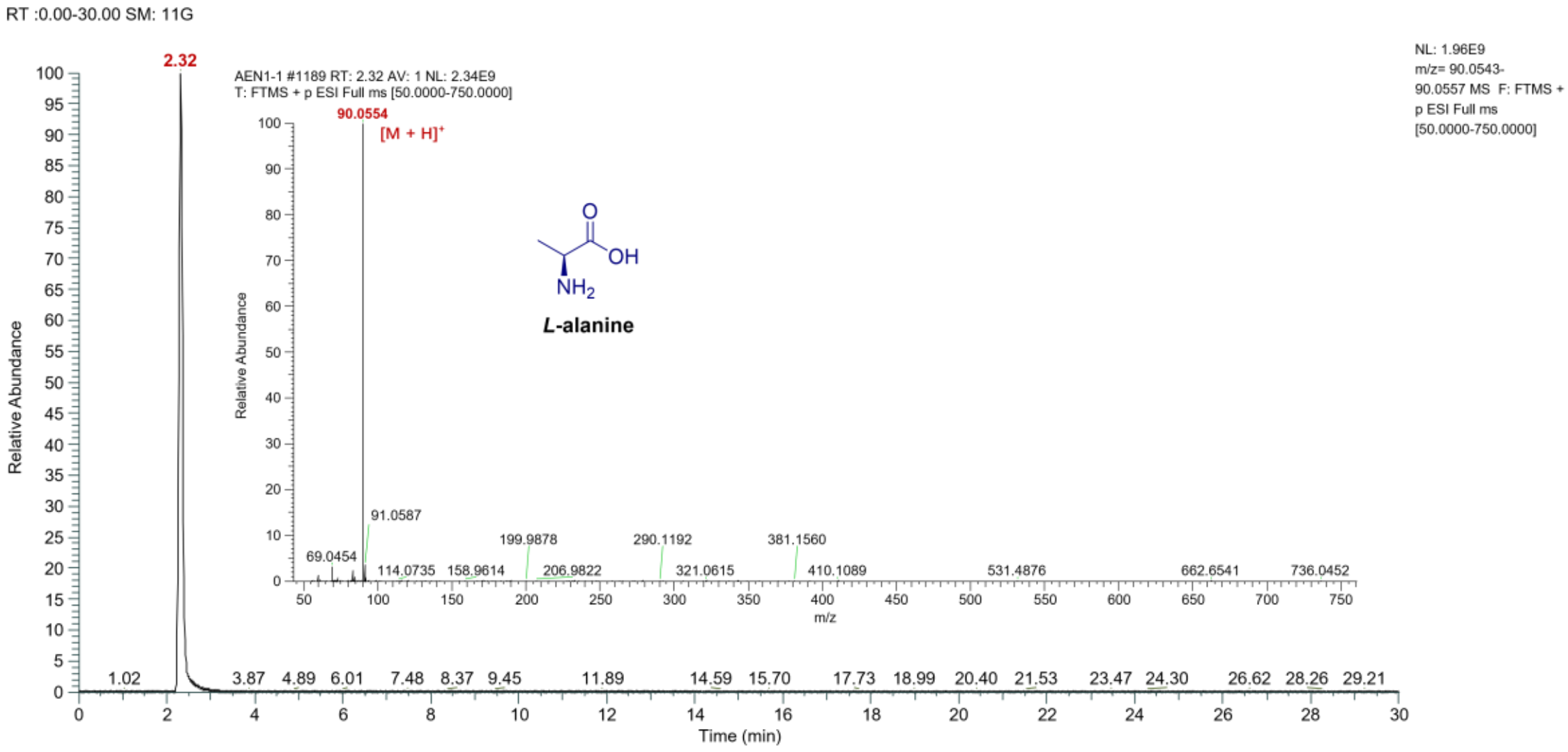

Fig. S81 Analysis of alanine by extraction ion chromatography-mass spectrometry. The analytical data confirm the extraction ion chromatography-mass spectrum of alanine, EDTA and Mg^{2+}. The extracted ion chromatogram with m/z 90.0550 was analyzed, which corresponds to alanine. The retention time is 2.32 minutes.

Supplementary Data

Table S4: Original data for Fig 2A.

Metal Ion	Intensity/EIC of Ala_2 (n = 3)		
Ctrl	0	0	0
Mg^{2+}	6,757,169	7,080,984	6,966,279
Fe^{3+}	6,242,002	6,333,946	6,445,107
Fe^{2+}	783,036	721,159	771,108
Ni^{2+}	0	0	0
Mn^{2+}	0	0	0
Zn^{2+}	0	0	0
Cu^{2+}	0	0	0
Ba^{2+}	0	0	0

Table S5: Original data for Fig 2B.

pH	Intensity/EIC of Ala_2 (n = 3)		
3	1,545,807	1,745,807	1,833,472
4	2,036,978	2,253,697	2,835,751
5	6,898,965	6,534,395	6,054,762
6	8,932,541	8,698,425	7,934,250
7	4,596,327	4,847,525	5,373,148

Table S6: Original data for Fig 2C.

Mg^{2+} concentration (mM)	Intensity/EIC of Ala_2 (n = 3)		
0.10	4,857,654	4,654,896	4,407,557
0.25	7,339,896	7,297,536	7,522,421
0.50	7,267,655	7,233,714	7,022,476
1.00	6,834,395	6,898,965	6,412,575
2.00	4,477,843	4,399,334	4,834,427

Table S7: Original data for Fig 2D.

Amino acid	Dipeptide (n = 3)			Tripeptide (n = 3)		
Ser	156,321	145,695	165,922			
Leu	154,689	169,489	144,109	6,901	8,254	6,672
Val	161,845	158,834	152,484			
Lys	912,582	892,326	902,595	11,732	13,751	15,786
Phe	1,223,047	1,197,823				
Gln	1,615,734	1,023,699	1,456,228	4,136	5,734	4,357
Met	3,424,045	3,285,263	3,569,255			
Asn	5,754,617	5,489,625	5,587,632	14,921,424	12,456,757	12,578,686
Gly	5,962,160	5,765,252	5,462,628	405,447	423,722	392,457
Ala	6,757,169	7,080,984	6,966,279	122,085	154,756	113,695
Trp	14,602,408	13,595,262	14,959,522			
Glu	33,758,395	35,575,460	34,389,733	52,828	43,278	40,274
