# Supplementary material for: Carbamoyl phosphate-mediated peptide formation in mild prebiotic environment
Source: Commun Chem. 2026 Jul 23;9:361. doi: 10.1038/s42004-026-02149-6 (PMC13760053; doi:10.1038/s42004-026-02149-6)
Supplement: Supplementary file 2 [file 42004_2026_2149_MOESM2_ESM.pdf]

Description of Additional Supplementary Files:

File: Supplementary Data

Description: The source data for Figures 2
